# Hydride bridge in [NiFe]-hydrogenase observed by nuclear resonance vibrational spectroscopy

**DOI:** 10.1038/ncomms8890

**Published:** 2015-08-10

**Authors:** Hideaki Ogata, Tobias Krämer, Hongxin Wang, David Schilter, Vladimir Pelmenschikov, Maurice van Gastel, Frank Neese, Thomas B. Rauchfuss, Leland B. Gee, Aubrey D. Scott, Yoshitaka Yoda, Yoshihito Tanaka, Wolfgang Lubitz, Stephen P. Cramer

**Affiliations:** 1 Max Planck Institute for Chemical Energy Conversion, Mülheim an der Ruhr 45470, Germany; 2 Department of Chemistry, University of California, Davis, California 95616, USA; 3 Division of Physical Biosciences, Lawrence Berkeley National Laboratory, Berkeley, California 94720, USA; 4 Department of Chemistry, University of Illinois, Urbana, Illinois 61801, USA; 5 Institut für Chemie, Technische Universität Berlin, Berlin 10623, Germany; 6 Division of Research and Utilization, SPring-8/JASRI, Hyogo 679-5198, Japan; 7 Materials Dynamics Laboratory, RIKEN SPring-8, Hyogo 679-5148, Japan

## Abstract

The metabolism of many anaerobes relies on [NiFe]-hydrogenases, whose characterization when bound to substrates has proven non-trivial. Presented here is direct evidence for a hydride bridge in the active site of the ^57^Fe-labelled fully reduced Ni-R form of *Desulfovibrio vulgaris* Miyazaki F [NiFe]-hydrogenase. A unique ‘wagging’ mode involving H^−^ motion perpendicular to the Ni(*μ*-H)^57^Fe plane was studied using ^57^Fe-specific nuclear resonance vibrational spectroscopy and density functional theory (DFT) calculations. On Ni(*μ*-D)^57^Fe deuteride substitution, this wagging causes a characteristic perturbation of Fe–CO/CN bands. Spectra have been interpreted by comparison with Ni(*μ*-H/D)^57^Fe enzyme mimics [(dppe)Ni(*μ*-pdt)(*μ*-H/D)^57^Fe(CO)_3_]^+^ and DFT calculations, which collectively indicate a low-spin Ni(II)(*μ*-H)Fe(II) core for Ni-R, with H^−^ binding Ni more tightly than Fe. The present methodology is also relevant to characterizing Fe–H moieties in other important natural and synthetic catalysts.

## Abstract (web summary)

Understanding the catalytic mechanism of redox-active hydrogenases is a key to efficient hydrogen production and consumption. Here, the authors use nuclear resonance vibrational spectroscopy to study [NiFe]-hydrogenase, and observe a bridging hydride structure in an EPR silent intermediate.

## Graphical abstract

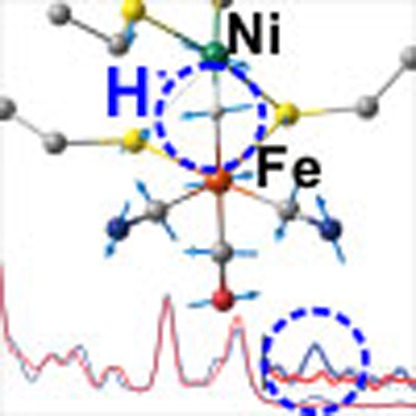

A central goal of the hydrogen economy is to forestall a continual buildup of CO_2_ and the threat of global climate change^1^. However, achieving independence of carbon-based fuels necessitates the development of better H_2_-processing catalysts from earth-abundant materials. In this regard, bidirectional hydrogenase enzymes, which catalyse both production and consumption of H_2_ (refs 2, 3), have attracted interest either for direct utilization^4^ or as aspirational targets for biomimetic catalysts^5^,^6^,^7^,^8^,^9^,^10^,^11^.

The redox-active hydrogenases are classified as either [FeFe]- or [NiFe]-hydrogenase, according to the metals present at their active sites. While both types possess high catalytic activity^12^,^13^,^14^, the latter are attractive practically in that they are more O_2_-tolerant^12^,^15^. The [NiFe]-hydrogenase active site features Ni and Fe centres bridged by two Cys residues (Fig. 1a), with two further Cys ligands binding Ni terminally, and the Fe coordination sphere being completed by one CO and two CN^−^ groups. The [NiFe] site functions in concert with an electron transport chain comprising three Fe–S clusters. The catalytic cycle is generally thought to involve three key redox states of the bimetallic centre: electron spin (paramagnetic) resonance (EPR)-silent Ni-SI_a_, EPR-active Ni-C and another EPR-silent species known as Ni-R^16^,^17^,^18^. Despite progress in characterizing hydrogenases by crystallography, spectroscopy, and theory^16^, questions remain about the molecular and electronic structure of various intermediates and inhibited species. This particularly applies to the electronic structure of Ni-R.

Up to three isoelectronic Ni(II)Fe(II) forms of Ni-R are characterized by their pH-dependent Fourier transform infrared (FT-IR) signatures^18^. The structures proposed for these Ni-R subspecies (Fig. 1b–d) most commonly have a bridging hydride at the active site (Ni(*μ*-H)Fe)^19^,^20^,^21^,^22^, with some even featuring an additional (terminal) hydride at Ni (HNi(*μ*-H)Fe)^23^,^24^. Another suggested form has an Fe-bound dihydrogen ligand (NiFe(*η*^2^-H_2_))^25^,^26^. A recent high-resolution crystallographic analysis of Ni-R1, a subspecies of Ni-R, indicates a Ni(*μ*-H)Fe core with a protonated terminal cysteinate^27^. Among proposals supporting the bridging hydride, there is further debate as to whether H^−^ is bound more strongly to Fe or Ni (ref. 28) and whether Ni(II) is high^29^ or low spin^30^ or whether both spin configurations coexist in the bulk^31^,^32^.

The Ni-R state represents a special challenge for spectroscopy in that it is EPR-silent, photolabile (and thus difficult to study with Raman spectroscopy) and expected to feature an active site M–H moiety (being notoriously difficult to observe using infrared methods)^33^. The present study instead employs a synchrotron radiation technique called nuclear resonance vibrational spectroscopy (NRVS), which involves X-ray excitation of a Mössbauer-active nuclide^34^,^35^,^36^,^37^. The raw NRVS data are commonly translated into partial vibrational density of states (PVDOS) spectra^38^, which show vibrational energy contributions specifically from the Mössbauer-active nuclei, such as ^57^Fe. PVDOS can also be predicted using density functional theory (DFT) or empirical force field calculations, assisting in confident spectral assignments.

NRVS is uniquely suited to detailed investigations of ^57^Fe-labelled enzyme active sites, avoiding interference from the thousands of protein modes present in a typical infrared or Raman spectrum. NRVS has enabled the observation of Fe–CN and Fe–CO bending and stretching modes for the active sites in [NiFe]-hydrogenases^39^,^40^, despite the presence of 11 (or more) Fe centres in clusters of the electron transport chain. This is because Fe–CN and Fe–CO modes are strongest in the region from 440 to 640 cm^−1^, while Fe–S cluster modes only make significant contributions below 440 cm^−1^ (refs 41, 42, 43). Another recent application of NRVS to a *trans*-H/D-^57^Fe-(CO) compound shed light on coupling of Fe–H/D and Fe–CO bending modes^33^.

The present study discloses the first spectroscopic evidence for the bridging hydride in the Ni-R active site and the unprecedented NRVS observation of a Fe–H stretching mode in a synthetic Ni–H–Fe system. A combined experimental/theoretical analysis of both Ni-R and its synthetic mimics is presented here, an approach that we anticipate to be of broad utility for the characterization of (bio)inorganic Fe-hydride catalysts.

## Results

### NRVS of model complexes

**Figure 2:**
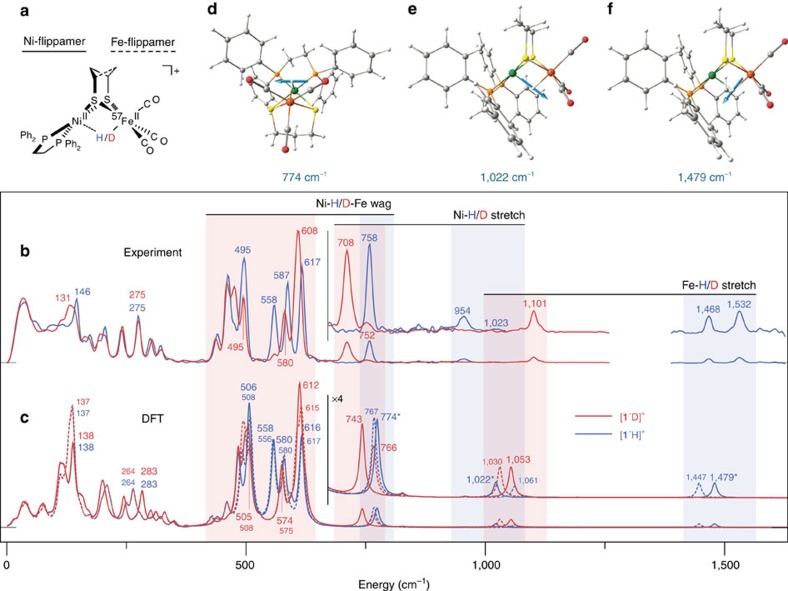
Metal-hydride bands for complexes [1′H/D]^+^. (**a**) Structure of [**1**′H]^+^ showing ‘flippamer’ conformations of the pdt^2−^ ligand. (**b**,**c**) Full-range ^57^Fe PVDOS for [**1**′H]^+^ (blue trace) and [**1**′D]^+^ (red trace) from NRVS experiments (**b**) and DFT calculations (**c**). In **c**, the plain/broken traces are spectra calculated for the dominant/alternative Ni-/Fe-flippamer, respectively. Spectra are repeated in the region >700 cm^−1^ with their intensities × 4 amplified. The colour bars highlight specific M–H/D bands, as well as their shifts on isotopic substitution. (**d**–**f**) Scaled-arrow representations of the M–H normal modes calculated for the Ni-flippamer of [**1**′H]^+^ are shown, with the corresponding bands indicated (*) in **c**. Unscaled-arrow and animated representations of these M–H modes can be found in Supplementary Fig. 22 and Supplementary Movies 1,2,3, respectively.

The Ni(II)(*μ*-thiolate)_2_(*μ*-H)Fe(II) core proposed for Ni-R is reproduced by the diamagnetic H_2_-evolving catalyst [(dppe)Ni(*μ*-pdt)(*μ*-H)Fe(CO)_3_]^+^ ([**1**H]^+^, dppe=1,2-bis(diphenylphosphino)ethane=1,2-Ph_2_PCH_2_CH_2_P Ph_2_, pdt^2−^=1,3-propanedithiolate=^−^SCH_2_CH_2_CH_2_S^−^)^44^,^45^ shown in Fig. 2a, which has recently been studied using resonance Raman spectroscopy^46^. Synthetic methodology allowed the preparation of the labelled analogue [(dppe)Ni(*μ*-pdt)(*μ*-H/D)^57^Fe(CO)_3_]^+^ in both hydride [**1′**H]^+^ and deuteride [**1′**D]^+^ forms for ^57^Fe NRVS analysis (see Supplementary Figs 1–16 and Supplementary Note 1). Complementary ^13^CO-labelled species [**1′′**H/D]^+^ were prepared as well.

The NRVS spectra for [**1′**H/D]^+^ are presented in Fig. 2b. Apart from low-frequency (180–340 cm^−1^) Fe–S modes, there are strong bands in the 440–630 cm^−1^ region assigned to *ν*_Fe–CO_ stretches and *δ*_Fe–CO_ bends (Supplementary Table 1). On the high-energy side, NRVS analysis of [**1′**H]^+^ also reveals peaks at 1,532 and 1,468 cm^−1^ that have previously been assigned to *ν*_Fe–H_ modes of two different conformations of the pdt^2−^ ligand (‘Ni/Fe-flippamers’^47^,^48^,^49^,^50^, see Fig. 2a and Supplementary Fig. 17 using Raman spectroscopy on [**1**H]^+^ (ref. 46, Supplementary Table 2). As expected, these features red-shift to ∼1,101 cm^−1^ on D substitution in the bridge. While NRVS has uncovered *δ*_Fe–H/D_ (ref. 33) and *ν*_Fe–D_ modes^51^ for other species, the 1,532 and 1,468 cm^−1^ bands for [**1′**H]^+^ are the first Fe–H stretching modes detected using this technique, as well as being the highest-frequency bands observed using NRVS to date. The band at 954 cm^−1^ for [**1′**H]^+^, previously assigned to a *ν*_Ni–H_ mode^46^, is evidently also Fe-coupled, given its detection using NRVS. In [**1′**D]^+^, the corresponding Ni-D stretch is at 708 cm^−1^. As expected from a harmonic oscillator in which H/D binds a much heavier nucleus, the *ν*_Fe–H_/*ν*_Fe–D_ and *ν*_Ni–H_/*ν*_Ni-D_ frequency ratios are ∼2^**½**^. This difference in the H/D and ^57^Fe nuclear masses also results in pure hydride bands having low NRVS intensities, as such vibrations involve only small displacements of the ^57^Fe centre. In addition, our successful use of NRVS to detect the well-defined *ν*_Ni-D_ stretch in ^57^Fe-labelled [**1′**D]^+^ contrasts Raman studies of natural Fe abundance [**1**D]^+^, in which the Ni-D band was obscured by solvent modes^46^.

On the basis of previous studies, one would expect *δ*_OC–Fe–H/D_ modes to appear in the 530–750/410–640 cm^−1^ regions, respectively^33^,^46^. The hydride [**1′**H]^+^ exhibits a well-defined NRVS feature at 758 cm^−1^ consistent with a *δ*_Fe–H_ mode, this region being obscured by solvent bands in Raman data^46^. As will become clear, this 758 cm^−1^ mode observed for [**1′**H]^+^ is of particular relevance to the interpretation of NRVS data for [NiFe]-hydrogenase. A distinct assignment for the corresponding *δ*_Fe-D_ mode in [**1′**D]^+^ is prevented by its mixing with the Fe–CO bending modes, such that the NRVS intensity is redistributed throughout the 440–630 cm^−1^ region.

Analysis of [**1**′H]^+^ revealed several intense Fe–CO bands in the 440–630 cm^−1^ region (Fig. 2b), the energies of which are almost identical to those of the conjugate base (dppe)Ni(*μ*-pdt)^57^Fe(CO)_3_ (**1**′)^47^, which lacks the hydride bridge. This is exemplified by the *δ*_Fe–CO_ NRVS triplets for [**1**′H]^+^ (558, 587 and 617 cm^−1^) and **1**′ (557, 588 and 613 cm^−1^) being virtually coincident (Supplementary Table 1), suggesting that the presence of H^−^ does not significantly perturb the *δ*_Fe–CO_ dynamics. In contrast, the *δ*_Fe–CO_ region for [**1**′D]^+^ collapses to a pair of bands at 580 and 608 cm^−1^, consistent with significant coupling to the *δ*_Fe-D_ bending motion. Thus, although Fe–D modes have intrinsically low NRVS intensity, the Fe–D/Fe–CO coupling allows for the high-intensity *δ*_Fe–CO_ region to serve as an indicator of whether or not a Fe–D moiety is present^33^.

### DFT of model complexes

DFT calculations were undertaken to better understand the dynamics of H/D-coupled motions in the model complexes. The DFT-simulated ^57^Fe PVDOS of [**1′**H/D]^+^ Ni/Fe-flippamers were compared with NRVS data over the range 0–1,600 cm^−1^ (Fig. 2c, see also Supplementary Fig. 18 and Supplementary Tables 1 and 2). As with our recently reported spectra for [**1′**]^0/+^ (ref. 52), the observed and calculated band positions and intensities are in good agreement below 700 cm^−1^. Given that the broad >700 cm^−1^ region is solely populated by Ni/Fe–H/D bands, DFT also allows for a confident assignment of these NRVS features (Fig. 2b,c) despite their low intensities and the difficulties recognized in the accurate theoretical prediction of M–H vibrational frequencies^46^.

In line with previous DFT calculations and Raman analyses^46^, the key distinction between calculated NRVS data for the two [**1′**H]^+^ Ni/Fe-flippamers results from splitting of the *ν*_Fe–H_=1,479/1,447 cm^−1^ and *ν*_Ni–H_=1,022/1,061 cm^−1^ modes, respectively, as indicated in Fig. 2c. For the Ni-flippamer, the calculated *ν*_Fe/Ni–H_ modes are shown correspondingly in Fig. 2f,e and Supplementary Movies 1 and 2. As expounded in the ‘Further Discussion on Model Complex’ section of the Supplementary Discussion, a fine yet noticeable interplay of the optimized Ni/Fe–H distances (Supplementary Table 3) gives rise to the *inverted* character of these ∼30–40 cm^−1^ flippamer-dependent splittings.

One of the most interesting and useful results from the present calculations on [**1′**H]^+^ is the prediction of a ^57^Fe PVDOS band at 774/767 cm^−1^ for the Ni/Fe-flippamers, respectively. With only a small flippamer-dependent splitting of 7 cm^−1^, this mode gives rise to the most intense feature above 700 cm^−1^ and aligns well with the NRVS band observed at 758 cm^−1^ (Fig. 2b,c). Inspection of the DFT-calculated nuclear displacements (see Fig. 2d and Supplementary Movie 3 for the mode animation) shows a H nucleus motion normal to the Ni(*μ*-H)Fe plane, in what is a unique ‘wagging’ mode. While the Ni–H–Fe wag is relatively isolated in [**1′**H]^+^ (H motion accounts for ∼80% of the kinetic energy), the corresponding Ni-D-Fe motion in [**1′**D]^+^ is predicted to be heavily mixed with Fe–CO modes (Supplementary Table 1). The results of such mixing are evident in the intense 440–630 cm^−1^ Fe–CO region (Fig. 2b,c). Thus, the NRVS signatures of the Ni–H–Fe moiety in the enzyme mimic are the weak wag band observed for [**1′**H]^+^ and the change in the amplified features in the Fe–CO region when comparing spectra of [**1′**H]^+^ and [**1′**D]^+^.

### Hydrogenase NRVS results

**Figure 3:**
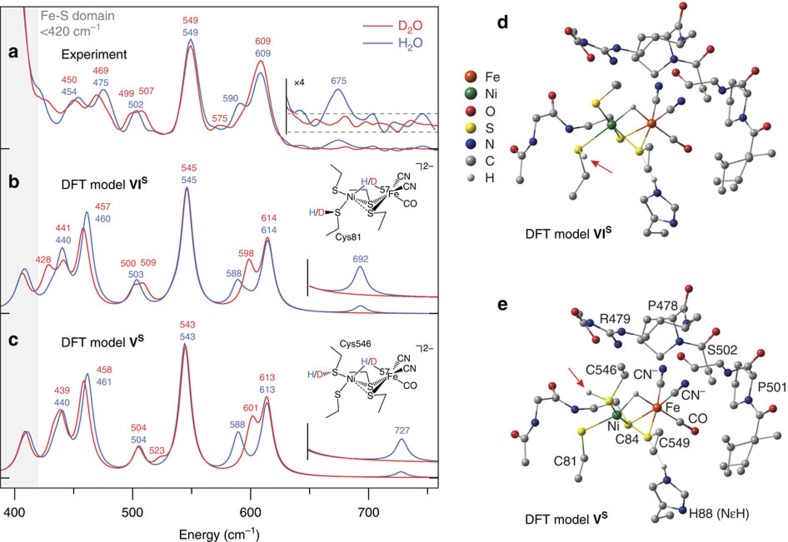
Ni–H–Fe-hydride wag exposed in the reduced state Ni-R of [NiFe]-hydrogenase. (**a**–**c**) High-frequency NRVS for [NiFe]-hydrogenase reduced in H_2_O (blue trace) and D_2_O (red trace; **a**) and the corresponding ^57^Fe PVDOS simulations given for the representative DFT models **VI**^**S**^ (**b**) and **V**^**S**^ (**c**). The higher regions of spectra containing the Ni–H–Fe wag band (in H_2_O samples) are repeated with their intensities × 4 amplified. The low-energy region of the Ni-R spectrum in H_2_O reveals a triplet of bands (454, 475 and 502 cm^−1^) that correspond to those located at 440, 461 and 504 cm^−1^ in the calculated spectrum of the model **V**^**S**^. Further, two intense bands seen at 549 and 609 cm^−1^ in Ni-R map on calculated bands at 543 and 613 cm^−1^, with an additional weak band observed at 590 cm^−1^ that can be correlated with the calculated band appearing at 588 cm^−1^. (**d**,**e**) Representative DFT-optimized models **VI**^**S**^ (**d**) and **V**^**S**^ (**e**) for the Ni-R active site. Arrows indicate the position of CysS*H*. Non-substrate H atoms have been omitted for clarity (excluding *H*N*ε* of His88).

NRVS data for Ni-R in H_2_/H_2_O and in D_2_/D_2_O are compared in Fig. 3a. While vibrations of the three electron transport Fe–S clusters exclusively populate the <420 cm^−1^ region^39^,^40^,^43^, this work instead focuses on the Fe–CO/CN region and higher-energy NRVS features to assign spectroscopic markers characteristic of the bridging hydride. Analysis of Ni-R in H_2_O revealed a sharp band at 549 cm^−1^ previously assigned to a *ν*_Fe–CO_ mode^39^, with additional features at 454, 475 and 502 cm^−1^ arising from a mixture of Fe–CO and Fe–CN modes. Compared with our previous results^39^, the absence of shoulders and additional features around the *ν*_Fe–CO_ band indicates a higher level of sample purity. The NRVS band positions are similar to but nevertheless distinct from Raman peaks for the Ni-L Ni(I)Fe(II) state of [NiFe]-hydrogenase, for which *ν*_Fe–CO_ was observed at 559 cm^−1^ (ref. 53). Samples of Ni-R in H_2_O exhibit bands at 590 and 609 cm^−1^ that collapse to a single peak at 609 cm^−1^ when D_2_O is instead used. Qualitatively, one can attribute the differences in this Fe–CO/CN region to a different coupling to Fe–H and Fe–D motion in the respective samples, as discussed above for [**1′**H/D]^+^. Other details about the active site, such as whether cysteine ligands are unprotonated or protonated, cannot be addressed on the basis of the NRVS data alone.

NRVS analysis of [NiFe]-hydrogenase in H_2_O also revealed a weak but well-resolved band at 675 cm^−1^ not observed for other samples. This band is presumably related to the Ni–H–Fe wag exposed for [**1′**H]^+^ at 758 cm^−1^ (Fig. 2b,d), making this the first assignment of a Fe–H-related mode in any enzyme by NRVS and the first direct spectroscopic evidence for a Ni(*μ*-H)Fe core in Ni-R. Deuteration of Ni-R is expected to red-shift this mode into the 420–620 cm^−1^ Fe–CO/CN region, in agreement with the changes observed and calculated for [**1′**D]^+^. Analogous to the model complex, the Ni-D-Fe wag is strongly mixed with Fe–CO/CN modes, which, in the case of Ni-R, makes its unique assignment very difficult.

### Hydrogenase DFT results

To interpret NRVS measurements in terms of suitable structural candidates for Ni-R, we performed DFT calculations on a series of active site models featuring different binding modes of the H_2_ substrate or its heterolysis products (see Supplementary Fig. 19). Limiting our models to the [NiFe] site is appropriate in that Fe–S clusters do not feature NRVS bands in the >420 cm^−1^ region of interest^39^,^40^. Two main structures were considered: one in which substrate is present in the form of a dihydrogen ligand ((*η*^2^-H_2_)NiFe, **I** or NiFe(*η*^2^-H_2_), **II**) and another where a bridging hydride is present (Ni(*μ*-H)Fe, **III** or HNi(*μ*-H)Fe, **IV**). In addition, variants of **III**, in which a terminal Cys ligand is protonated ((Cys546)SHNi(*μ*-H)Fe, **V** and (Cys81)SHNi(*μ*-H)Fe, **VI**), were also studied. Taking into account the Ni(II)Fe(II) Ni-R active site^16^,^18^, and assuming Fe(II) remains low-spin, each model may exist in electronic singlet (*S*=0) or triplet (*S*=1) Ni(II) states, both of which were evaluated computationally. The DFT-calculated ^57^Fe PVDOS for selected models were compared with the NRVS data for Ni-R over the range 400–750 cm^−1^ (Fig. 3a–c, see ‘Further Discussion of Enzyme Cluster Models’ of the Supplementary Discussion and Supplementary Figs 20–30). DFT-calculated NRVS for models **V**^**S**^ and **VI**^**S**^ (Fig. 3d,e, superscript S denotes singlet Ni(II)) match experimental data remarkably well, with the number and positions of absorption bands being in accordance. Relative intensities of the calculated peaks are also in good agreement with our measurements.

According to the normal mode analysis of model **V**^**S**^ (Supplementary Figs 28 and 29; animated representations of vibrational modes for models **V**^**S**^ and **VI**^**S**^ are provided in Supplementary Movies 4,5,6,7,8,9,10,11,12,13,14,15,16,17,18,19,20,21,22,23,24,25,26,27,28,29,30,31,32,33,34), vibrations in the 440–504 cm^−1^ region predominantly involve Fe–CN bending and stretching, while higher-energy bands (543–613 cm^−1^) are derived from Fe–CO vibrations. The feature calculated at 543 cm^−1^ has significant *ν*_Fe–CO_ character, while that at 613 cm^−1^ is assigned to a *δ*_Fe–CO_ mode. Likewise, the 588 cm^−1^ band can be assigned to an H–Fe–CO bend in which H, Fe and C remain nearly collinear. The above normal modes are highly mixed, which make the assignment of individual fragments complicated.

Both bridging hydride models **V**^**S**^ and **VI**^**S**^ are predicted to exhibit a weak Ni–H–Fe out-of-plane wagging band (at 727 and 692 cm^−1^, respectively, see Fig. 3; Supplementary Movies 11 and 28 for mode animations) whose intensity is comparable to that of the 675 cm^−1^ feature observed for Ni-R. When compared with the Fe–CO/CN region, both theory and experiment predict lower NRVS intensity of the wag in Ni-R than that observed and calculated for [**1′**H]^+^ (at 758 and 774/767 cm^−1^, respectively, see Fig. 2). Moreover, simulations for **V**^**S**^ and **VI**^**S**^ accurately reproduce the disappearance of the 675 cm^−1^ band in data for Ni-R in D_2_O. The difference between the experimental and calculated frequencies of the wagging mode are likely due to limitations in our model, which does not take into account direct contacts between the protonated cysteines and surrounding residues, as well as anharmonicity effects. Moreover, one would expect an intrinsic error of the chosen functional/basis set combination. While hydride bands are extremely sensitive to (electronic) structure, we note that the observed error is still well within normal limits^54^,^55^,^56^,^57^,^58^ expected for this methodology. However, since the full spectral information is considered in the interpretation, the present conclusions can be made with confidence. Finally, calculated H/D isotope shifts for the two representative models (Supplementary Tables 4 and 5) in the low-energy region are also fully consistent with the observed data. The overall analysis here identifies the 675 cm^−1^ feature in the Ni-R NRVS spectrum as the Ni–H–Fe wag mode.

## Discussion

Our NRVS measurements on synthetic bridging hydrides and Ni-R, combined with DFT calculations, provide new constraints on the structure of this key catalytic state of [NiFe]-hydrogenase. Spectra of [**1′**H/D]^+^ feature characteristic Fe–H/D stretches whose energies (1,532/1,468 cm^−1^ for the Ni/Fe-flippamer, respectively) are comparable to those for bridging hydrides in other structures, including another recently reported Ni(*μ*-thiolate)_2_(*μ*-H)Fe species for which infrared spectroscopy revealed a *ν*_Fe–H_ band at 1,687 cm^−1^ (ref. 28). Symmetrical *μ*_2_-H^−^ bridges, such as those in Fe_4_H_4_^+^ clusters, give rise to symmetric stretches at around 1,400 cm^−1^ (ref. 59), while purely terminal hydrides have *ν*_Fe–H_∼1,700–2,300 cm^−1^ (refs 33, 60). The sensitivity of hydride vibrations to structural perturbations underscores their enormous diagnostic value in understanding catalyst structure and function.

Unfortunately, even the strongest of these relatively pure stretches, *ν*_Fe-D_, is predicted to have much lower NRVS intensity than the *ν*_Fe–CO_ modes. Thus, while [**1′**H/D]^+^ allowed for direct observation of *ν*_Fe–H/D_ and *ν*_Ni–H/D_ modes, the resolution of similar bands for [NiFe]-hydrogenase is beyond our current capabilities.

Of special significance is the DFT prediction of a Ni–H–Fe wag (Fig. 2d), this vibration being assigned to an observed NRVS band at 758 cm^−1^ for [**1′**H]^+^, a mode likely obscured by solvent bands in Raman spectra^46^. A key advantage of NRVS is thus demonstrated in that its sole detection of modes coupled to the Mössbauer-active ^57^Fe nucleus makes it unaffected by solvent or matrix modes. The NRVS intensity of the Ni–H–Fe wag is at least four times greater than those of the Ni–H/Fe–H stretches. The Ni–H–Fe wag is a valuable diagnostic probe of the Ni-R structure, with NRVS data for Ni-R in H_2_O featuring a weak but reproducible band at 675 cm^−1^ that is absent when a D_2_O medium was used (Fig. 3). Given that ^57^Fe NRVS-active modes necessarily involve motion of this metal centre, observation of an H/D isotopically sensitive band at 675 cm^−1^ is strong evidence for the presence of a Fe–H moiety in Ni-R.

A second observable indicating the presence of a bridging H^−^/D^−^ in both Ni-R and its mimics [**1′**H/D]^+^ stems from the coupling of Fe–CO and Fe–CN stretches and bends with the Ni-D-Fe wag. While coupling to the Fe–CO/CN modes makes resolution of the Ni-D-Fe wag impossible, it also results in marked changes in band position and intensity in the 440–630 cm^−1^ region on H/D substitution (Figs 2 and 3). In line with the marked isotope effects described above for [**1′**H/D]^+^, NRVS spectra of Ni-R display similar shifts in position and intensity around 450–480 cm^−1^, splitting of a band at 502 cm^−1^, and disappearance of a shoulder peak at 590 cm^−1^. This coupling provides an indirect but powerful method for characterizing theFe–H/D-binding geometry. The NRVS experiments were complemented by DFT simulations, which further pointed to the presence of an active-site bridging hydride. Spectra predicted for the singlet (*S*=0) models (Cys81)SHNi(*μ*-H)Fe (**VI**^**S**^) and (Cys546)SHNi(*μ*-H)Fe (**V**^**S**^) reproduce the data exceptionally well, in particular with respect to the Ni–H–Fe wag in each H/D isotopologue. The model **V**^**S**^ is also supported by the recent high-resolution crystallographic analysis of Ni-R1 (ref. 27). As detailed in the ‘Further Discussion of Enzyme Cluster Models’ section of the Supplementary Discussion, the NRVS data are best reproduced assuming a low-spin Ni(II). DFT also indicated asymmetric binding of H^−^ to the Ni(II)Fe(II) core, with the ligand more strongly bound to Ni than to Fe (see ‘Electronic Structure of Model V’ of the Supplementary Discussion).

Taken together, NRVS analysis of Ni-R, in combination with NRVS and DFT data for [**1′**H/D]^+^, indicates that models **VI**^**S**^ and **V**^**S**^ provide a consistent and detailed picture of Ni-R. This cohesive study thus represents the first evidence from vibrational spectroscopy for the presence of a bridging H^−^ in the active site of Ni-R, a state central to the function of [NiFe]-hydrogenase. The combined experimental and theoretical approach described here has applicability far beyond the Ni-R and [NiFe]-hydrogenase. Ideal for the detailed study of Fe–H fragments, we envisage that such methods will also be of importance for unravelling the mechanisms of [FeFe]-hydrogenase and nitrogenase^61^, as well as for the development of synthetic catalysts inspired by these metalloenzymes.

## Methods

### General

Protocols employed for chemical and biochemical synthesis, as well as NRVS measurements and DFT calculations, are outlined here. Full procedures, including associated references, are given in the Supplementary Methods.

### Model complex preparation

Metallic ^57^Fe was converted to the complex (dppe)Ni(*μ*-pdt)^57^Fe(CO)_3_ (**1′**) via the intermediates ^57^Fe_2_I_4_(2-propanol)_4_, (dppe)Ni(*μ*-pdt)^57^FeI_2_ and [(dppe)Ni(*μ*-pdt)(*μ*-I)^57^Fe(CO)_3_]BF_4_ ([**1′**I]BF_4_) (ref. 52). Species **1′** underwent facile exchange with ^13^CO (1 atm), allowing access to isotopologue (dppe)Ni(*μ*-pdt)^57^Fe(^13^CO)_3_ (**1″**). The two Ni(I)Fe(I) derivatives **1′** and **1″**were then subjected to protonation and deuteronation (effected with excess HBF_4_ and HBF_4_/CD_3_OD, respectively), affording the Ni(II)Fe(II) salts [**1′**H]BF_4_, [**1′**D]BF_4_, [**1′**H]BF_4_ and [**1″**D]BF_4_ as crystalline solids. The isotopic purity of the hydrides and deuterides was confirmed using multinuclear (^1^H, ^2^H, ^13^C and ^31^P) NMR spectroscopy, infrared spectroscopy and ESI mass spectrometry. NRVS analysis was conducted on a solid sample of each of the four model complexes (*vide infra*).

### *D. vulgaris* Miyazaki F [NiFe]-hydrogenase preparation

[NiFe]-hydrogenase expressed in *D. vulgaris* was isolated and purified as described earlier^62^. The as-isolated protein was transferred from 25 mM Tris-HCl (pH=7.4) buffer to 100 mM MES (pH=5.0). The solution was placed in a tube, which was sealed, degassed and then purged with H_2_ (1.2 bar) for 8 h. In the case of the sample in D_2_O, the buffer was replaced by 100 mM MES (pD=5.0) in D_2_O and the mixture placed under D_2_ (1.3 bar) for 8 h. Samples were transferred to an anaerobic chamber and loaded into NRVS cells. FT-IR spectra were recorded on a Bruker IFS66v/S FT-IR spectrometer with a 2 cm^−1^ spectral resolution at 293 K (Supplementary Fig. 31).

### NRVS measurements and data analysis

The NRVS data were collected according to a published procedure^39^ at SPring-8 BL09XU (with flux ∼1.4 × 10^9^ photons s^−1^) and BL19LXU (∼6 × 10^9^ photons s^−1^) using 14.4 keV radiation at 0.8 meV resolution. The spectral maximum counts/second (cts s^−1^) at BL19 is ∼2.6–3 times of that at BL09. To compare the data from different beamlines, we rescale the BL19 counting time on the basis of its max cts s^−1^ versus the max cts s^−1^ at BL09 and create BL09 equivalent seconds, for example, the 10 (s) per point (s/pt) at BL19 is corresponding to 26 or 30 equivalent s/pt at BL09. Delayed nuclear and Fe K fluorescence (from internal conversion) were recorded with a 2 × 2 APD (avalanche photodiode) array in either beamline, and raw NRVS data were converted to single-phonon ^57^Fe PVDOS using the PHOENIX software^39^. Sample temperatures were maintained at 30–50 K during analysis.

The average cts s^−1^ is 0.6–0.8 at the Fe–CO peak (609 cm^−1^) and 0.10–0.12 at X–Fe–H peak (675 cm^−1^), while the measured dark background cts are ∼0.03 cts s^−1^. To improve weak features, we use 1–3 s/pt from −240 to 400 cm^−1^, 5–10 s/pt for the Fe–CN and Fe–CO regions, and 10–30 s/pt for the candidate X–Fe–H bending region (at 620–770 cm^−1^).

### Model complex calculations

Initial coordinates for the DFT calculations on [**1′**H/D]^+^ were extracted from the X-ray structure of [**1**H]BF_4_·3THF (ref. 44). The methodology applied was mostly equivalent to our earlier set-up on [**1′**]^0/+^ (ref. 52). Structural optimizations and subsequent normal mode analyses were performed using GAUSSIAN 09 on the basis of the densities exported from single point calculations using JAGUAR 7.9. The BP86 functional and the LACV3P** basis set were employed. The environment was considered using a self-consistent reaction field model. ^57^Fe PVDOS spectra were generated using Q-SPECTOR, successfully applied earlier^33^. Simulated spectra were broadened by convolution with a full-width at half-maximum=12 cm^−1^ Lorentzian.

### Enzyme cluster model calculations

DFT calculations on active-site cluster models were performed using ORCA 3.0. The initial geometry was prepared from the crystal structure of reduced [NiFe]-hydrogenase from *Desulfovibrio vulgaris* Miyazaki F (PDB 1H2R)^63^. Constraint geometry optimizations and vibrational frequency calculations at the scalar relativistic level (ZORA) employed B3LYP(-D3) hybrid-GGA with RIJCOSx and the COSMO model (*ε*=4). Segmented all-electron relativistically contracted basis sets with corresponding auxiliary basis sets were used (def2-TZVPP: Ni, Fe, CN^−^, CO, Sγ, H^−^, H^+^, H_2_.; def2-SV(P): remaining atoms). Vibrational frequencies and normal mode compositions were utilized to simulate NRVS data (Lorentzian fitting, linewidth 12 cm^−1^).

## Additional information

**How to cite this article:** Ogata, H. *et al*. Hydride bridge in [NiFe]-hydrogenase observed by nuclear resonance vibrational spectroscopy. *Nat. Commun.* 6:7890 doi: 10.1038/ncomms8890 (2015).

## Supplementary Material

Supplementary Information Supplementary Figures 1-31, Supplementary Tables 1-4, Supplementary Note 1, Supplementary Discussion, Supplementary Methods and Supplementary References

Supplementary Movie 1 Model complex [1'H]+ (H isotopomer), Fe-H stretching mode, v = 1479 cm-1

Supplementary Movie 2 Model complex [1'H]+ (H isotopomer), Ni-H stretching mode, v = 1022 cm-1

Supplementary Movie 3 Model complex [1'H]+ (H isotopomer), Ni-H-Fe wagging mode, v = 774 cm-1

Supplementary Movie 4 Enzyme cluster model V (H isotopomer), Singlet state, v = 411.00 cm-1

Supplementary Movie 5 Enzyme cluster model V (H isotopomer), Singlet state, v = 439.93 cm-1

Supplementary Movie 6 Enzyme cluster model V (H isotopomer), Singlet state, v = 460.91 cm-1

Supplementary Movie 7 Enzyme cluster model V (H isotopomer), Singlet state, v = 504.68 cm-1

Supplementary Movie 8 Enzyme cluster model V (H isotopomer), Singlet state, v = 543.53 cm-1

Supplementary Movie 9 Enzyme cluster model V (H isotopomer), Singlet state, v = 588.69 cm-1

Supplementary Movie 10 Enzyme cluster model V (H isotopomer), Singlet state, v = 613.20 cm-1

Supplementary Movie 11 Enzyme cluster model V (H isotopomer), Singlet state, v = 726.92 cm-1

Supplementary Movie 12 Enzyme cluster model V (D isotopomer), Singlet state, v = 408.20 cm-1

Supplementary Movie 13 Enzyme cluster model V (D isotopomer), Singlet state, v = 432.27 cm-1

Supplementary Movie 14 Enzyme cluster model V (D isotopomer), Singlet state, v = 438.72 cm-1

Supplementary Movie 15 Enzyme cluster model V (D isotopomer), Singlet state, v = 458.01 cm-1

Supplementary Movie 16 Enzyme cluster model V (D isotopomer), Singlet state, v = 504.14 cm-1

Supplementary Movie 17 Enzyme cluster model V (D isotopomer), Singlet state, v = 522.60 cm-1

Supplementary Movie 18 Enzyme cluster model V (D isotopomer), Singlet state, v = 544.16 cm-1

Supplementary Movie 19 Enzyme cluster model V (D isotopomer), Singlet state, v = 600.64 cm-1

Supplementary Movie 20 Enzyme cluster model V (D isotopomer), Singlet state, v = 613.26 cm-1

Supplementary Movie 21 Enzyme cluster model VI (H isotopomer), Singlet state, v = 409.49 cm-1

Supplementary Movie 22 Enzyme cluster model VI (H isotopomer), Singlet state, v = 439.68 cm-1

Supplementary Movie 23 Enzyme cluster model VI (H isotopomer), Singlet state, v = 460.61 cm-1

Supplementary Movie 24 Enzyme cluster model VI (H isotopomer), Singlet state, v = 502.78 cm-1

Supplementary Movie 25 Enzyme cluster model VI (H isotopomer), Singlet state, v = 545.14 cm-1

Supplementary Movie 26 Enzyme cluster model VI (H isotopomer), Singlet state, v = 588.10 cm-1

Supplementary Movie 27 Enzyme cluster model VI (H isotopomer), Singlet state, v = 614.86 cm-1

Supplementary Movie 28 Enzyme cluster model VI (H isotopomer), Singlet state, v = 692.53 cm-1

Supplementary Movie 29 Enzyme cluster model VI (D isotopomer), Singlet state, v = 406.20 cm-1

Supplementary Movie 30 Enzyme cluster model VI (D isotopomer), Singlet state, v = 427.43 cm-1

Supplementary Movie 31 Enzyme cluster model VI (D isotopomer), Singlet state, v = 440.82 cm-1

Supplementary Movie 32 Enzyme cluster model VI (D isotopomer), Singlet state, v = 457.11 cm-1

Supplementary Movie 33 Enzyme cluster model VI (D isotopomer), Singlet state, v = 500.43 cm-1

Supplementary Movie 34 Enzyme cluster model VI (D isotopomer), Singlet state, v = 508.69 cm-1

Supplementary Movie 35 Enzyme cluster model VI (D isotopomer), Singlet state, v = 545.50 cm-1

Supplementary Movie 36 Enzyme cluster model VI (D isotopomer), Singlet state, v = 597.65 cm-1

Supplementary Movie 37 Enzyme cluster model VI (D isotopomer), Singlet state, v = 613.77 cm-1

## Figures and Tables

**Figure 1:**
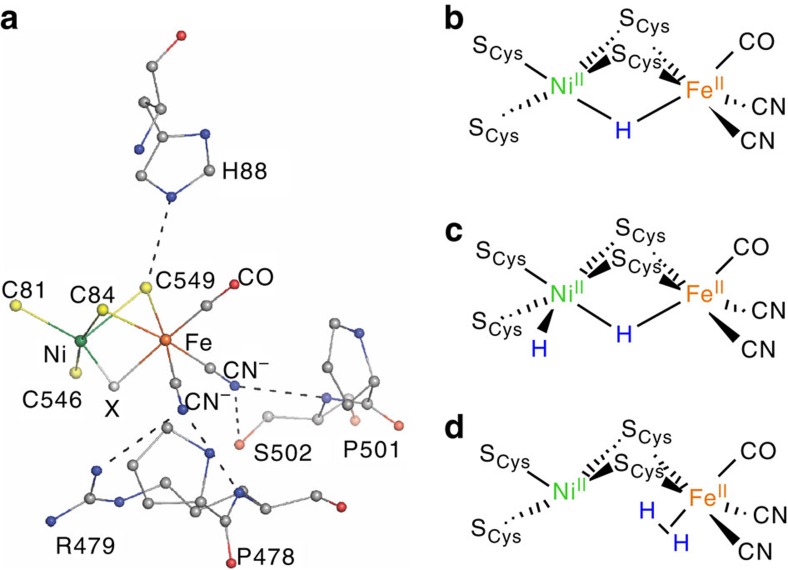
The Ni-R reduced state of [NiFe]-hydrogenase. (**a**) X-ray structure of the *Desulfovibrio vulgaris* Miyazaki F (*Dv*MF) [NiFe]-hydrogenase active site from PDB entry 1WUI (ref. 62). The bridging ligand X is oxygenic for deactivated states, with catalytically active states having either a hydride or a vacant site. (**b**–**d**) Some of the structures proposed for isoelectronic Ni-R forms^19^,^20^,^21^,^22^,^23^,^24^,^25^,^26^.
