# Supplementary material for: Hydride bridge in [NiFe]-hydrogenase observed by nuclear resonance vibrational spectroscopy
Source: Nat Commun. 2015 Aug 10;6:7890. doi: 10.1038/ncomms8890 (PMC4531378; doi:10.1038/ncomms8890)
Supplement: Supplementary Information — Supplementary Figures 1-31, Supplementary Tables 1-4, Supplementary Note 1, Supplementary Discussion, Supplementary Methods and Supplementary References [file ncomms8890-s1.pdf]

Supplementary Figures

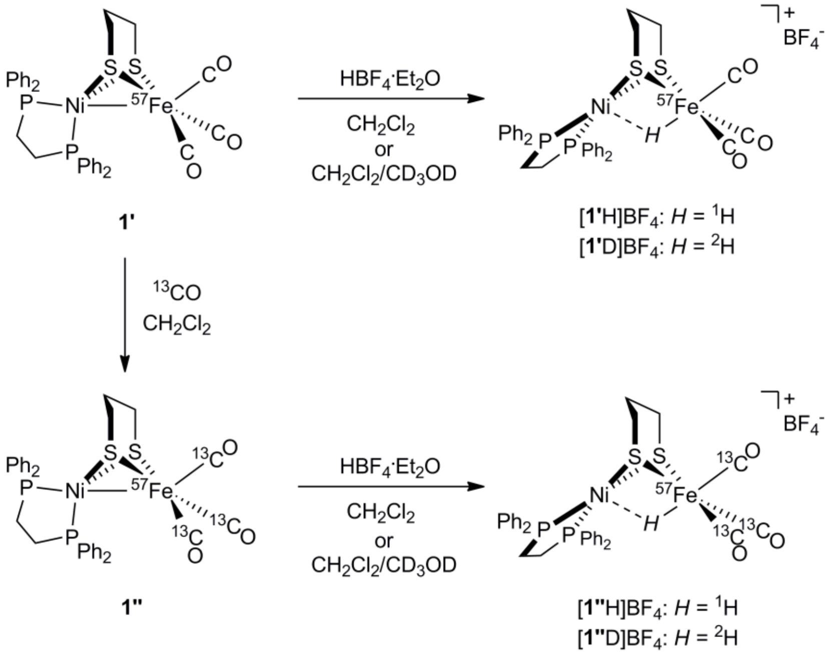

Supplementary Figure 1. Synthesis of labeled $Ni^{57}Fe$ complexes.

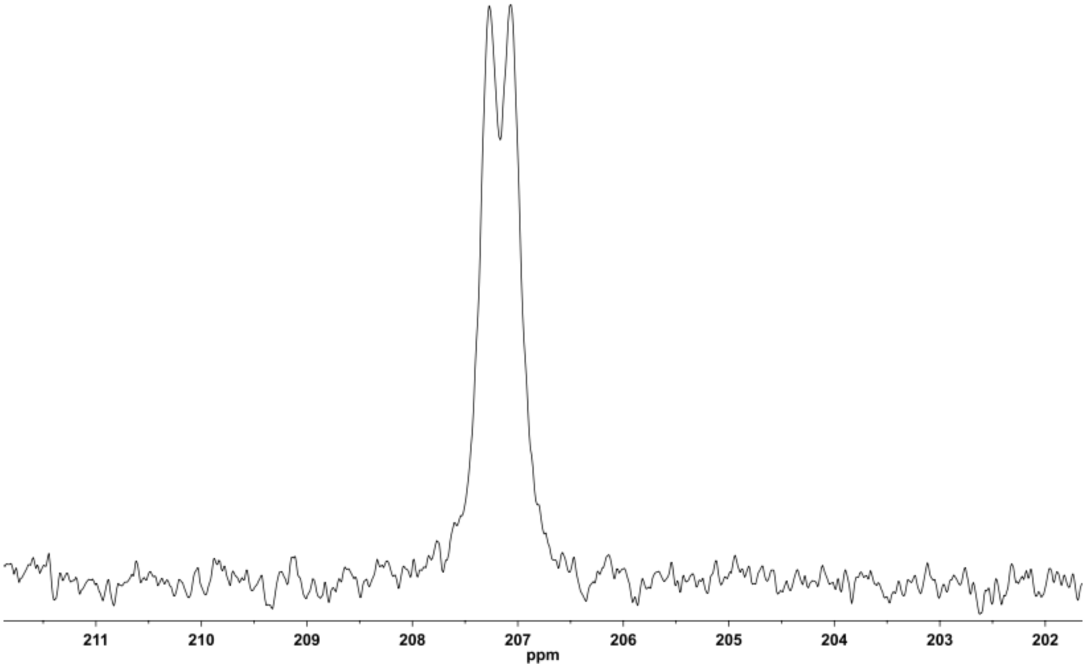

Supplementary Figure 2. $^{13}C\{^1H\}$ NMR spectrum (CH_2Cl_2) of **1″**.

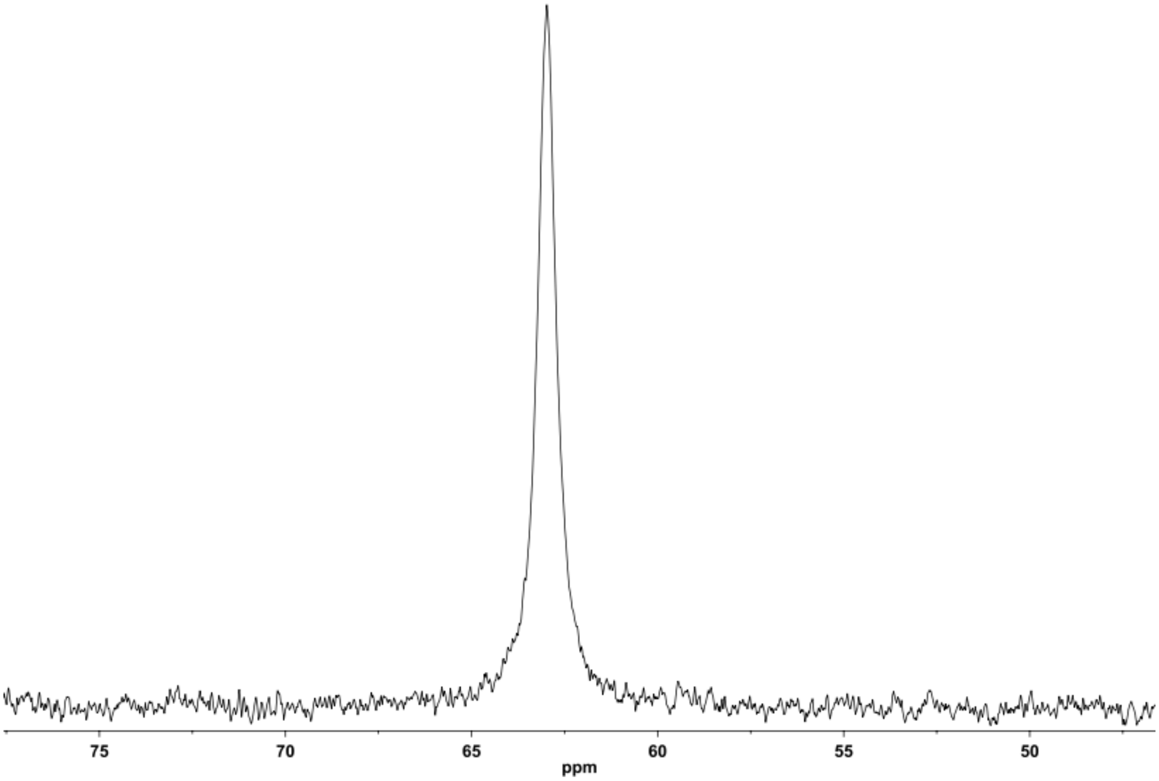

Supplementary Figure 3. $^{31}P\{^1H\}$ NMR spectrum (CH_2Cl_2) of **1″**.

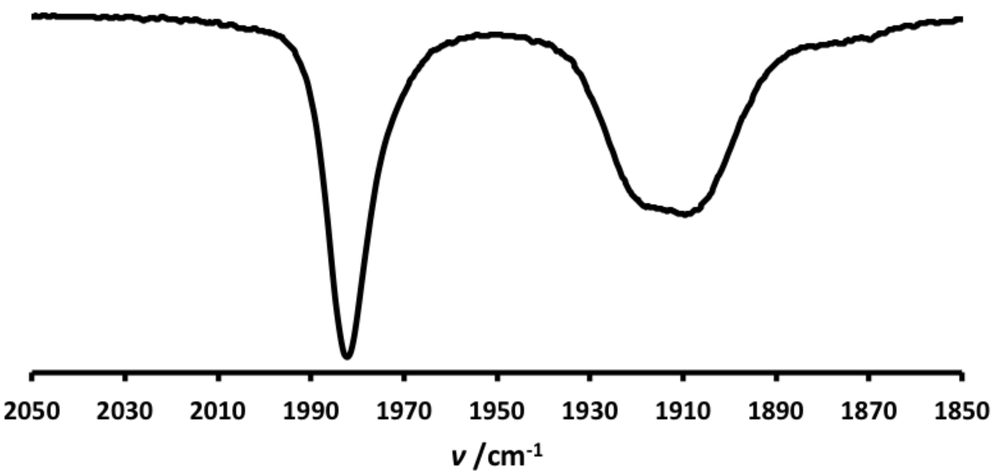

Supplementary Figure 4. FT-IR spectrum (ν_{CO} region, CH_2Cl_2) of **1′′**.

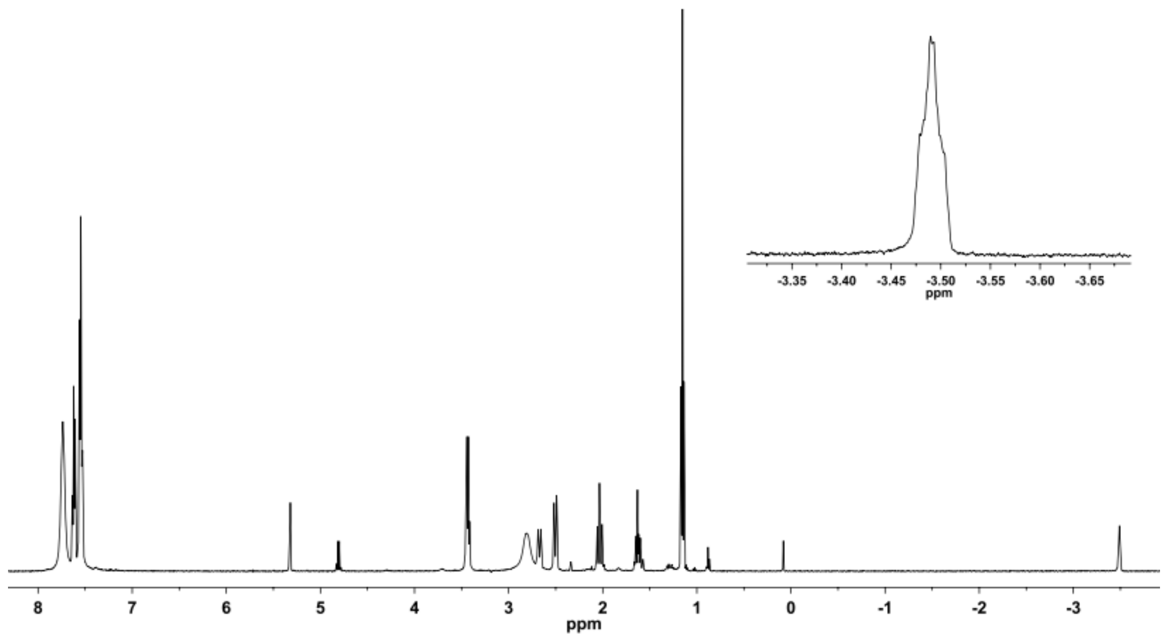

Supplementary Figure 5. ^{1}H NMR spectrum (CD_2Cl_2) of [**1′**H]BF_4. Resonances at 3.43 (Et_2O), 1.31 (pentane), 1.12 (Et_2O) and 0.89 ppm (pentane) are from solvent impurities.

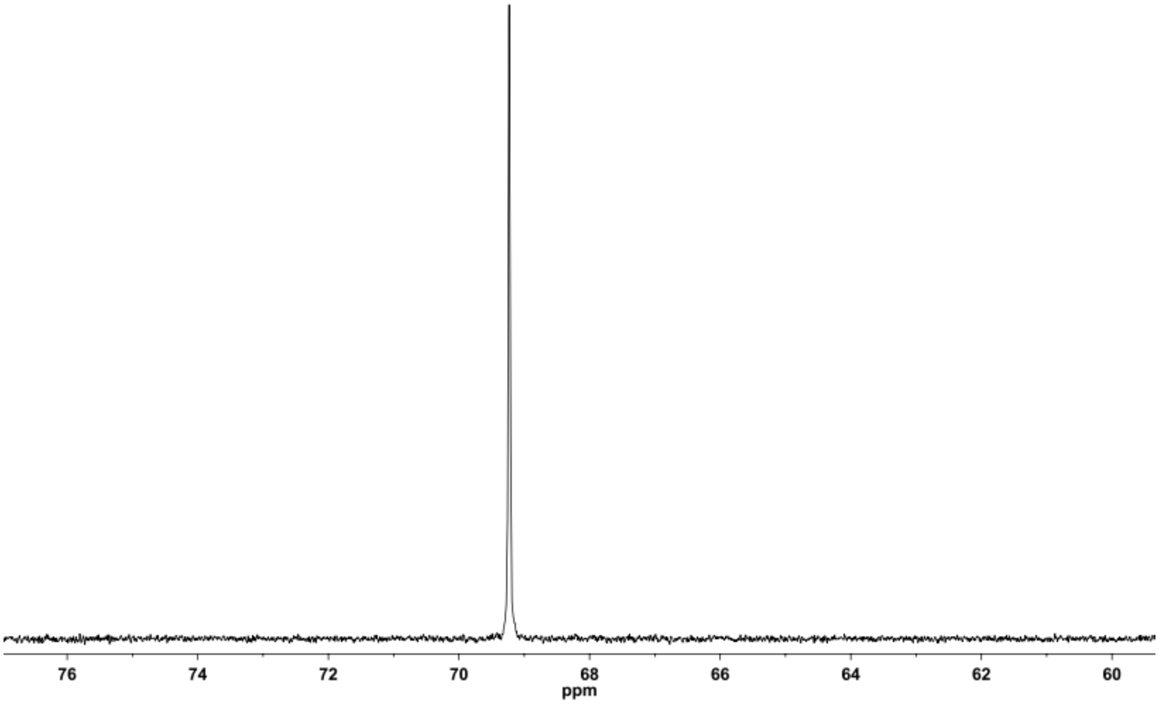

Supplementary Figure 6. $^{31}P\{^{1}H\}$ NMR spectrum (CD_2Cl_2) of [**1′**H]BF_4.

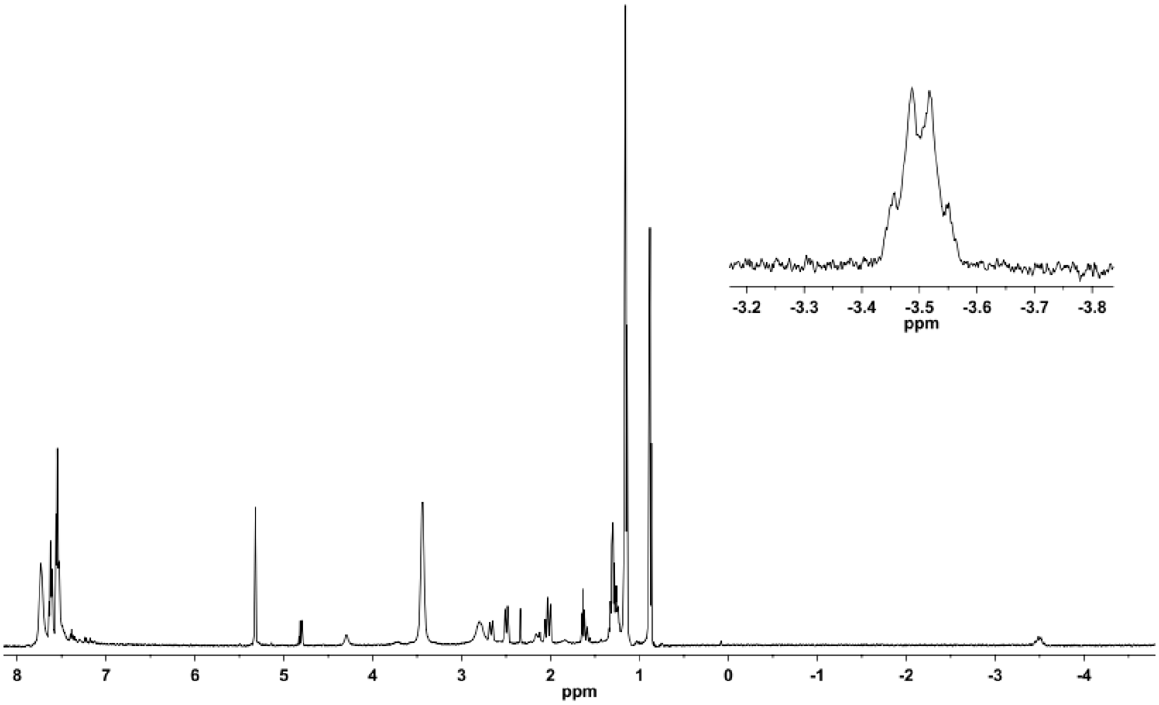

Supplementary Figure 7. ^{1}H NMR spectrum (CD_2Cl_2) of [**1″**H]BF_4. Resonances at 3.43 (Et_2O), 1.31 (pentane), 1.12 (Et_2O) and 0.89 ppm (pentane) are from solvent impurities.

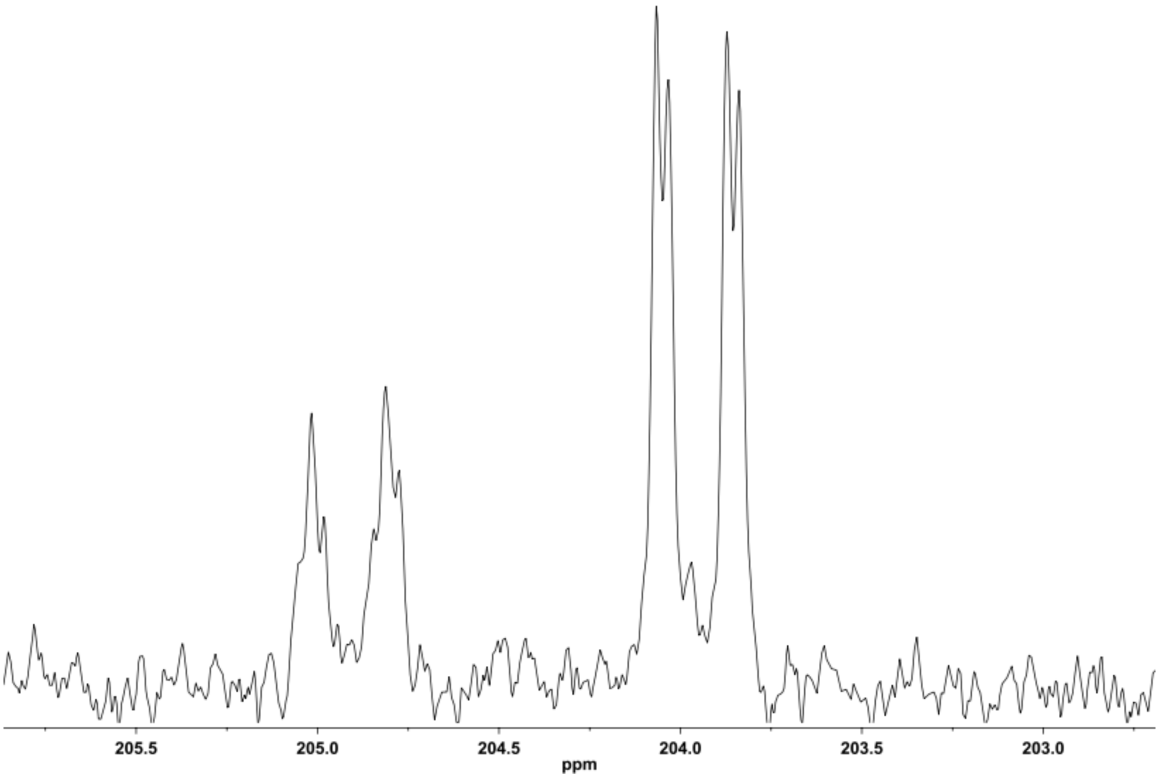

Supplementary Figure 8. $^{13}C\{^1H\}$ NMR spectrum (CD_2Cl_2) of [**1′′**H]BF_4.

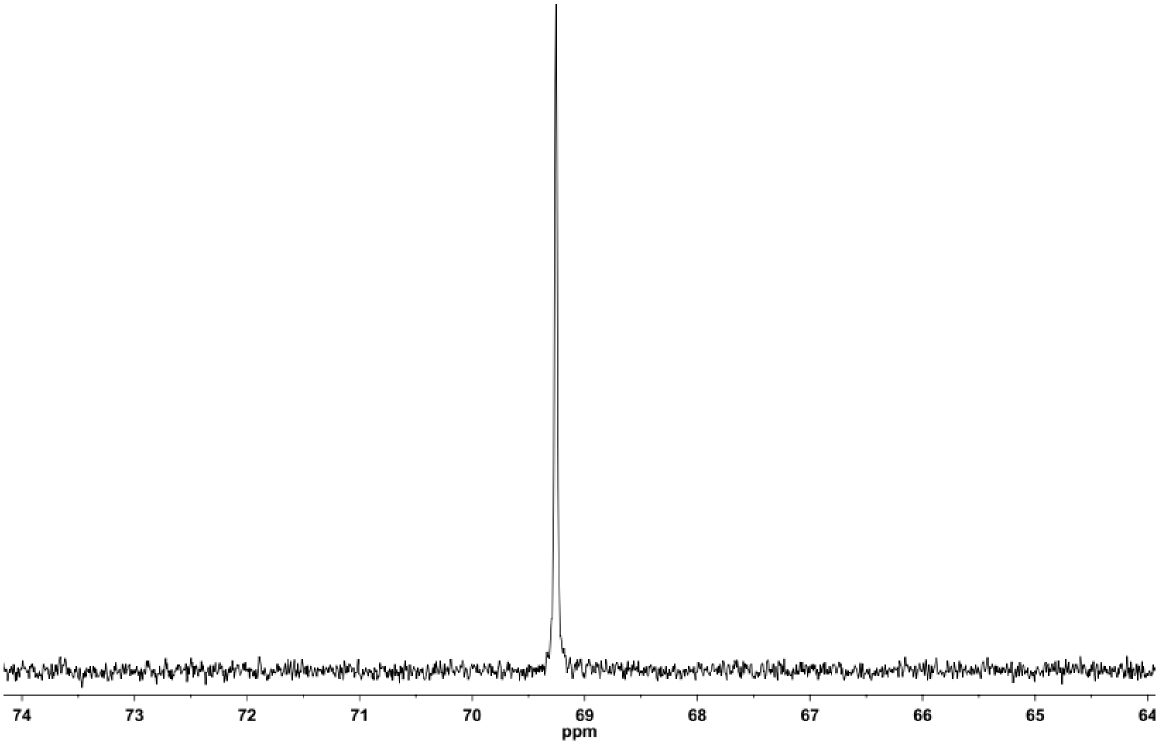

Supplementary Figure 9. $^{31}P\{^1H\}$ NMR spectrum (CD_2Cl_2) of [**1′′**H]BF_4.

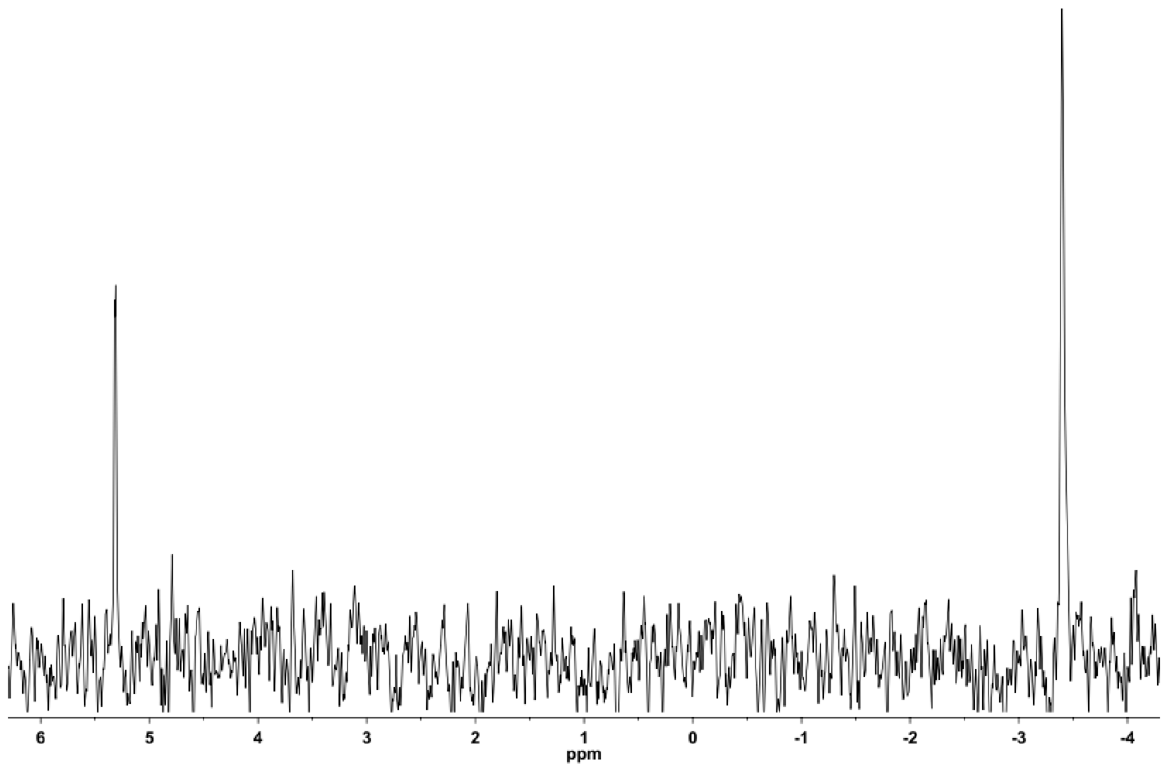

Supplementary Figure 10. ^{2}H NMR spectrum (CH_2Cl_2) of [**1′**D]BF_4. The resonance at 5.32 ppm is from $CDHCl_2$.

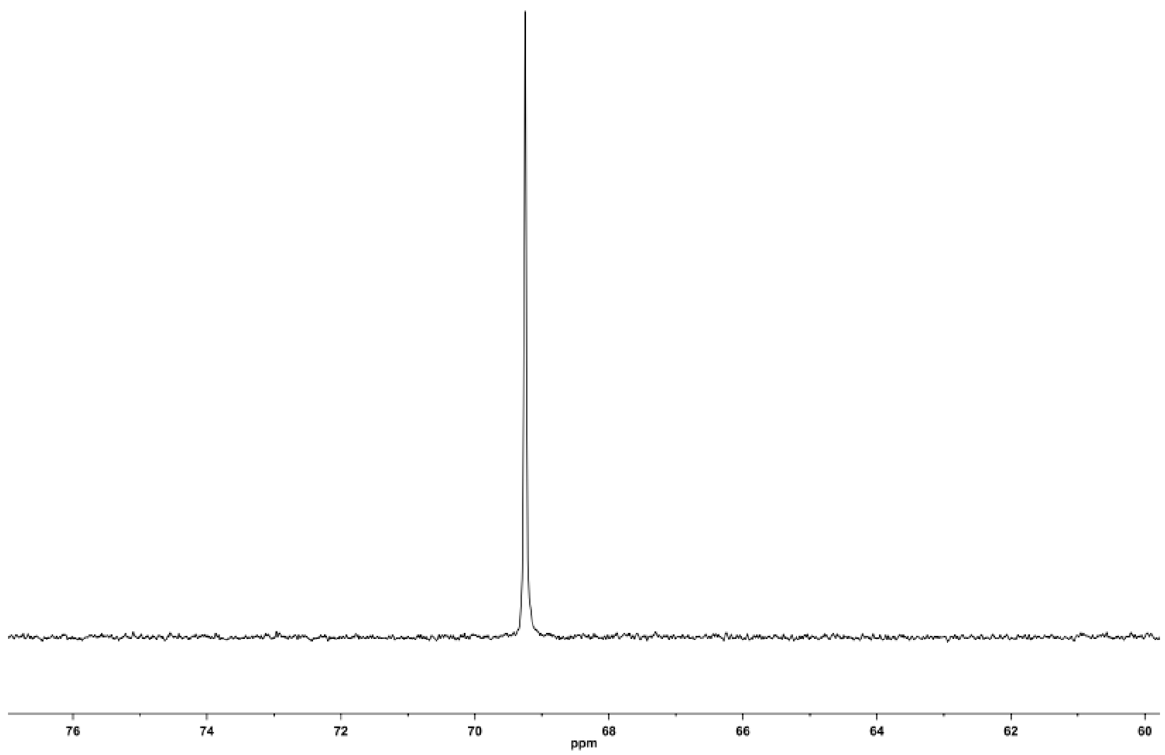

Supplementary Figure 11. $^{31}P\{^{1}H\}$ NMR spectrum (CH_2Cl_2) of [**1′**D]BF_4.

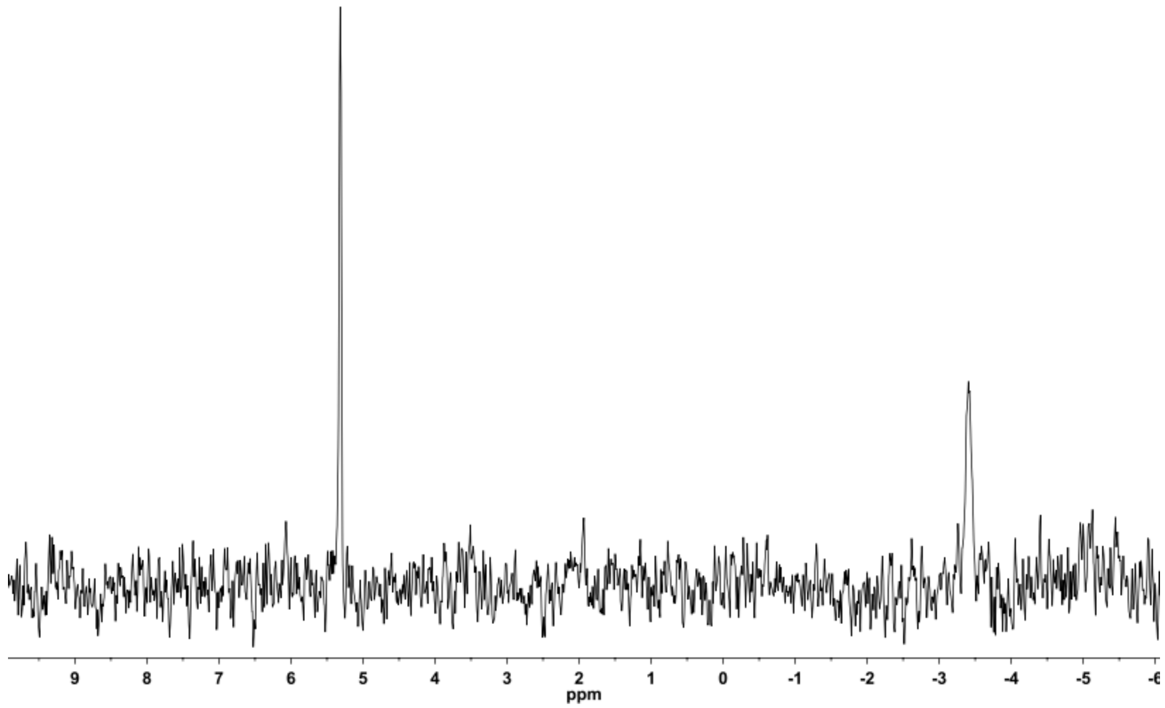

Supplementary Figure 12. ^{2}H NMR spectrum (CH_2Cl_2) of [**1″**D]BF_4. The resonance at 5.32 ppm is from $CDHCl_2$.

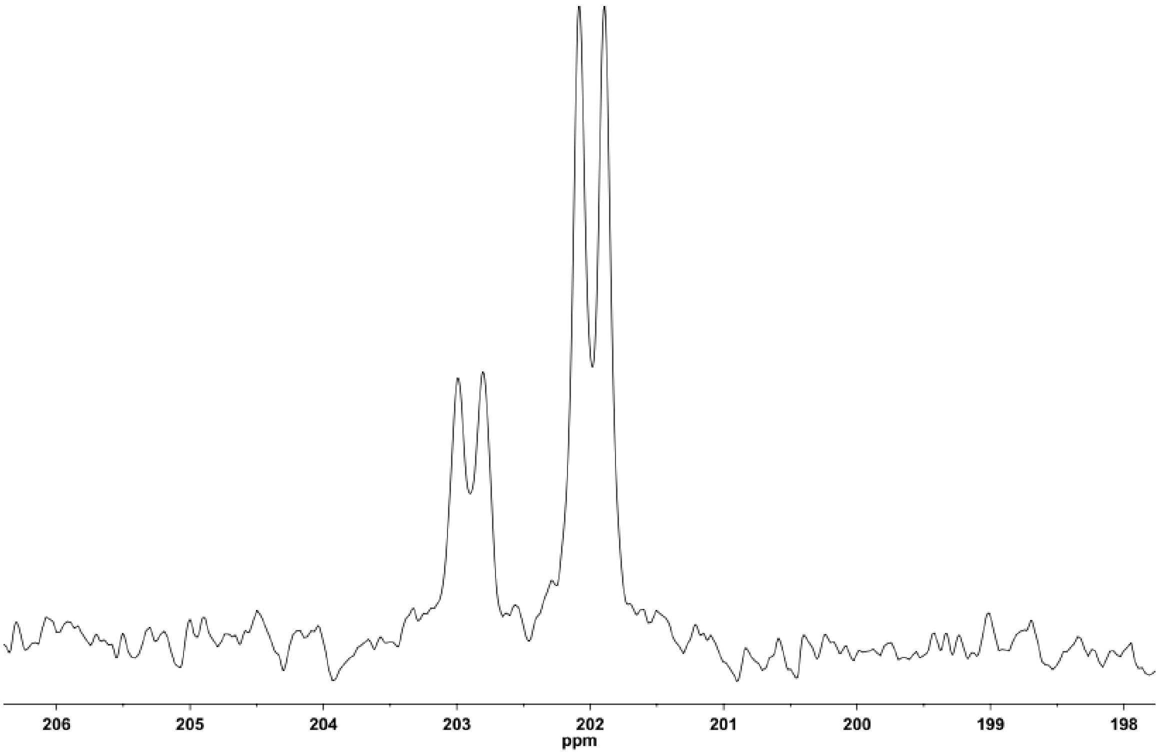

Supplementary Figure 13. $^{13}C\{^1H\}$ NMR spectrum (CD_2Cl_2) of [**1′′**D]BF_4.

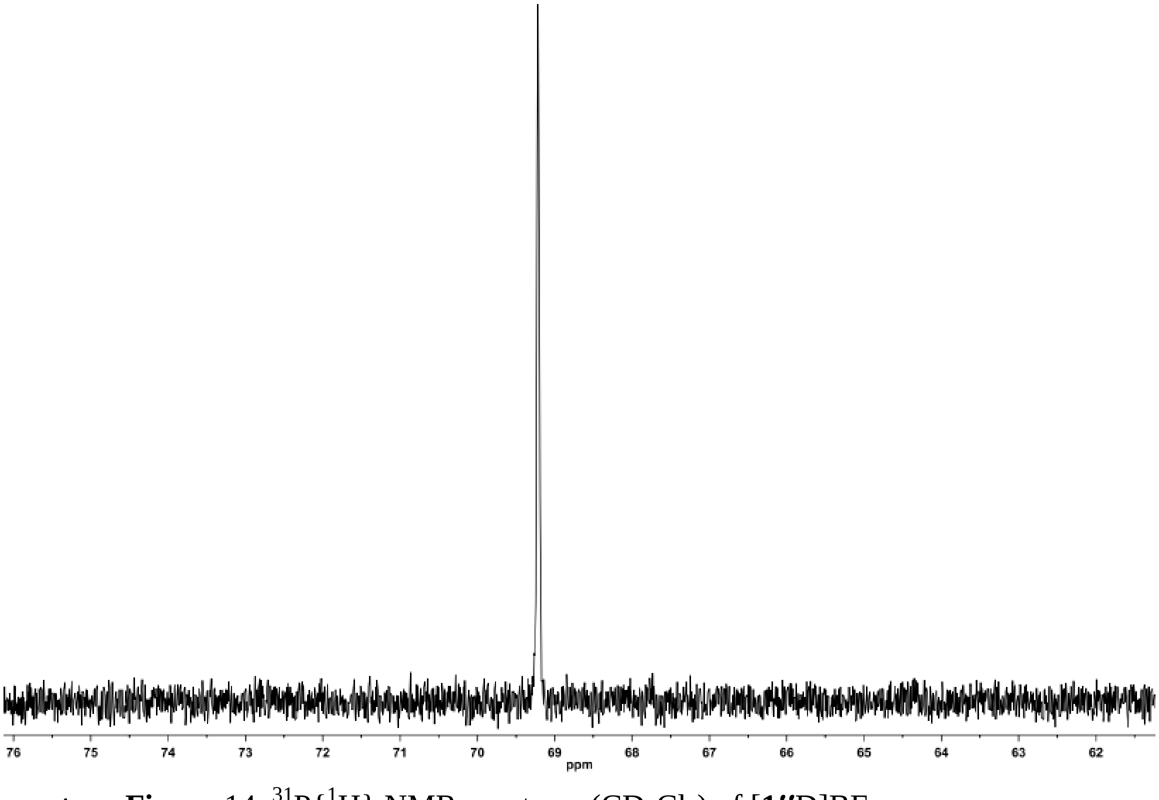

Supplementary Figure 14. $^{31}P\{^1H\}$ NMR spectrum (CD_2Cl_2) of [**1′′**D]BF_4.

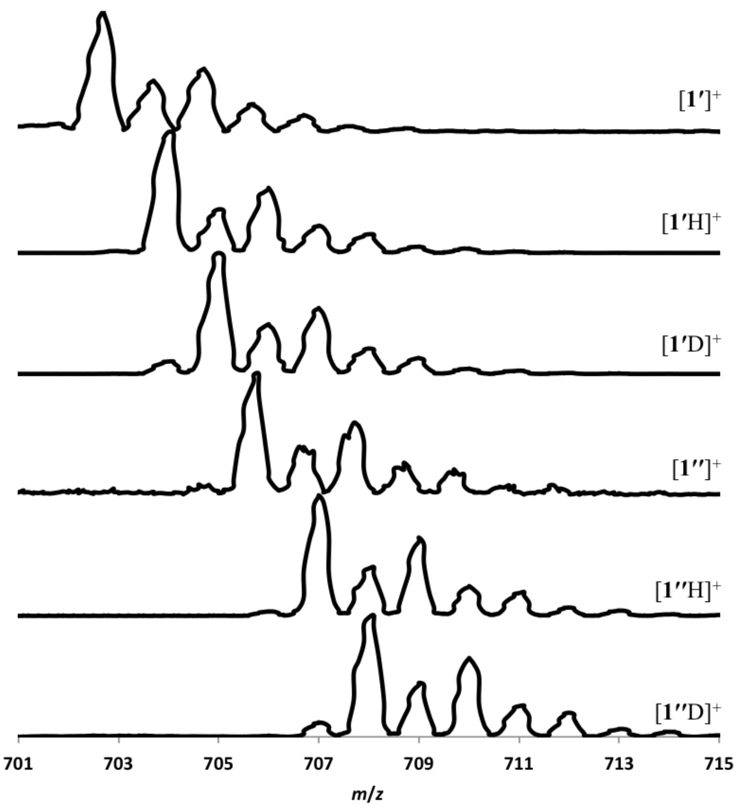

Supplementary Figure 15. Positive ion ESI mass spectra of synthetic model complexes.

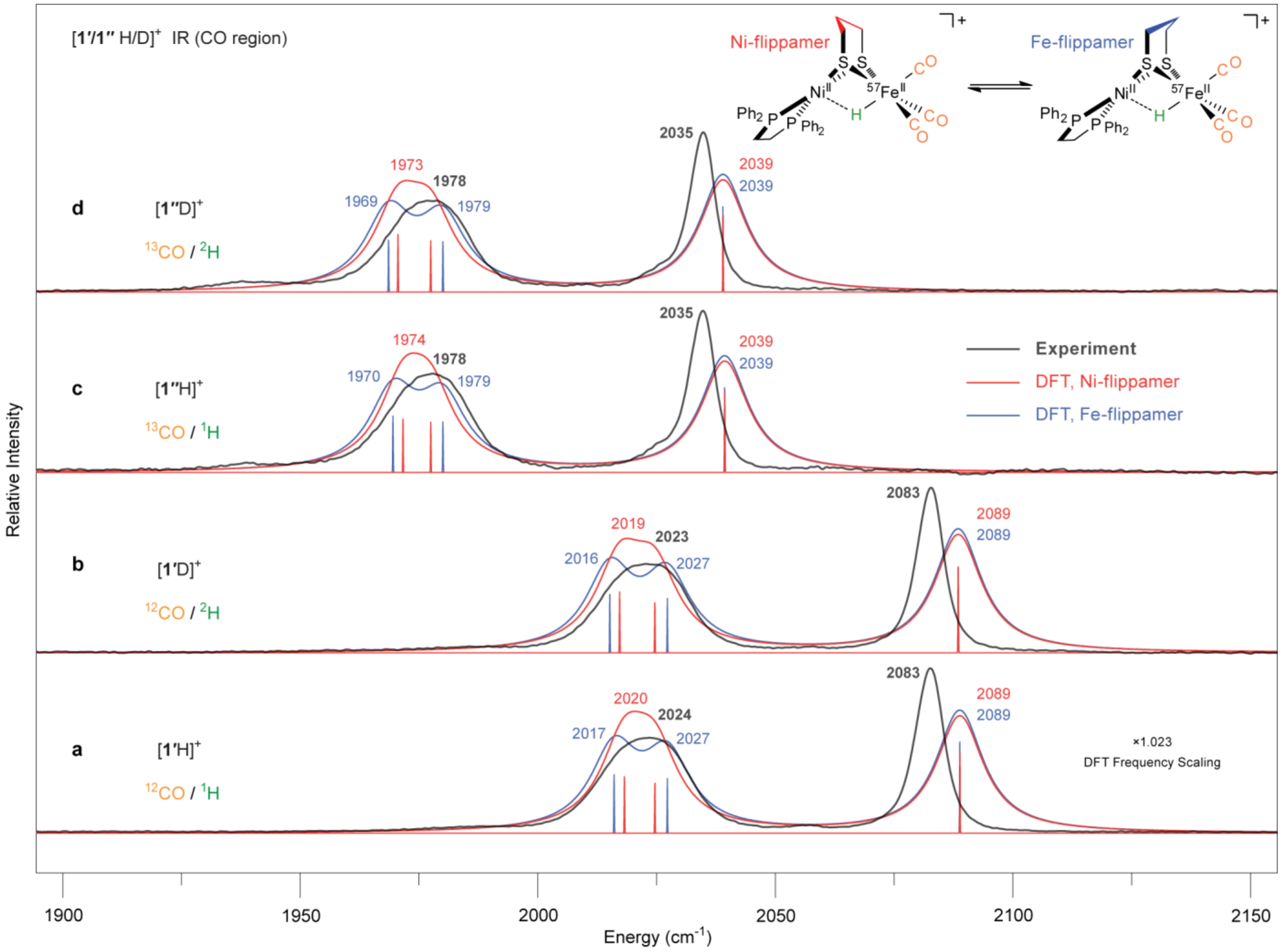

Supplementary Figure 16. FT-IR spectra (ν_{CO} region) of: **a,** [**1′**H]+, **b,** [**1′**D]+, **c,** [**1″**H]+ and **d,** [**1″**D]+. The experimental data (collected in CH_2Cl_2 solution) are presented in black, while the DFT-predicted spectra for Ni- and Fe-flippamers are in red and blue, respectively.

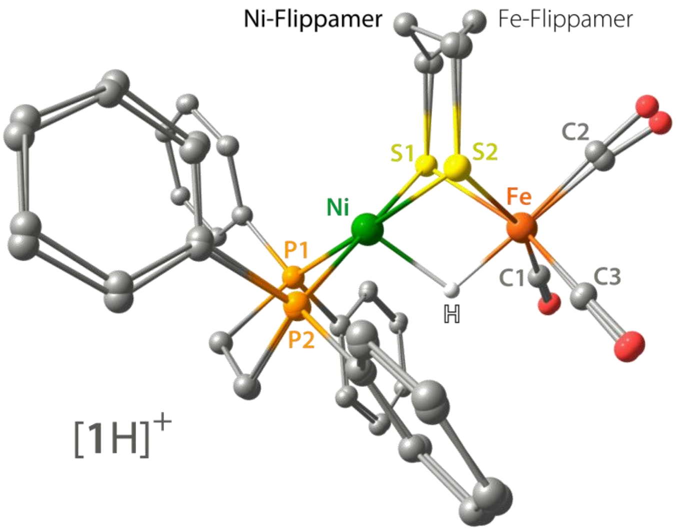

Supplementary Figure 17. Overlay of the DFT-optimized structures for the dominant (Ni-flippamer) and alternative (Fe-flippamer) conformers of [**1**H]$^{+}$. H atoms, apart from the hydride bridge, are omitted for clarity.

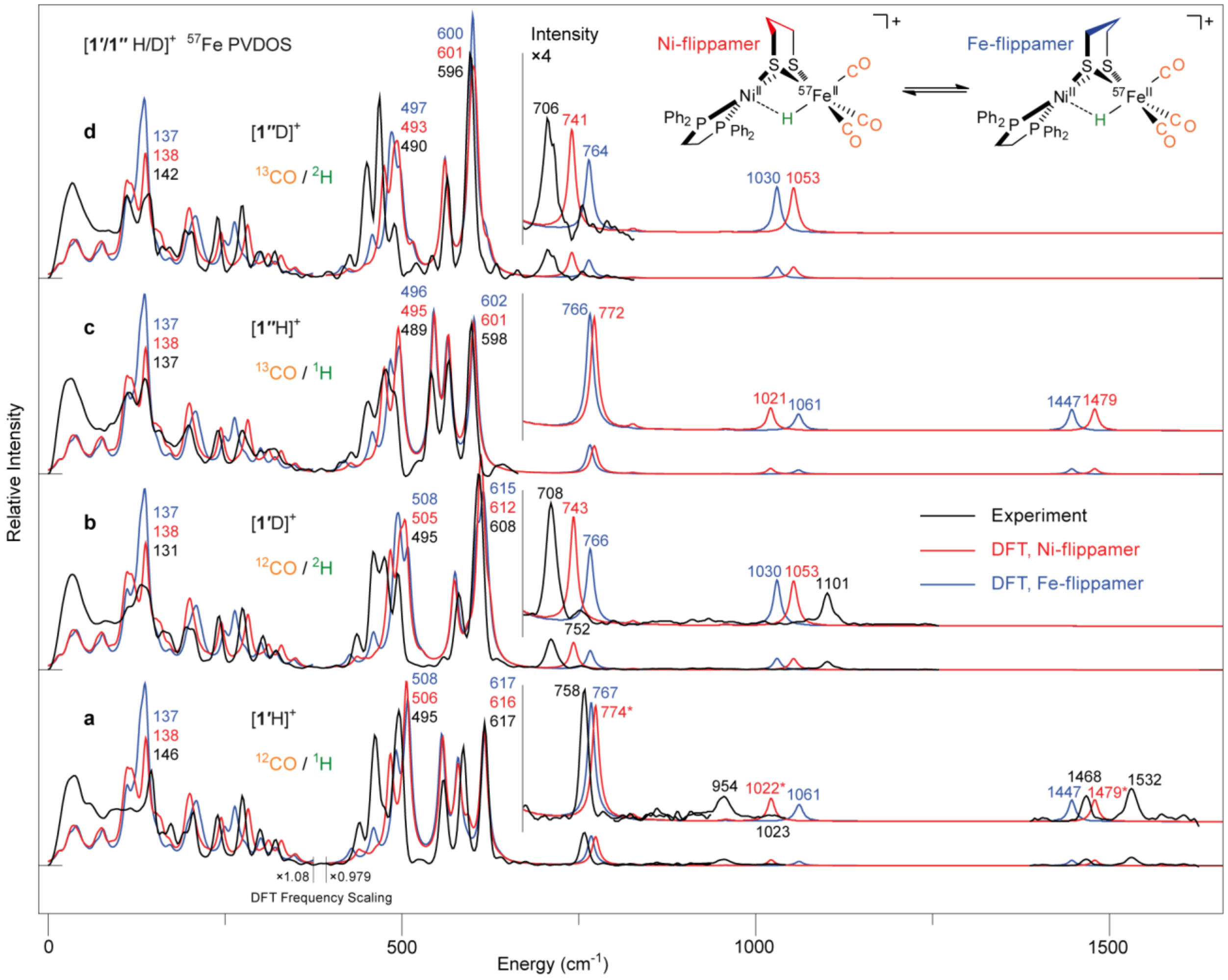

Supplementary Figure 18. ^{57}Fe PVDOS spectra for **a**, [**1′**H]$^{+}$ (^{12}CO/^{1}H), **b**, [**1′**D]$^{+}$ (^{12}CO/^{2}H), **c**, [**1″**H]$^{+}$ (^{13}CO/^{1}H), and **d**, [**1″**D]$^{+}$ (^{13}CO/^{2}H) isotopologues derived from NRVS data (black) and DFT calculations on Ni/Fe-flippamers (red/blue, respectively). Above 700 cm^{-1}, all spectra are repeated with their intensities ×4 amplified. Animated representations of the M–H normal modes indicated (*) in **a** are available as part of the Electronic Supplementary Information.

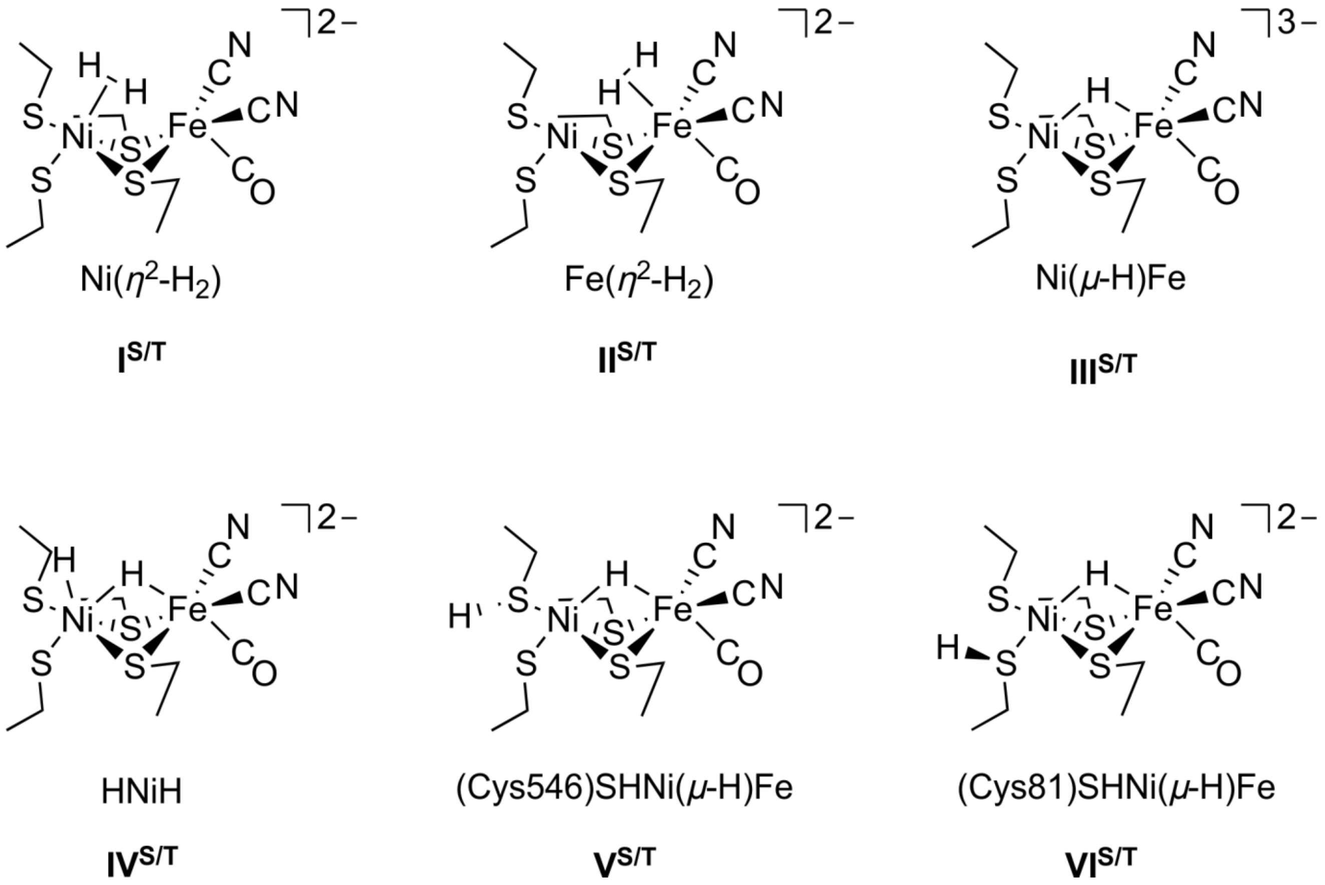

Supplementary Figure 19. Active site models used for DFT-calculated NRVS spectra. Residues in the secondary coordination sphere have been omitted for clarity.

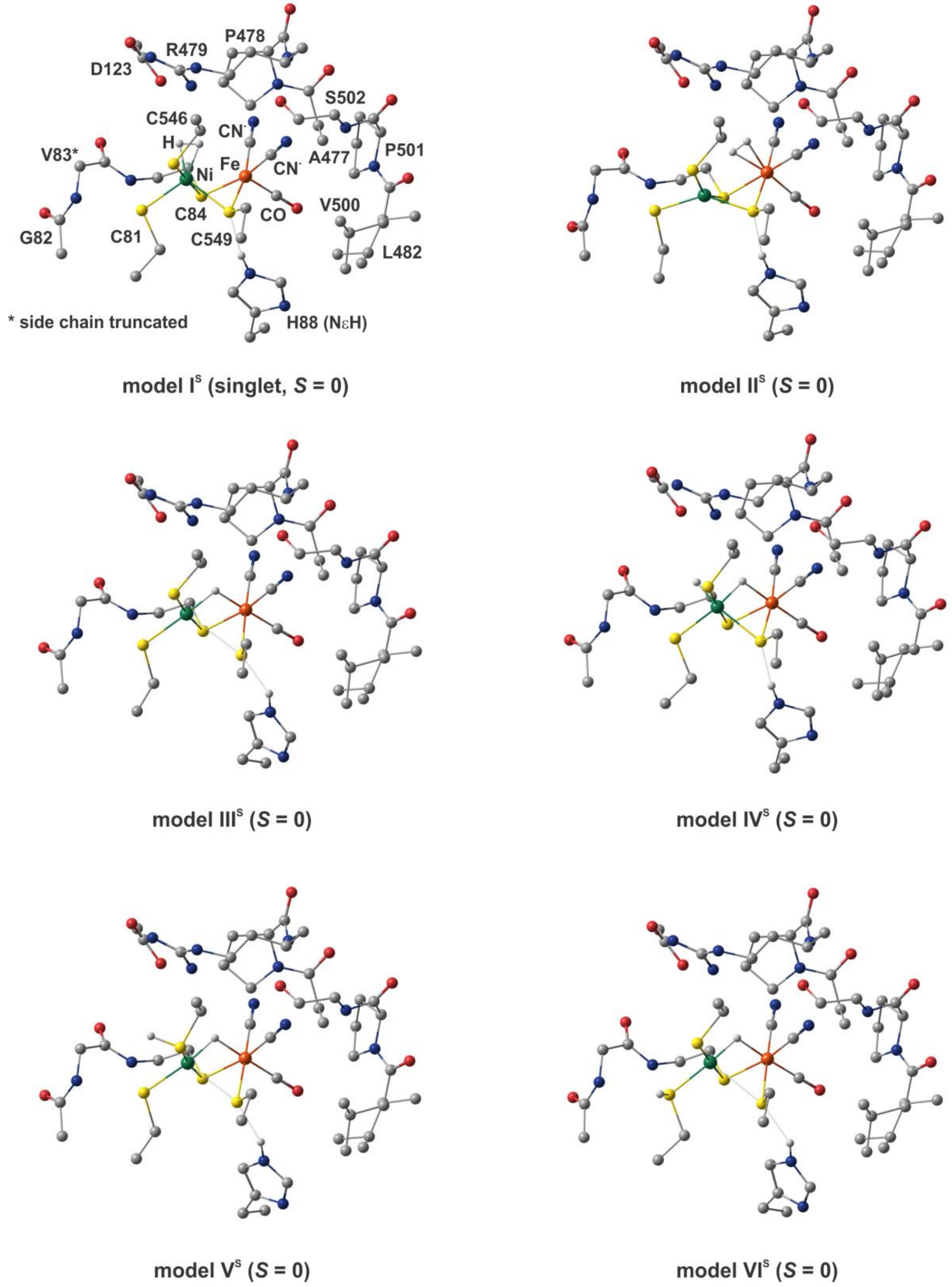

Supplementary Figure 20. Ball-and-stick representations of optimized low-spin enzyme cluster models.

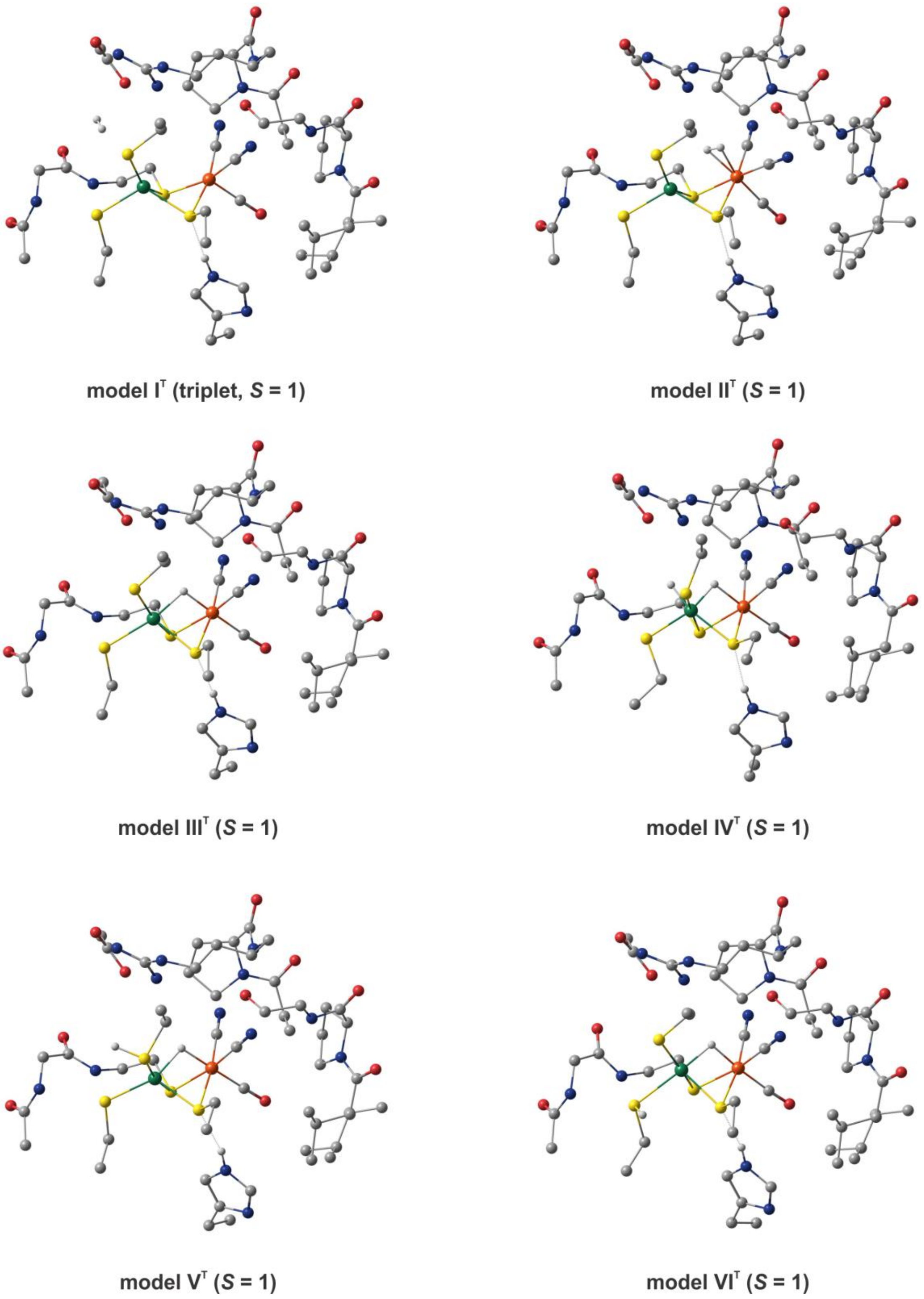

Supplementary Figure 21. Ball-and-stick representations of optimized high-spin enzyme cluster models.

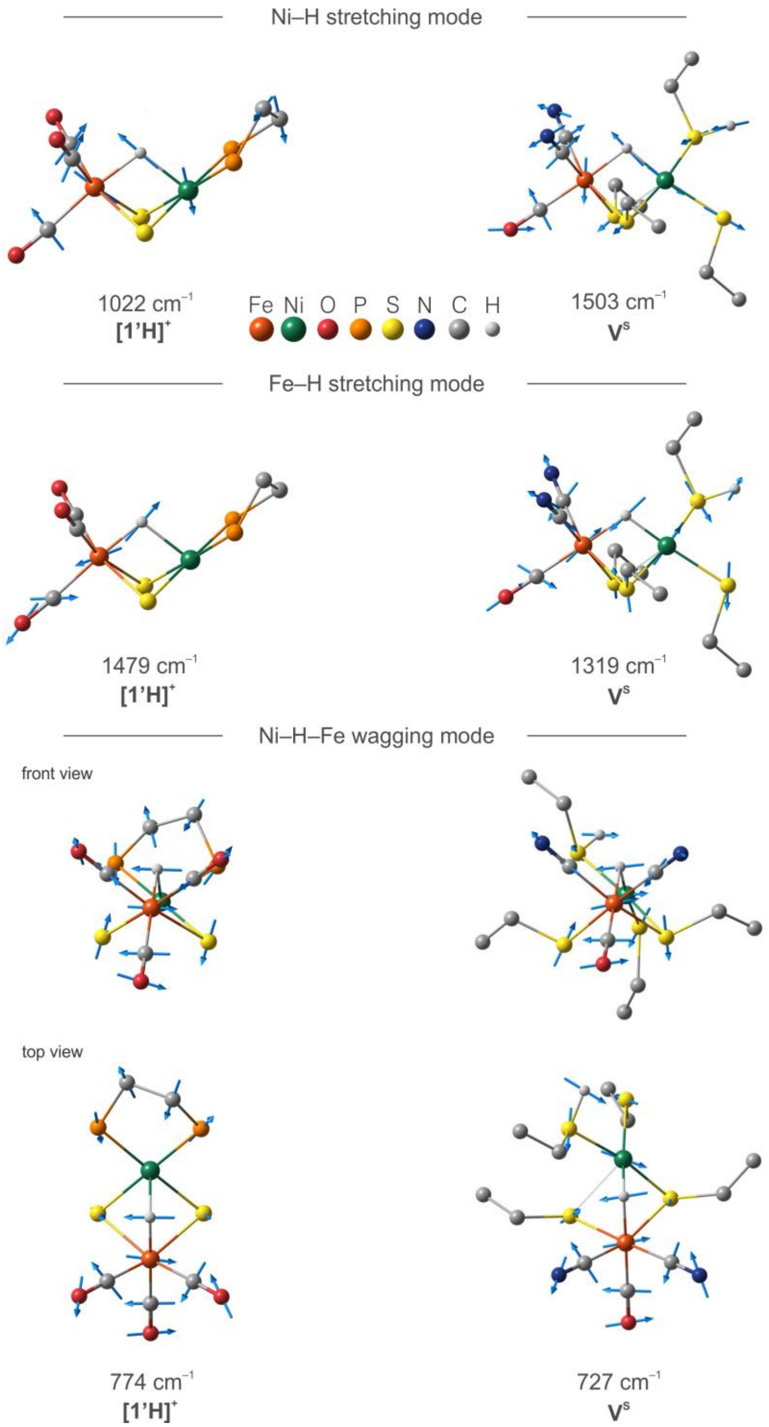

Supplementary Figure 22**.** Unscaled-arrow representations of the M–H normal modes calculated for the Ni-flippamer of [**1′**H]$^{+}$ (left) and Ni-R active site model $\mathbf{V}^{\mathbf{S}}$ (right), focusing on the direct coordination sphere of the dinuclear centers. Only the hydride bridge and the proton of Cys546 are shown, with other H atoms omitted for clarity. Displacement vectors for cysteinyl C atoms have been omitted for clarity.

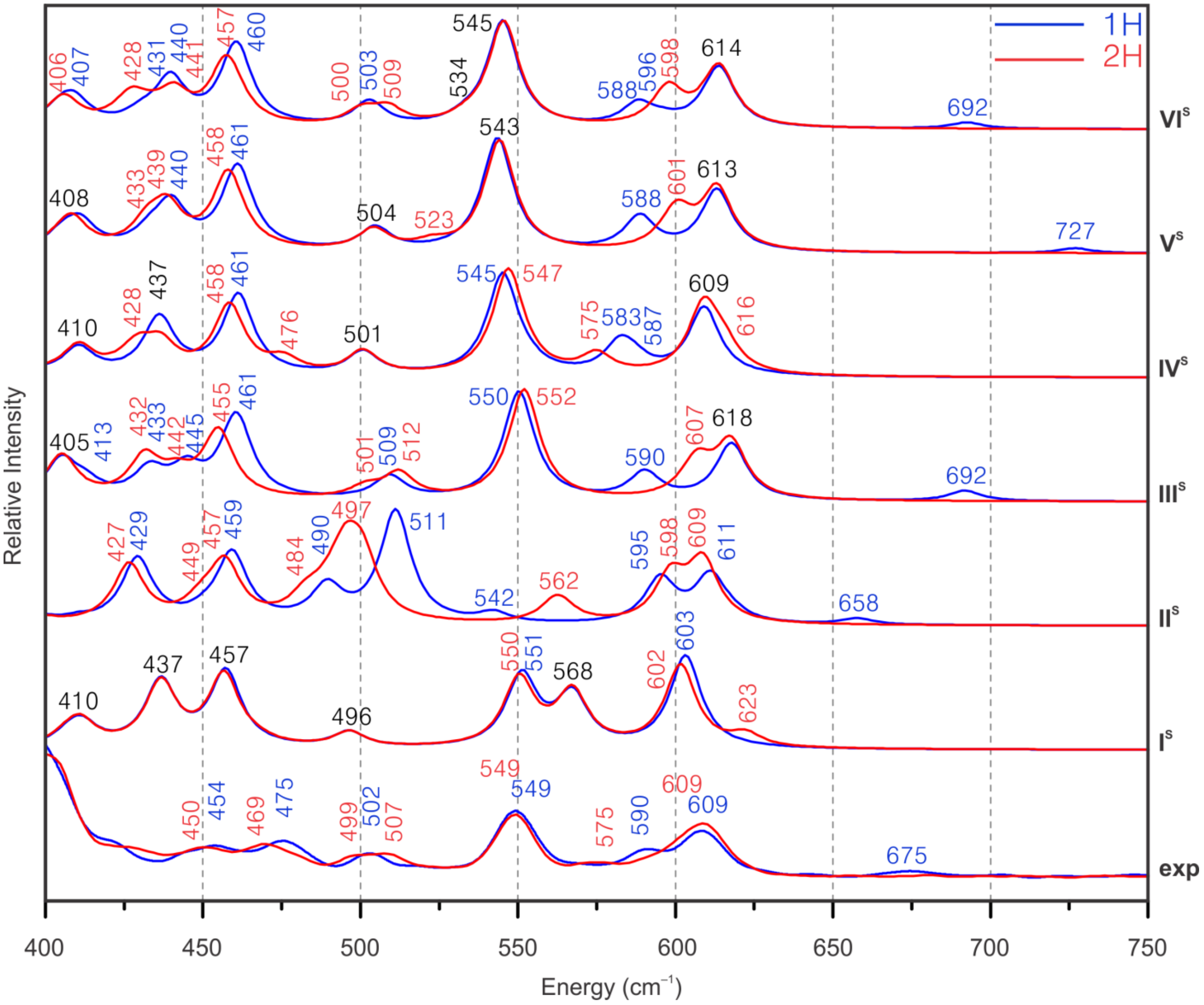

Supplementary Figure 23. Experimental NRVS for Ni-R and DFT-calculated spectra for S = 0 models (η^2-H_2)NiFe (**I^S**), NiFe(η^2-H_2) (**II^S**), Ni(μ-H)Fe (**III^S**), HNi(μ-H)Fe (**IV^S**), (Cys546)SHNi(μ-H)Fe (**V^S**), (Cys81)SHNi(μ-H)Fe (**VI^S**). H/D isotopomers are given in blue/red. The spectrum in H_2O is the average of three measurements at pH 5.0 while the spectrum in D_2O is the average of four at pD 5.0 and pD 7.8.

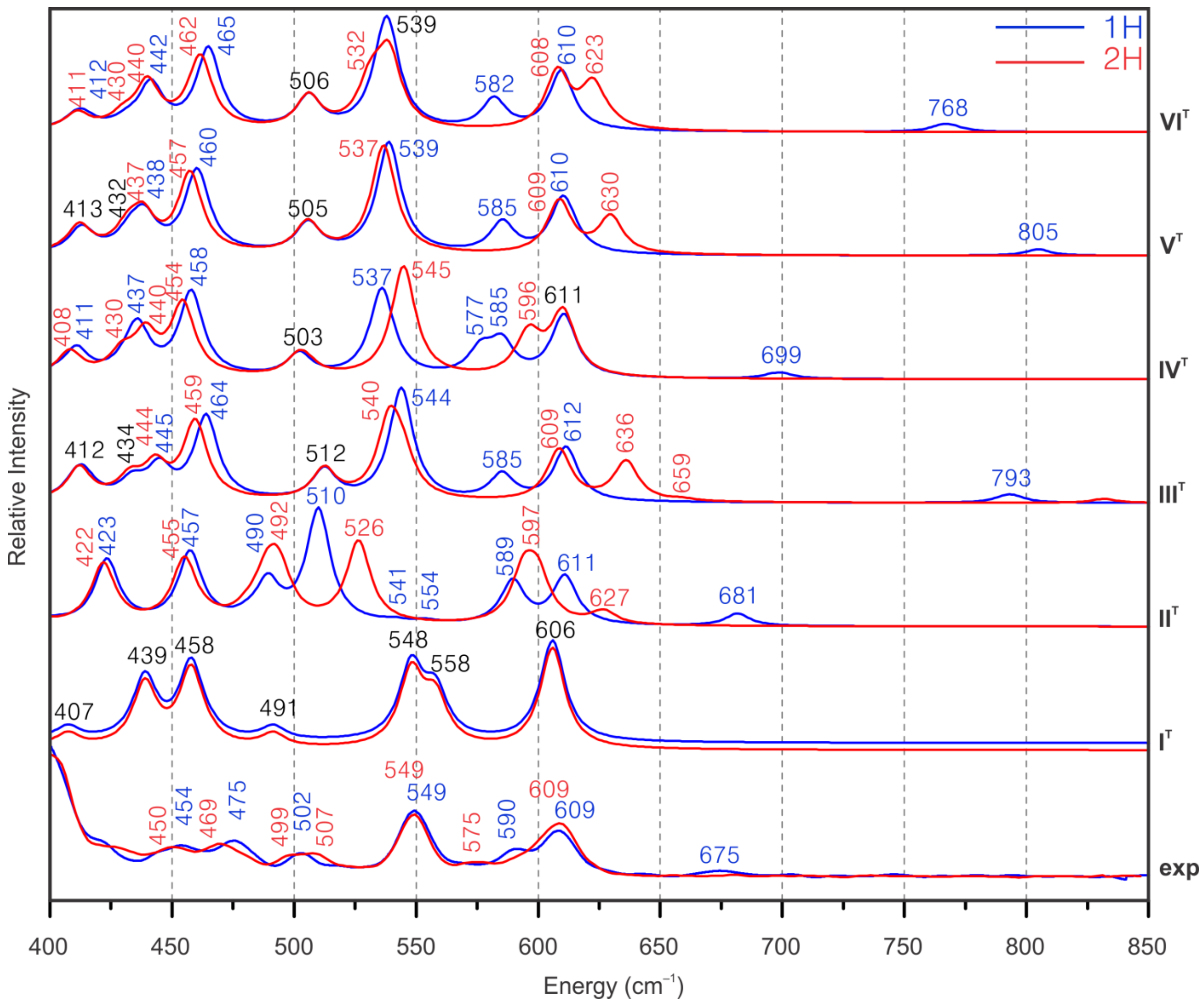

Supplementary Figure 24. Experimental NRVS for Ni-R and DFT-calculated spectra for *S* = 1 models (η^2-H_2)NiFe (**I^T**), NiFe(η^2-H_2) (**II^T**), Ni(μ-H)Fe (**III^T**), HNi(μ-H)Fe (**IV^T**), (Cys546)SHNi(μ-H)Fe (**V^T**), (Cys81)SHNi(μ-H)Fe (**VI^T**). H/D isotopomers are given in blue/red. The spectrum in H_2O is the average of three measurements at pH 5.0 while the spectrum in D_2O is the average of four at pD 5.0 and pD 7.8.

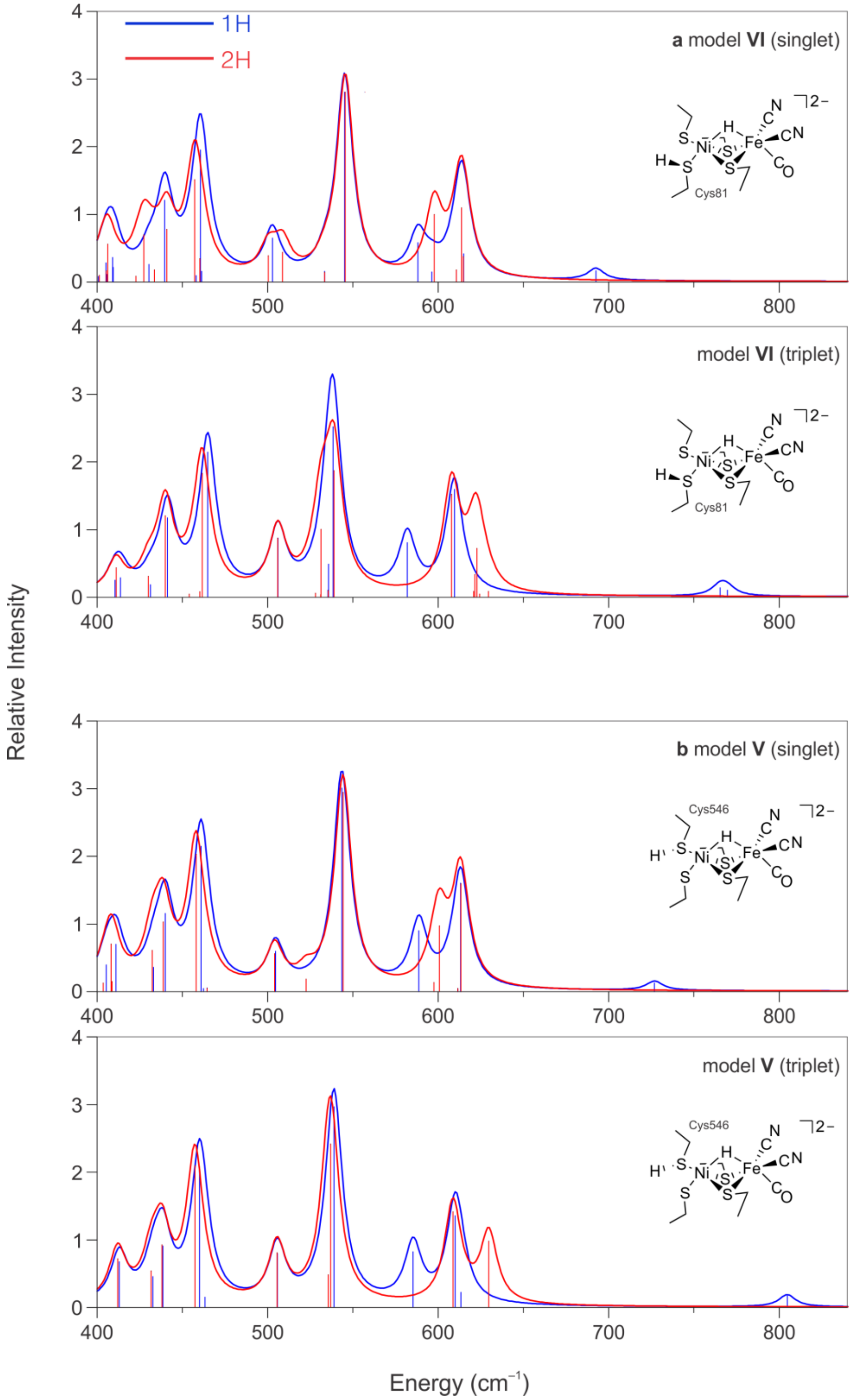

Supplementary Figure 25. DFT-predicted NRVS spectra of models **V** and **VI** as H/D isotopologues (blue and red traces, respectively). Vertical bars under the curves correspond to calculated mode composition factors of individual vibrational transitions.

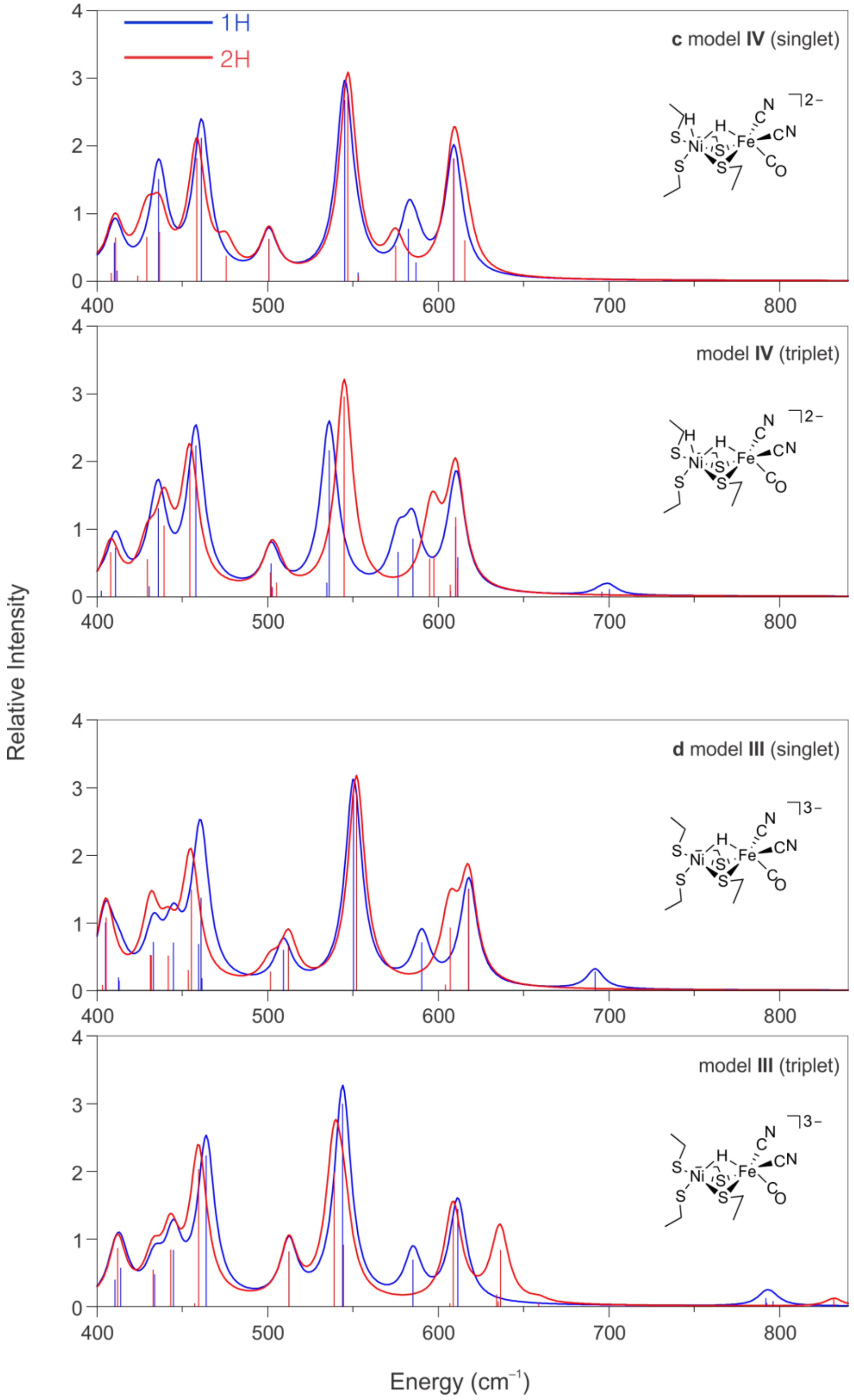

Supplementary Figure 26. DFT-predicted NRVS of structural models **III** and **IV** as H/D isotopologues (blue and red traces, respectively). Vertical bars under the curves correspond to calculated mode composition factors of individual vibrational transitions.

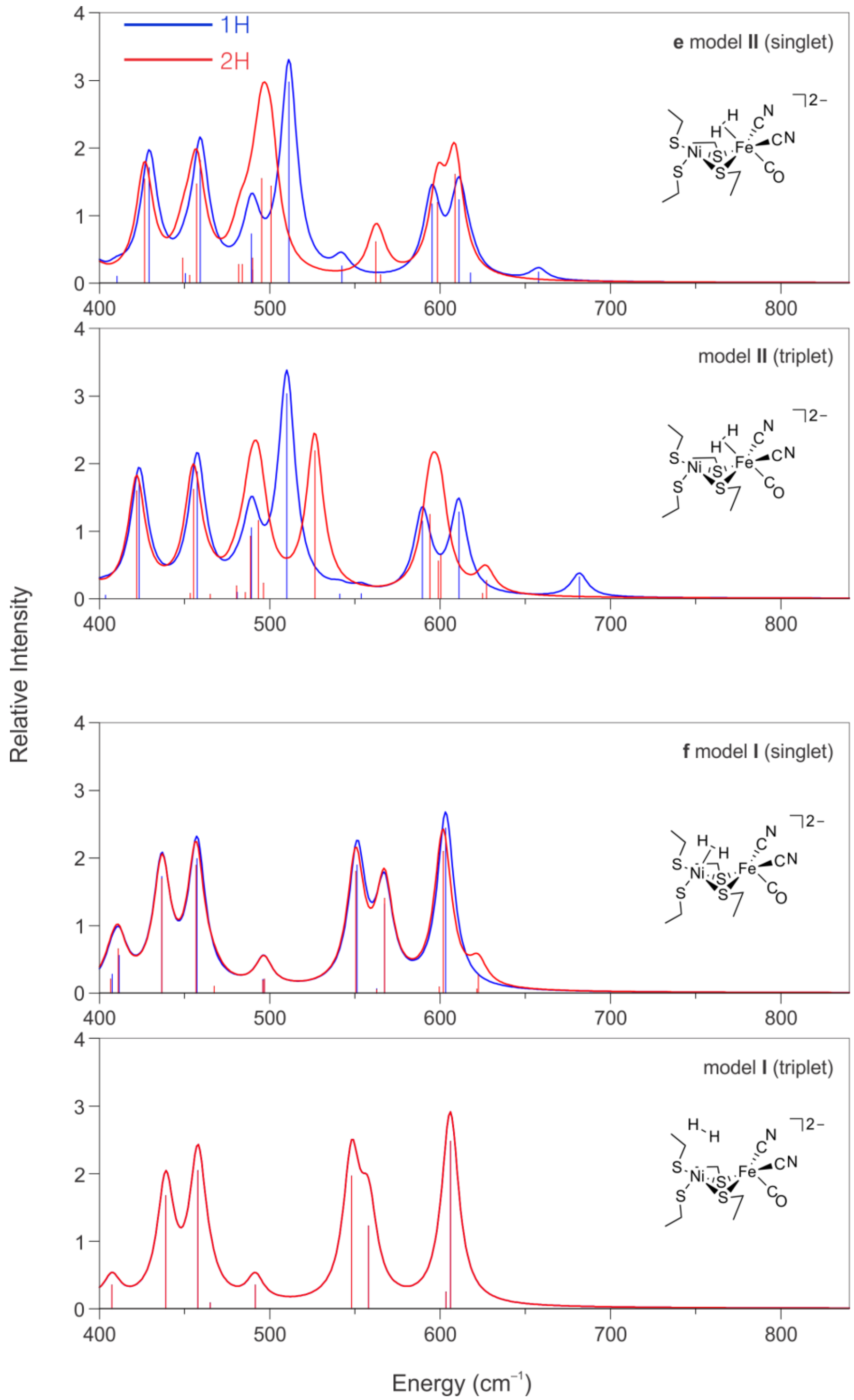

Supplementary Figure 27. DFT-predicted NRVS spectra of structural models **I** and **II** as H/D isotopologues (blue and red traces, respectively). Vertical bars under the curves correspond to calculated mode composition factors of individual vibrational transitions.

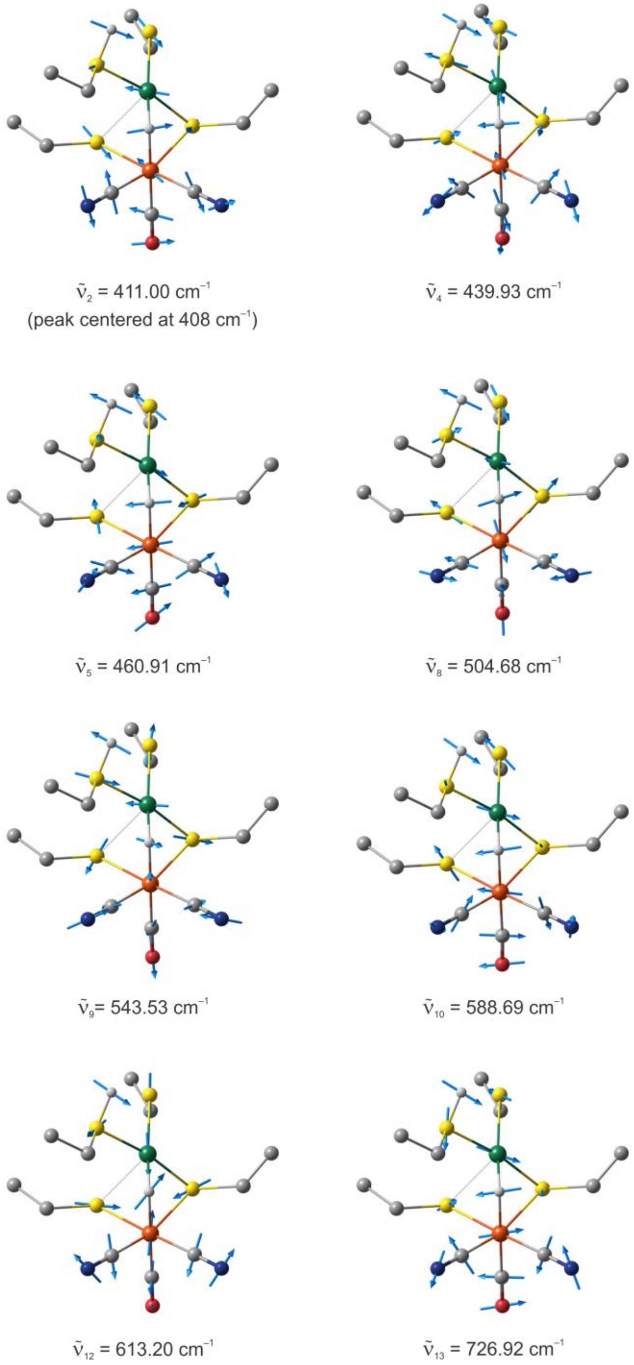

Supplementary Figure 28. Arrow representation of significant DFT-calculated normal modes for model $\mathbf{V^S}$ (H isotopologue) of Ni-R. Motions of the primary coordination sphere are shown, with the secondary coordination sphere omitted for clarity. Note that all modes are vibrationally coupled to the backbone and amino acid sidechains (see Supplementary Fig. 29). Animated representations of these modes are available as part of the Supplementary Information.

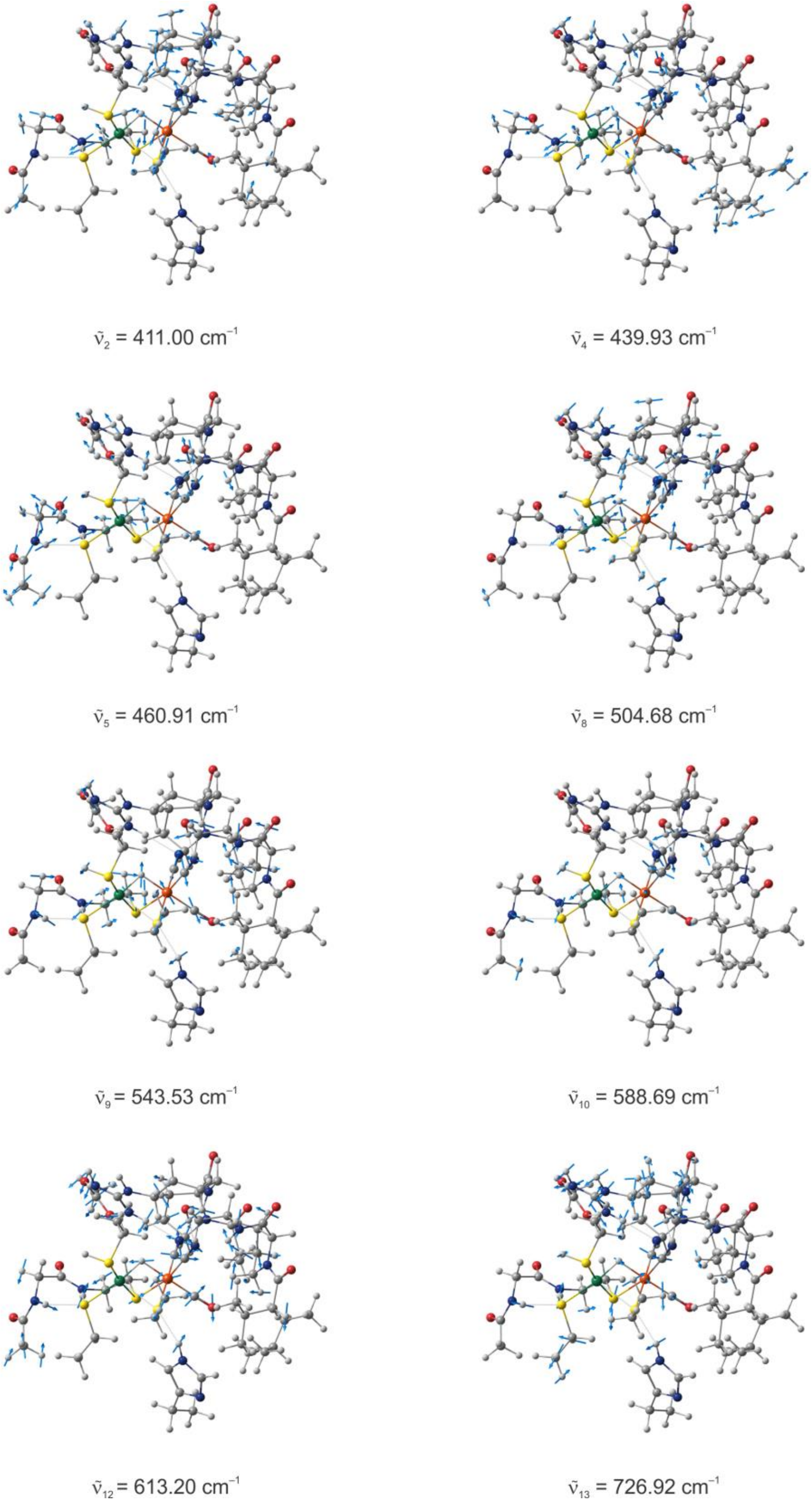

Supplementary Figure 29. DFT-calculated normal modes for model $\mathbf{V}^{\mathrm{S}}$ of Ni-R, showing the vibrational coupling of normal modes of the active site to backbone and amino acid side chains.

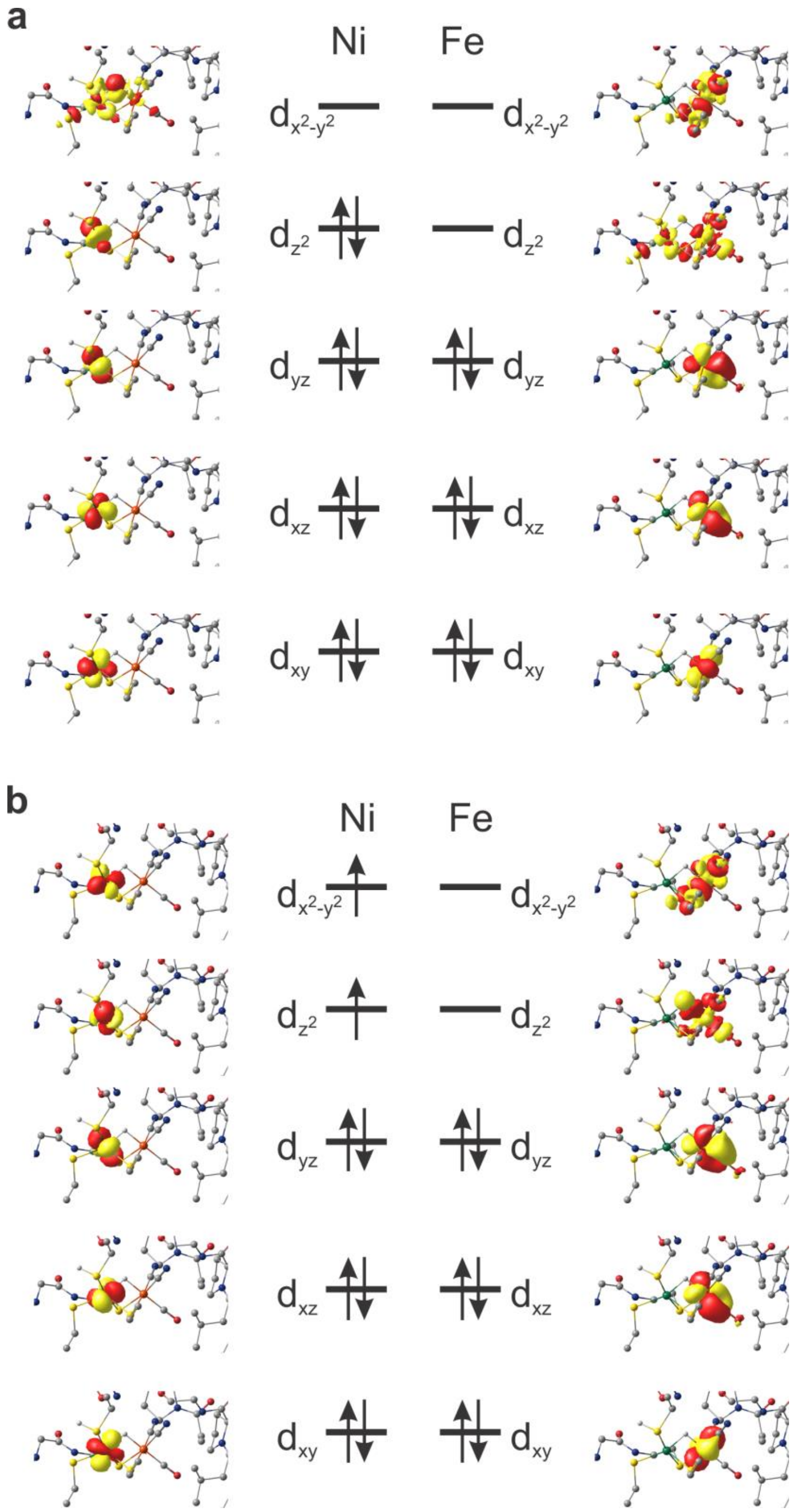

Supplementary Figure 30. Molecular orbital diagrams representing the electronic structure of singlet $\mathbf{V^S}$ (**a**) and triplet $\mathbf{V^T}$ (**b**) states of the (Cys546)SHNi(μ-H)Fe model. Orbitals correspond to localized (Pipek-Mezey scheme) canonical orbitals.

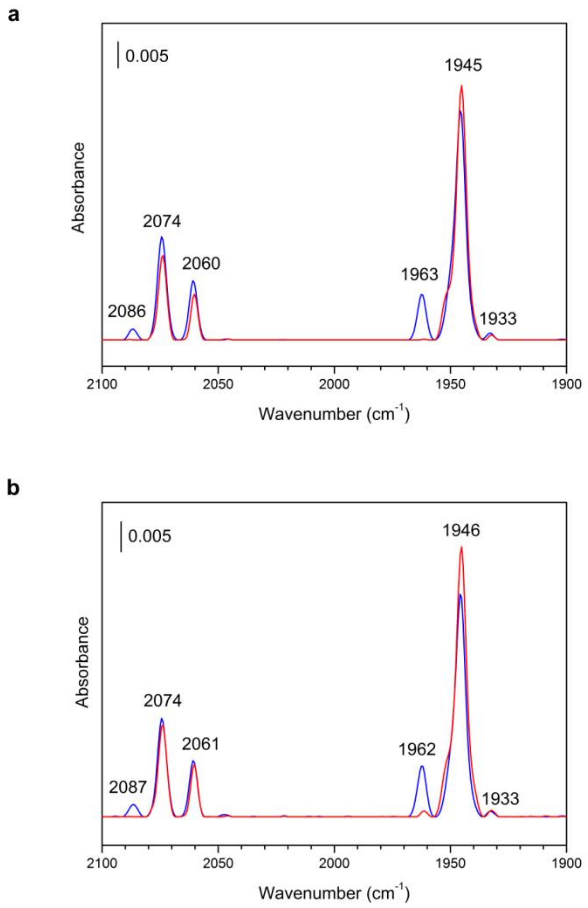

Supplementary Figure 31. FT-IR spectra of [NiFe]-hydrogenase samples in H_2O (blue trace) and D_2O (red trace) collected: **a**, before NRVS data collection and **b**, after NRVS data collection.

Supplementary Tables

Supplementary Table 1. Matching of the observed and DFT-calculated CO-coupled NRVS band positions (cm^{-1}) for model complexes **1′** (ref. [2]) and [**1′**H/D]$^+$.[a]

Model Complex	ν_{Fe-CO}	ν'_{Fe-CO}	ν''_{Fe-CO}	δ_{Fe-CO}	δ'_{Fe-CO}	δ''_{Fe-CO}
**1′**	-	469	496	557	588	613
DFT	-	*470*	*497*	*564*	*589*	*608*
[**1′**H]$^+$	440	462	495	558	587	617
DFT	*440* / *460*	*484* / *492*	*506* / *508*	*558* / *556*	*580* / *580*	*616* / *617*
[**1′**D]$^+$	436	460	495	-	580	608
DFT	*437* / *460*	*484* / *495*	*505* / *508*	-	*574* / *575*	*612* / *615*

[a] Corresponding [**1′**H/D]$^+$ spectra are shown in the main text Fig. 2 and Supplementary Fig. 18. The band positions from DFT are given in *italics*; for [**1′**H]$^+$ and [**1′**D]$^+$, calculated band positions for both the Ni/Fe-flippamers are provided in the corresponding order, separated by “ / ”.

Supplementary Table 2. Observed and DFT-calculated M–H/D band positions (cm^{-1}) for model complexes [**1**H/D]$^+$ and [**1′**H/D]$^+$.[a]

**M–H/D Band**	[**1′**H/D]$^{+\,b}$		[**1**H/D]$^{+\,c}$	
	**Observed**	***DFT*** *($e^2_{H/D}$)*	**Observed**	***DFT***
ν_{Fe-H}	1532 / 1468	*1479 (0.98)* / *1447 (0.98)*	1528 / 1470	*1672* / *1657*
ν_{Fe-D}	~1101 [d]	*1053 (0.94)* / *1030 (0.94)*	1096 / 1077	*1195* / *1185*
ν_{Ni-H}	954 / (1023) [e]	*1022 (0.91)* / *1061 (0.94)*	945 / 961	*959* / *984*
ν_{Ni-D}	708 / (752) [e]	*743 (0.83)* / *766 (0.85)*	N.D.	*716* / *730*
$\delta_{Ni-H-Fe}$	~758 [d]	*774 (0.77)* / *767 (0.83)*	N.D.	*N.D.*
$\delta_{Ni-D-Fe}$ [f]	mixed	*624 (0.13)* / *620 (0.13)*<br>*610 (0.22)* / *606 (0.18)*<br>*530 (0.23)* / *528 (0.22)*<br>*497 (0.15)* / *497 (0.17)*<br>*406 (0.21)* / *406 (0.20)*	N.D.	*N.D.*

[a] Band positions for the Ni- and Fe-flippamers, respectively, are provided in the corresponding order, separated by “ / “. Calculated band positions from DFT are given in *italics*.

[b] NRVS data and associated DFT modeling for ^{57}Fe-labeled complexes [**1′**H/D]$^+$ from the present work. Corresponding [**1′**H/D]$^+$ NRVS spectra are shown in the main text Fig. 2 and Supplementary Fig. 18. The normal mode composition factors from DFT ($e^2_{H/D}$) imply contribution of the H/D hydride motion to the total (e^2_{total} = 1) vibrational energy of a band.

[c] Raman data and associated DFT modeling for natural Fe abundance complexes [**1**H/D]$^+$ (ref. [1]). “N.D.” implies bands not determined or not defined.

[d] Ni/Fe-flippamer-dependent splitting could not be determined from NRVS experiment; only single band was observed.

[e] Tentative assignment for a low-intensity NRVS band.

[f] For the $\delta_{Ni-D-Fe}$ mixed wagging band, only DFT modes with the deuteride motion contribution to the vibrational energy above 10% (e^2_D > 0.1) are given.

Supplementary Table 3. Metal-metal and metal-ligand internuclear distances (Å) in [**1**H]$^+$ from X-ray data analysis[2] and DFT optimizations.*

	**X-ray Ni-flippamer**†	**DFT Ni/Fe-flippamer**†
**Fe–Ni**	2.614	2.652 / 2.659
**Fe–H**	1.460	1.607 / 1.611
**Ni–H**	1.638	1.799 / 1.782
**Fe–S1**	2.321	2.363 / 2.375
**Fe–S2**	2.321	2.370 / 2.375
**Fe–C1**	1.802	1.774 / 1.773
**Fe–C2**	1.798	1.803 / 1.807
**Fe–C3**	1.786	1.774 / 1.773
**Ni–S1**	2.210	2.261 / 2.256
**Ni–S2**	2.219	2.268 / 2.262
**Ni–P1**	2.173	2.226 / 2.223
**Ni–P2**	2.160	2.217 / 2.213

*Optimized structures for both Ni/Fe-flippamers and atom labels are shown in Supplementary Fig. 17.
†Only the Ni-flippamer structure is available from X-ray analysis[2].

Supplementary Table 4. DFT-calculated and observed wavenumbers in the range 400–750 cm^{-1} with ^{57}Fe normal mode composition factors ($e^2_{\mathrm{Fe}\alpha}$) for S = 0 model (Cys81)SHNi(μ-H)Fe (**VI**$^{\mathbf{S}}$). *This mode has significant Ni–H–Fe wagging character.

**mode**	**DFT wavenumbers ($e^2_{\mathrm{Fe}\alpha}$)**		**observed wavenumbers**	
	**H**	**D**	**H**	**D**
	$\tilde{\nu}_1$ 400.67 (0.007)			
	$\tilde{\nu}_2$ 405.20 (0.025)	401.28 (0.008)		
	$\tilde{\nu}_3$ 405.63 (0.010)	405.32 (0.014)		
	$\tilde{\nu}_4$ 409.09 (0.032)	406.20 (0.049)		
	$\tilde{\nu}_5$ 409.49 (0.019)			
		422.75 (0.008)		
Fe–CN symmetric stretch/bend	$\tilde{\nu}_6$ 430.41 (0.024)	427.43 (0.064)	454	450
	$\tilde{\nu}_7$ 439.68 (0.114)	433.64 (0.017)		
		440.82 (0.074)		
Fe–CN asymmetric stretch/bend	$\tilde{\nu}_8$ 458.05 (0.009)	457.11 (0.149)	475	469
	$\tilde{\nu}_9$ 460.61 (0.194)	460.28 (0.034)		
	$\tilde{\nu}_{10}$ 461.32 (0.016)			
Fe–CN scissor		500.43 (0.042)		499
	$\tilde{\nu}_{11}$ 502.78 (0.070)		502	
		**508.69 (0.048)***		507
Fe–CO stretch + Fe–CN symmetric bend	$\tilde{\nu}_{12}$ 533.33 (0.018)	533.35 (0.016)	549	549
	$\tilde{\nu}_{13}$ 545.14 (0.330)	545.50 (0.330)		
H–Fe–CN linear bend	$\tilde{\nu}_{14}$ 588.10 (0.074)	597.65	-	-
	$\tilde{\nu}_{15}$ 596.36 (0.019)	-	590	(575)
Fe–CO bend + Fe–CN symmetric bend	$\tilde{\nu}_{16}$ 610.70 (0.022)	610.63 (0.023)		
	$\tilde{\nu}_{17}$ 613.76 (0.141)	613.77 (0.145)	609	609
	$\tilde{\nu}_{18}$ 614.86 (0.055)	614.83 (0.050)		
Ni–H–Fe wag	$\tilde{\nu}_{19}$ 692.53 (0.025)	-	675	-

Supplementary Table 5. DFT-calculated and observed wavenumbers in the range 400–750 cm^{-1} with ^{57}Fe normal mode composition factors ($e^2_{Fe\alpha}$) for S = 0 model (Cys546)SHNi(μ-H)Fe ($\mathbf{V^S}$). *This mode has significant Ni–H–Fe wagging character.

mode	DFT wavenumbers ($e^2_{Fe\alpha}$)		observed wavenumbers	
	**H**	**D**	**H**	**D**
	$\tilde{\nu}_1$ 405.41 (0.034)<br>$\tilde{\nu}_2$ 411.00 (0.062)	403.54 (0.011)<br>408.20 (0.062)<br>408.72 (0.013)		
Fe–CN symmetric stretch/bend	$\tilde{\nu}_3$ 433.12 (0.033)<br>$\tilde{\nu}_4$ 439.93 (0.109)	432.27 (0.057)<br>438.72 (0.097)	454	450
Fe–CN asymmetric stretch/bend	$\tilde{\nu}_5$ 460.91 (0.212)<br>$\tilde{\nu}_6$ 462.36 (0.004)<br>$\tilde{\nu}_7$ 464.45 (0.005)	458.01 (0.200)<br>464.45 (0.005)	475	469
Fe–CN scissor	$\tilde{\nu}_8$ 504.68 (0.065)	504.14 (0.060)<br>**522.60 (0.021)***	502	499<br>507
$\nu_{Fe–CO}$ stretch (+ Fe–CN symmetric bend)	$\tilde{\nu}_9$ 543.53 (0.351)	544.16 (0.344)	549	549
H–Fe–CN linear bend	$\tilde{\nu}_{10}$ 588.69 (0.114)	597.46 (0.017)<br>600.64 (0.125)	-<br>590	-<br>(575)
$\delta_{Fe–CO}$ bend (+ Fe–CN symmetric bend)	$\tilde{\nu}_{11}$ 611.51 (0.006)<br>$\tilde{\nu}_{12}$ 613.20 (0.211)	611.51 (0.006)<br>613.26 (0.211)	609	609
Ni–H–Fe wag	$\tilde{\nu}_{13}$ 726.92 (0.020)	-	675	-

Supplementary Notes

Supplementary Note 1

(dppe)Ni(μ-pdt)^{57}Fe(^{13}CO)$_3$ (1″)

^{13}C{^{1}H} NMR (CH_2Cl_2) 207.2 ppm (d, $^1J_{PFe}$ = 26 Hz). ^{31}P{^{1}H} NMR (CH_2Cl_2) 63.0 ppm. FT-IR (CH_2Cl_2): ν_{CO} = 1982, 1913 cm^{-1}. ESI-MS: *m*/*z* 705.7 $[M]^+$ (ionization induced by addition of $FcBF_4$). Anal. calcd for $^{13}C_{32}H_{30}O_3S_2P_2Ni^{57}Fe$: C, 54.33; H, 4.28; N, 0.00. Found: C, 54.36; H, 3.87; N, 0.00.

[(dppe)Ni(μ-pdt)(μ-H)^{57}Fe(CO)$_3$]BF$_4$ ([1′H]BF$_4$)

^{1}H NMR (CD_2Cl_2) –3.46 ppm (m). ^{31}P{^{1}H} NMR (CD_2Cl_2) 69.2 ppm. FT-IR (CD_2Cl_2): ν_{CO} = 2083, 2024 cm^{-1}. ESI-MS: *m*/*z* 704.0 $[M - BF_4^-]^+$.

[(dppe)Ni(μ-pdt)(μ-H)^{57}Fe(^{13}CO)$_3$]BF$_4$ ([1″H]BF$_4$)

Yield: 85%, orange powder. ^{1}H NMR (CD_2Cl_2) –3.50 ppm (d, J_{HC} = 10 Hz). ^{13}C{^{1}H} NMR (CD_2Cl_2) 205.1 (dt, $^1J_{CFe}$ = 26 Hz, $^2J_{CC}$ = 4 Hz, 1C), 204.2 (dd, $^1J_{CFe}$ = 26 Hz, $^2J_{CC}$ = 4 Hz, 2C) ppm. ^{31}P{^{1}H} NMR (CD_2Cl_2) 69.2 ppm. FT-IR (CD_2Cl_2): ν_{CO} = 2035, 1978 cm^{-1}. ESI-MS: *m*/*z* 707.0 $[M - BF_4^-]^+$.

[(dppe)Ni(μ-pdt)(μ-D)^{57}Fe(CO)$_3$]BF$_4$ ([1′D]BF$_4$)

^{2}H NMR (CH_2Cl_2) –3.4 ppm (m). ^{31}P{^{1}H} NMR (CH_2Cl_2) 69.2 ppm. FT-IR (CH_2Cl_2): ν_{CO} = 2083, 2023 cm^{-1}. ESI-MS: *m*/*z* 705.0 $[M - BF_4^-]^+$.

[(dppe)Ni(μ-pdt)(μ-D)^{57}Fe(^{13}CO)$_3$]BF$_4$ ([1″D]BF$_4$)

Yield: 85%, orange powder. ^{2}H NMR (CH_2Cl_2) –3.4 ppm (m). ^{13}C{^{1}H} NMR (CH_2Cl_2) 204.6 (dm, $^1J_{CFe}$ = 24 Hz, 1C), 203.7 (dm, $^1J_{CFe}$ = 24 Hz, 2C) ppm. ^{31}P{^{1}H} NMR (CH_2Cl_2) 69.2 ppm. FT-IR (CH_2Cl_2): ν_{CO} = 2035, 1978 cm^{-1}. ESI-MS: *m*/*z* 708.1 $[M -BF_4^-]^+$.

Supplementary Discussion

Further Discussion on Model Complex

With the bridging hydride in focus, structural parameters of the NiFe core in [**1′**H]$^{+}$ deserve further attention. Metal-metal and metal-ligand internuclear distances listed in Supplementary Table 3 display deviations between the X-ray diffraction data[2] and theory of ~0.06 Å at most, except those for the M–H distances. Both the Ni–H and Fe–H distances are predicted to be ~0.14-0.16 Å consistently longer by DFT. Similar trends have been outlined in the earlier DFT study on [**1′**H]$^{+}$; a systematic underestimation of M–H bond distances by X-ray diffraction has been claimed as an additional source for the above discrepancy[1].

As illustrated in the main text Fig. 2 and Supplementary Fig. 17, the central methylene of the $^{-}SCH_2\mathit{CH_2}CH_2S^{-}$ (pdt^{2-}) ligand can be oriented either toward Ni ('Ni-flippamer') or Fe ('Fe-flippamer'). Both present and previous[3] DFT calculations predict the Ni-flippamer to be more stable than the Fe-flippamer by ~3 kcal mol^{-1}. With this energy difference, which is close to the DFT accuracy limits, the Ni-flippamer is nevertheless expected to dominate in the NRVS sample. Examination of the high-end NRVS spectra (the main text Fig. 2 and Supplementary Fig. 18 and Supplementary Table 2) clearly resolves the Ni/Fe-flippamer dependent splitting of ν_{Fe-H} = 1532/1468 cm^{-1} ($\Delta\nu_{Fe-H}$ = −64 cm^{-1}) by the experiment and ν_{Fe-H} = 1479/1447 cm^{-1} ($\Delta\nu_{Fe-H}$ = −32 cm^{-1}) by DFT. The relative ν_{Fe-H} bands intensities indicate nearly equal populations for the Ni/Fe-flippamers. For the lower energy Ni–H stretching band, ν_{Ni-H} = 954/1023 cm^{-1} ($\Delta\nu_{Ni-H}$ = 69 cm^{-1}) by the experiment and ν_{Ni-H} = 1022/1061 cm^{-1} ($\Delta\nu_{Ni-H}$ = 39 cm^{-1}) by DFT, the observed NRVS intensities however correspond to a significantly lower population of the alternative Fe-flippamer. A similar conclusion on underrepresentation of the Fe-flippamer can be deduced from examination of the 700-770 cm^{-1} Ni-D bands in the [**1′**D]$^{+}$ and [**1″**D]$^{+}$ isotopologues data (Supplementary Fig. 18). The Ni–H–Fe wag mode predicted at 774/768 cm^{-1} has only a small flippamer-dependent splitting ($\Delta_{Ni-H-Fe}$ = −6 cm^{-1}), which could not be observed in the experiment. Among the high intensity NRVS bands <700 cm^{-1} (recorded for all the four isotopologues, see Supplementary Fig. 18), the 440-630 cm^{-1} Fe–CO region does not reveal a flippamer preference; finally, in the low frequency region <400 cm^{-1}, the observed bands positions and intensities are in noticeably better agreement with the Ni-flippamer DFT modeling. In conclusion, our evaluation of the observed and calculated spectra indicates variable concentrations of both the [**1′**H]$^{+}$ Ni/Fe-flippamers in

the NRVS samples. This result is in line with variance from earlier interpretations: while $[\mathbf{1}H]^+$ crystallizes as the Ni-flippamer[4], Raman spectroscopy indicated that both conformations are present in frozen solution[5].

As suggested by the values above, magnitudes of the flippamer-dependent $\Delta\nu_{M-H}$ splittings from DFT are significantly (but consistently) underestimated by ~30 cm^{-1}: $\Delta\nu_{Fe-H} = -64/-32$ cm^{-1} and $\Delta\nu_{Ni-H} = 69/39$ cm^{-1} from the observed/calculated spectra, respectively. This may be rationalized by the consistent (see above) overestimation of the equilibrium Ni/Fe–H bond lengths by DFT, and 'softer' calculated potential for the hydride motion as an outcome. Additionally, the *inverted* character of the flippamer-dependent $\Delta\nu_{M-H}$ shifts (*i.e.*, negative for ν_{Fe-H} and positive for ν_{Ni-H}) can be rationalized in view of Badger's rule applied to a fine yet noticeable hydride position shift: the optimized Fe–H bond becomes 0.004 Å longer and Ni–H bond becomes 0.017 Å shorter when going from the Ni- to Fe-flippamer (Supplementary Table 3).

Further Discussion of Enzyme Cluster Models

The spectra simulated for singlet $\mathbf{V^S}$ and $\mathbf{VI^S}$ most closely matched the experimentally determined peak positions, intensity distributions, splitting pattern and H/D isotope effects. Interestingly, the calculated NRVS is relatively insensitive to the protonation state of terminal cysteinates, as the results for $\mathbf{III^S}$ are almost identical to those for $\mathbf{V^S}$ and $\mathbf{VI^S}$ (Supplementary Fig. 23). Therefore, discrimination between models based on NRVS data (in the Fe–CO/CN region) alone is impossible. However, considering our previous FT-IR studies on Ni-R, and the relative intensities of ν_{CO} and ν_{CN} modes simulated for the models, we conclude that $\mathbf{III^S}$, which bears unprotonated cysteinates, is an implausible depiction of the Ni–R active site[6].

Agreement between DFT calculations and experiment is much less satisfactory when examining Ni-R models other than $\mathbf{V^S}$ and $\mathbf{VI^S}$ (Supplementary Fig. 23). Most strikingly, the distinct Ni–H–Fe wagging mode expected around 700 cm^{-1} is absent from simulated NRVS data (the only exception being $\mathbf{III^S}$). Interestingly, although model $\mathbf{IV^S}$ is predicted to exhibit an IR-active vibration of mainly Ni–H–Fe wagging character at higher energy (840 cm^{-1}), this mode remains absent from the NRVS.

In addition to the absence of a wagging mode for the (η^2-H_2)NiFe model $\mathbf{I^S}$, the DFT-calculated spectrum does not reveal any isotope effect in the low energy region. This is rationalized in terms of H_2 binding strongly to Ni and interacting only negligibly with Fe (the distance between Fe and the closest H

atom is 2.46 Å). In contrast, the strong Fe–(η^2-H_2) interaction in $\mathbf{II^S}$ results in additional spectral features (Supplementary Fig. 23). While spectra of $\mathbf{II^S}$ are sensitive to H/D exchange, peak positions/intensities and isotope shifts completely disagree with experimental data. It is also important to note that the peak feature at 658 cm^{-1} in the simulated spectrum of $\mathbf{II^S}$ does not correspond to the characteristic Ni–H–Fe wag, but rather a distinctly different wagging mode of the Fe–H_2 moiety.

Considering the overall contour of the calculated spectra, it is evident that none of the models in which Ni(II) is high-spin (S = 1) can give rise to the experimental NRVS fingerprint (see Supplementary Fig. 24). The key Ni–H–Fe wagging mode observed for Ni-R, while present in simulated spectra for $\mathbf{V^T}$ and $\mathbf{VI^T}$, is shifted to higher energy (relative to singlet $\mathbf{V^S}$ and $\mathbf{VI^S}$) by ~80 cm^{-1}, far away (>100 cm^{-1}) from the band of the native Ni-R ($\mathbf{III^T}$ is a similarly poor spectroscopic model). These results highlight the fact that the exact position of the wagging mode in the spectrum strongly depends on the electronic structure of the active site and thus the primary coordination sphere. The energy of this mode is strongly correlated with the degree of 'planarity' of the Ni coordination sphere (as measured by the dihedral angle between the Cys81–Ni–Cys546 and H–Ni–Cys84 planes) and, to a lesser extent, the M–H bond distances. The latter indicates that the wag frequency may serve as a diagnostic of whether the wag has more Ni–H or Fe–H character.

Prominent peak features at 623 ($\mathbf{VI^T}$), 630 ($\mathbf{V^T}$) and 636 cm^{-1} ($\mathbf{III^T}$) in the spectra of the D-isotopologue result in splitting patterns in the high energy region at odds with observed data. Although $\mathbf{IV^T}$ exhibits a wag at 699 cm^{-1}, the predicted H/D isotope splitting of bands at 537 and 545 cm^{-1} also renders this model inconsistent with experiment. Similar arguments hold for $\mathbf{II^T}$. Finally, H_2 does not bind to Ni in the high-spin model $\mathbf{I^T}$ and dissociation of H_2 is observed[3]. As a result, the calculated NRVS spectrum features vibrational signatures of the $Fe(CN)_2(CO)$ moiety but lacks the important Ni–H–Fe wagging mode. As would be expected, no isotope effect is observed.

A detailed depiction of contributions from individual normal modes (stick plots) to the bands in the NRVS spectra of all models is shown in Supplementary Figs. 25-27.

Electronic Structure of Model V

Electronic structures of the singlet and triplet states of model (Cys546)SHNi(μ-H)Fe (**V**) have been analyzed by means of the Pipek-Mezey localized orbital scheme (Supplementary Fig. 30). In the singlet state ($\mathbf{V^S}$), the Ni adopts an almost square-planar coordination with a weak bonding interaction between

Ni and S of Cys549 (Ni–S 2.75 Å), while the remaining bond lengths between the Ni and the in-plane S atoms are in the range 2.21–2.25 Å. The geometry about Fe is octahedral. The bridging H^- forms part of both these coordination spheres, occupying the axial position at Fe, and a basal Ni site. Binding of the hydride to the metal centers is asymmetric, reflected in the short Ni–H bond distance (1.57 Å) relative to the longer Fe–H bond distance (1.71 Å), indicating stronger bonding between H^- and Ni. The Ni–H–Fe bond angle is 104.5°. In terms of the orbital analysis, the presence of a doubly unoccupied $d_{x^2-y^2}$ orbital but an otherwise completely filled 3*d* orbital manifold on the Ni site according to the electronic configuration $(d_{xy})^2(d_{xz})^2(d_{yz})^2(d_{z^2})^2$ is fully consistent with low-spin Ni(II) (d^8). Likewise, the $(t_{2g})^6$ configuration (assuming local quasi-octahedral O_h symmetry) found on the Fe center is typical for low-spin Fe(II) (d^6). In the triplet state (**V$^{\mathbf{T}}$**) iron remains low-spin, while the actual change of the spin state takes place on the Ni center. This ion now adopts a local high-spin $(d_{z^2})^1(d_{x^2-y^2})^1$ electronic configuration. Mulliken spin populations of 1.40 and 0.06 on Ni and Fe, respectively, accompanied by substantial spin delocalization onto the ligand S atoms ($\rho_S \approx 0.5$), are fully consistent with this notion. Conversion from low-spin to high-spin causes notable changes in the core geometry. The bond distance between Ni and S of Cys549 significantly shortens (Ni–S 2.35 Å), this cysteinyl thiolate occupying the axial position of the now distorted square-pyramidal center. The octahedral Fe coordination is preserved. The remaining M–S bonds, involving Cys546, Cys81 and Cys84, become elongated (2.31–2.44 Å). Importantly, the position of the H^- bridge is also affected, resulting in an expansion of the Ni–H bond distance (1.71 Å) and simultaneously, contraction of the Fe–H bond distance (1.65 Å). The weakening of the Ni–H interaction is accompanied by a strengthening of the Fe–H bond (also clearly reflected in the Fe–H and Ni–H stretching frequencies and their force constants).

Supplementary Methods

Synthetic Model Compounds

Overview

Low-valent Ni(I)Fe(I) complexes such as (dppe)Ni(μ-pdt)Fe(CO)$_3$ (**1**) serve as valuable precursors to Ni(II)Fe(II) [NiFe]-hydrogenase models such as tricarbonyl salt [(dppe)Ni(μ-pdt)(μ-H)Fe(CO)$_3$]BF$_4$ ([**1**H]BF$_4$), formed by action of HBF$_4$ on **1**. The recently reported synthesis of the ^{57}Fe-labeled derivative (dppe)Ni(μ-pdt)^{57}Fe(CO)$_3$ (**1′**) thus affords convenient access to labeled hydrides such as [(dppe)Ni(μ-pdt)(μ-H)^{57}Fe(CO)$_3$]BF$_4$ ([**1′**H]BF$_4$, Supplementary Fig. 1) for spectroscopic investigations. The ^{1}H NMR resonance for the H$^-$ ligand in this compound is broadened relative to the natural abundance species owing to a weak ^{57}Fe-^{1}H interaction, ESI-MS data being consistent with high isotopic purity.

With respect to the NRVS study, it seemed likely that δ_{FeCO} bending modes would preclude the resolution of key H-coupled vibrations. The salt [(dppe)Ni(μ-pdt)(μ-H)^{57}Fe(^{13}CO)$_3$]BF$_4$ ([**1″**H]BF$_4$) was thus targeted to allow for bands of interest to be resolved and assigned. While hydride [**1**H]BF$_4$ undergoes only sluggish exchange with ^{13}CO, **1** is rapidly converted to (dppe)Ni(μ-pdt)Fe(^{13}CO)$_3$ under ambient temperature and ^{13}CO (1 atm)[7]. Analogously, **1′** was converted to ^{57}Fe- and ^{13}CO-labeled species (dppe)Ni(μ-pdt)^{57}Fe(^{13}CO)$_3$ (**1″**), which exhibits ν_{CO} bands identical to those of (dppe)Ni(μ-pdt)Fe(^{13}CO)$_3$. Further analyses by ESI-MS ([**1″**]$^+$ @ *m*/*z* 705.7), and ^{31}P{^{1}H} NMR (δ 63.0, broad) are particularly telling. Also of interest was the ^{13}C{^{1}H} NMR spectrum of **1″**, which features an intense doublet at δ 207.2 ($^1J_{CFe}$ = 26 Hz) assigned to the ^{13}CO ligands. The single resonance for these ligands indicates the rapid turnstile rotation of the (^{13}CO)$_3$Fe fragment at room temperature, while the magnitude of the ^{13}C-^{57}Fe coupling is similar to that in the (fluxional) iron carbonyls Fe(CO)$_5$ (23 Hz) and Fe(CO)$_4$PEt$_3$ (26 Hz). Treatment of **1″** with HBF$_4$ furnished [**1″**H]BF$_4$ according to FT-IR and ESI-MS, as well as ^{1}H, ^{13}C{^{1}H} and ^{31}P{^{1}H} NMR data. Most notable was the observation of two ^{13}CO signals, assigned to the apical and basal carbonyl ligands. Thus, in contrast to their Ni(I)Fe(I) precursors, the hydrides exhibit turnstile rotation which is slow on the NMR timescale, a fact that might explain the slow reaction of [**1**H]BF$_4$ with ^{13}CO. The apical and basal ^{13}CO ligands in [**1″**H]BF$_4$ couple equally strongly to the ^{57}Fe center ($^1J_{CFe}$ = 26 Hz), while the ^{13}C-^{13}C interaction is less prominent ($^2J_{CC}$ = 4 Hz).

In order to confirm the assignment of $NiH^{57}Fe$ modes by NRVS, the deuterium-labeled analogs [**1′**D]BF_4 and [**1″**D]BF_4 were prepared, lower energy bands being expected in these cases. Routine characterization data for deuterides were similar to those for the hydrides, with ESI-MS (Supplementary Fig. 15) and ^{2}H NMR analyses being most diagnostic of successful deuteration.

Preparative Details

Unless otherwise stated, all chemicals were purchased from commercial sources and used as received. Chromatography was performed using SiO_2 (40 - 63 μm, 230 - 400 mesh) as the stationary phase. CD_2Cl_2 was distilled from CaH_2. The labeled complex (dppe)Ni(μ-pdt)^{57}Fe(CO)$_3$ (**1′**) was prepared according to the literature methods[8]. All manipulations not involving ^{13}CO were conducted in an MBraun glovebox equipped with a solvent purification system; the concentrations of O_2 and H_2O in the N_2 atmosphere were typically no higher than 2 and 0.2 ppm, respectively. Glassware used in the preparation of deuterides [**1′**D]BF_4 and [**1″**D]BF_4 was washed with D_2O and dried prior to use. Solution IR spectra were recorded on a Perkin-Elmer Spectrum 100 FT-IR spectrometer. A Waters Micromass Quattro II spectrometer was used to acquire ESI-MS data for analytes in dilute CH_2Cl_2 or CD_2Cl_2 solution. Analytical data were acquired using an Exeter Analytical CE-440 elemental analyzer. NMR data were acquired at room temperature, with samples under an atmosphere of N_2. ^{1}H, ^{13}C{^{1}H} and ^{31}P{^{1}H} NMR spectra were recorded on a Varian VXR500 spectrometer at 500, 126 and 202 MHz, respectively. ^{2}H NMR spectra were recorded on a Varian UI500NB spectrometer at 77 MHz. Chemical shifts (in ppm) are referenced to $CHDCl_2$/CH_2Cl_2 (5.32 ppm for ^{1}H, ^{2}H, 53.84 ppm for ^{13}C), and external 85% H_3PO_4 (0 ppm for ^{31}P).

(dppe)Ni(μ-pdt)^{57}Fe(^{13}CO)$_3$ (1″)

A solution of **1′** (14.1 mg, 20 μmol) in CH_2Cl_2 (2 mL) was frozen with liquid N_2. The reaction vessel was evacuated; the solution was placed under ^{13}CO (1 atm) and allowed to warm to room temperature. The mixture was briefly agitated before being allowed to stand for 2 h, after which the solution was once again frozen and the headspace replaced with fresh ^{13}CO (1 atm). After 2 h at room temperature, the solution was chromatographed (~4 cm SiO_2, CH_2Cl_2 eluent) and the green band collected and treated with CH_3CN (5 mL). The solution was concentrated to ~1 mL, whereupon a solid formed. The material was isolated by filtration, washed with CH_3CN (2 × 5 mL), Et_2O (2 × 5 mL) and pentane (2 × 5 mL),

and dried briefly to afford the title compound as dark green crystals (11.5 mg, 16 µmol, 81%) (Supplementary Figs. 2-4).

[(dppe)Ni(µ-pdt)(µ-H)^{57}Fe(CO)$_3$]BF$_4$ ([1′H]BF$_4$)

A solution of **1′** (10.6 mg, 15 µmol) in CH_2Cl_2 (1 mL) was treated with HBF_4 (54% Et_2O solution, 7.3 mg, 45 µmol) in CH_2Cl_2 (1 mL). The solution was evaporated to dryness, and the oily residue triturated with Et_2O (3 mL). The resulting solid was isolated by filtration, washed with Et_2O (2 × 1 mL), and dried briefly to afford the title compound as a red-brown powder (9.5 mg, 12 µmol, 80%) (Supplementary Figs. 5 and 6).

[(dppe)Ni(µ-pdt)(µ-H)^{57}Fe(^{13}CO)$_3$]BF$_4$ ([1″H]BF$_4$)

This salt was prepared analogously to [**1′**H]BF$_4$, using **1″** as the precursor (Supplementary Figs. 7-9).

[(dppe)Ni(µ-pdt)(µ-D)^{57}Fe(CO)$_3$]BF$_4$ ([1′D]BF$_4$)

A solution of **1′** (10.6 mg, 15 µmol) in CH_2Cl_2 (1 mL) and CD_3OD (0.1 mL) was treated with HBF_4 (54% Et_2O solution, 7.3 mg, 45 µmol) in CD_3OD (0.3 mL). The solution was evaporated to dryness, and the oily residue taken up in CH_2Cl_2 (0.3 mL) and CD_3OD (0.1 mL). Et_2O (3 mL) was added and the mixture cooled to –28°C. The solid that formed was isolated by filtration, washed with Et_2O (2 × 1 mL), and dried briefly to afford the title compound as a red-brown powder (9.8 mg, 12 µmol, 82%) (Supplementary Figs. 10 and 11).

[(dppe)Ni(µ-pdt)(µ-D)^{57}Fe(^{13}CO)$_3$]BF$_4$ ([1″D]BF$_4$)

This salt was prepared analogously to [**1′**D]BF$_4$, using **1″** as the precursor (Supplementary Figs. 12-14).

NRVS Sample Preparation

NRVS samples of [**1′**H]BF_4, [**1′**D]BF_4, [**1″**H]BF_4 and [**1″**D]BF_4 were prepared by suspending a small amount (~3 mg) of each crystalline solid in pentane (~0.2 mL). The mixtures were carefully transferred by Pasteur pipette into separate rectangular NRVS cells. After evaporation, the cells were sealed with Kapton® tape, removed from the glovebox, and stored at 77 K prior to NRVS measurements.

Hydrogenase Sample Preparation

D. vulgaris Miyazaki F Cell Growth and Hydrogenase Protein Isolation

Cells were cultivated using a minimal medium supplemented with $^{57}FeSO_4$, prepared by oxidation of ^{57}Fe (99.7 %, Chemgas, France) with $CuSO_4$. Fermentation was carried out in a 10 L glass bottle (BIOSTAT B plus, Sartorius Stedim Biotech, Germany) under anaerobic conditions, and the [NiFe]-hydrogenase expressed was isolated and purified as described earlier[9].

Conversion to Ni-R1

The as-isolated [NiFe]-hydrogenase was transferred from 25 mM Tris-HCl (pH = 7.4) buffer to100 mM MES (pH = 5.0). The solution was placed in a tube, which was sealed, degassed and then purged with H_2 (1.2 bar) for 8 h. In the case of the sample in D_2O, the buffer was replaced by 100 mM MES (pD = 5.0) in D_2O and the mixture placed under D_2 (1.3 bar) for 8 h. Samples were transferred to a glovebox and loaded into NRVS cells. NRVS experiments were performed on several samples and accumulated. Oxidation states of the samples were confirmed by FT-IR spectroscopy (Supplementary Fig. 31). FT-IR spectra were recorded on a Bruker IFS66v/S FT-IR spectrometer with a 2 cm^{-1} spectral resolution at 293 K. The data collection and baseline correction were carried out using the OPUS software (Bruker). After the NRVS data collection, the reduced sample in H_2O comprised 84% Ni-R1 (ν_{CO} = 1946 cm^{-1}) and 16% Ni-C (at 1962 cm^{-1}), and trace Ni-R2 (ν_{CO} = 1933 cm^{-1}). The sample in D_2O comprised mostly Ni-R1.

NRVS Measurements and Data Analysis

The NRVS spectra were recorded using a published procedure[10] at SPring-8 beamlines BL09XU[11] and BL19LXU[12] in Japan. At BL09XU, a Si(111) double crystal high heat load monochromator (HHLM) produced 14.4 keV radiation with ~1.0 eV resolution, and a high energy resolution monochromator (HRM) [Ge(422)×2Si(975)] subsequently produced 14.4 keV radiation with 0.8 meV resolution. The beam flux was ~1.4×10^9 photons s^{-1}. At BL19LXU, the similar HHLM and HRM provided 14.4 keV radiation with 0.8 meV resolution, but the use of a 25 m undulator afforded a 4.2× higher beam flux[13]. Due to various limitations, the maximum counts/second (cts/s) is currently about 2.6-3 times of that at BL09. During NRVS measurements, the samples were maintained at cryogenic temperature using a liquid He cryostat[2,14]. The real sample temperatures were derived from anti-Stokes / Stokes intensity ratios and were 30-50 K. Delayed nuclear fluorescence and Fe K fluorescence (from internal conversion) were recorded with a 2×2 APD array in either beamline. NRVS spectra were recorded with a step size of 0.28 and 0.27 meV at BL09XU and BL19LXU, respectively (the different step size is due to a different energy scaling factor; both step sizes are 0.295 meV before the energy calibration).

Since the relative strength of NRVS transitions varies dramatically, the scans were divided into segments with very different collection times. In general, 1-3 s per point was used for the range from negative 240 to 400 cm^{-1}, then 5-10 s per point for the Fe−CN and Fe−CO region from 400-620 cm^{-1}, and finally 10-30 s for points in the candidate X−Fe−H bending region (at 620-770 cm^{-1}). To compare the data from different beamlines, we re-scale the BL19 counting time based on its max cts/s vs. the max cts/s at BL09 and create BL09 equivalent scans / BL09 equivalent seconds, *e.g* the 10 s/pt is corresponding to 26 or 30 equivalent s/pt at BL09. Ni-R in H_2O was measured once at BL09XU (10 s × 68) and twice at BL19LXU, (12 + 24) × 2.6 + 68 = 162 equivalent scans, and 660 × 2.6 + 680 = 2396 total equivalent seconds at the possible X−Fe−H bending region (620-770 cm^{-1}). The NiR in D_2O was measured three times at BL09XU and once at BL19LXU, 40+30+36+20×3 = 166 equivalent scans, and total 2780 + 400 × 3 = 3980 equivalent seconds in the X−Fe−H region. The cts/s is 0.6-0.8 at Fe−CO peak (609 cm^{-1}) and 0.10-0.12 at X−Fe−H peak (675 cm^{-1}) while the measured dark background cts are ~0.03 cts/s.

Raw NRVS data were analyzed using PHOENIX software to get the single-phonon ^{57}Fe PVDOS, as previously published[15].

Computational Methods

Model Complex Calculations

The initial coordinates for the DFT calculations on the complexes [(dppe)Ni(μ-pdt)(μ-H/D)^{57}Fe(CO)$_3$]$^+$ ([**1′**H/D]$^+$) were extracted from the dominant Ni-flippamer X-ray structure of [(dppe)Ni(μ-pdt)(μ-H)Fe(CO)$_3$]BF$_4$·3THF[2]. The alternative Fe-flippamer initial structure was obtained using a fine manipulation on the coordinates. The DFT methodology described below is mostly equivalent to our earlier setup on [(dppe)Ni(μ-pdt)^{57}Fe(CO)$_3$]$^{0/+}$ ([**1′**]$^{0/+}$) model complex[8]. For the diamagnetic [**1′**H/D]$^+$ species, the present calculations employed a closed-shell singlet formalism. The structural optimizations and subsequent normal mode analyses were done using GAUSSIAN 09 (ref. [16]), based on the densities exported from single point calculations using JAGUAR 7.9 (ref. [17]) The BP86 (ref. [18,19]) functional and the LACV3P** basis set as implemented in JAGUAR 7.9 were employed. For the first- and second-row elements, LACV3P** implies 6-311G** triple-ζ basis sets including polarization functions. For the Fe and Ni atoms, LACV3P** consists of a triple-ζ basis set for the outermost core and valence orbitals, and the quasirelativistic Los Alamos effective core potential (ECP) for the innermost electrons. The [**1′**H/D]$^+$ species environment was considered using a self-consistent reaction field (SCRF) polarizable continuum model and integral equation formalism (IEF-PCM)[20] as implemented in GAUSSIAN 09 with the IEF-PCM parameters at their default values for water. Based on the normal mode outputs, ^{57}Fe partial vibrational density of states (PVDOS) spectra which complement the NRVS experiment were generated using the Q-SPECTOR program. Q-SPECTOR is an in-house Python tool for analysis of the normal modes calculated using computational chemistry software (here, GAUSSIAN 09), successfully applied earlier[21-24]. To account for the resolution of the present NRVS experiment, the simulated ^{57}Fe PVDOS spectra were broadened by convolution with a FWHM = 12 cm^{-1} Lorentzian. The homogeneous empirical scaling of the calculated frequencies by 108.0/97.9/102.3% in the 0-400/400-1550/1950-2100 cm^{-1} ranges, respectively, was applied to the simulated NRVS (see the main text Fig. 2 and Supplementary Fig. 18) and ν_{CO} IR (Supplementary Fig. 16) spectra, providing essentially unambiguous mapping between the principal calculated and observed features for [**1′**H/D]$^+$ and [**1″**H/D]$^+$. Notably, our attempts to vary the DFT methodology described above using (*i*) alternative functionals (non-hybrid PBE[25,26] and hybrid B3LYP[27-30] (*ii*) larger basis set (adding '+' diffuse functions), (*iii*) two-body D3 dispersion corrections by Grimme *et al.*[31,32] and (*iv*) options on the SCRF procedure did not produce any better correspondence between the calculated ^{57}Fe PVDOS and experimental NRVS data for [**1′**H/D]$^+$.

Enzyme Cluster Model Calculations

Electronic structure calculations on active site cluster models were performed using the ORCA 3.0 quantum chemistry package[33]. Details of the enzyme cluster models have been previously reported by us[6,34]. The initial geometry was prepared from the crystal structure of *DvM* hydrogenase in its reduced form (PDB 1H2R). The metal ions were in their divalent states, as in the Ni(II)Fe(II) Ni-R form. Amino acids of the first and secondary coordination sphere were incorporated in order to ensure that important secondary interactions (H-bonding, steric) with the core region are maintained, which have a direct influence on the electronic structure of the active site. Residue His88 was protonated at the *N*ε position. Constraint geometry optimizations of the models in their respective singlet (S = 0) or triplet (S = 1) states were carried out at the DFT level, using the B3LYP[27-30] hybrid-GGA functional in conjunction with the RIJCOSx[35,36] approximation. In order to ensure high numerical accuracy, tight SCF convergence criteria were used along with increased integration grids (Grid6 and FinalGrid7) in combination with a large COSX grid (IntAccX 5,5,5 and GridX 3,3,4). All calculations employed the def2-TZVPP basis set on both metal atoms and on the atoms in the immediate coordinating sphere, including CN^- and CO, Sγ of cysteines, as well as the substrate atoms (H^-, H^+, H_2). The remaining atoms in the surrounding were treated with the def2-SV(P) basis set. Corresponding auxiliary basis sets for coulomb fitting were provided. The segmented all-electron relativistically contracted (SARC) def2 bases as implemented in ORCA were used[37]. Scalar relativistic effects were incorporated by the all-electron zeroth-order regular approximation (ZORA)[4,38] according to van Wüllen[39]. Dispersion effects were accounted for by the use of Grimme's atom pair-wise van der Waals correction[31,32]. Dielectric effects due to the presence of the enzyme environment were approximated by embedding the clusters in a conductor-like screening model (COSMO)[5,40] with a dielectric constant of ε = 4. The DFT-optimized stationary points were confirmed to be (local) minima by analysis of their harmonic vibrational frequencies, calculated at the same level of theory. Calculated frequencies were scaled by 97.9% (> 400 cm^{-1}), in order to facilitate unequivocal mapping between the principal calculated and experimental NRVS bands for Ni-R and to be consistent with the model complex calculations. Vibrational frequencies and normal mode compositions were subsequently utilized to calculate parameters of NRVS spectra for H and D isotopologues of the cluster models according to the current ORCA implementation. Details on the theory and implementation are reported elsewhere[41,42]. For a given model all relevant hydrogen atoms of the substrate (H_2, H^- and H^+) were included in the isotope substitution. Calculated NRVS

spectra were fit to a Lorentzian, using a consistent linewidth (FWHM, full width at half-height) of 12 cm^{-1}.

Supplementary References

1. Shafaat, H. S., Weber, K., Petrenko, T., Neese, F. & Lubitz, W. Key hydride vibrational modes in [NiFe] hydrogenase model compounds studied by resonance Raman spectroscopy and density functional calculations. *Inorg. Chem.* **51**, 11787−11797 (2012).
2. Barton, B. E., Whaley, C. M., Rauchfuss, T. B. & Gray, D. L. Nickel-iron dithiolato hydrides relevant to the NiFe-hydrogenase active site. *J. Am. Chem. Soc.* **131**, 6942-6943 (2009).
3. Bruschi, M., Tiberti, M., Guerra, A. & De Gioia, L. Disclosure of key stereoelectronic factors for efficient H_2 binding and cleavage in the active site of [NiFe]-hydrogenases. *J. Am. Chem. Soc.* **136**, 1803-1814 (2014).
4. van Lenthe, E., Snijders, J. G. & Baerends, E. J. The zero-order regular approximation for relativistic effects: The effect of spin-orbit coupling in closed shell molecules. *J. Chem. Phys.* **105**, 6505-6516 (1996).
5. Klamt, A. & Schuurmann, G. COSMO - A new approach to dielectric screening in solvents with explicit expressions for the screening energy and its gradient. *J. Chem. Soc.-Perkin Trans. 2*, 799-805 (1993).
6. Krämer, T., Kampa, M., Lubitz, W., van Gastel, M. & Neese, F. Theoretical spectroscopy of the Ni-II intermediate states in the catalytic cycle and the activation of [NiFe] hydrogenases. *ChemBioChem* **14**, 1898-1905 (2013).
7. Schilter, D. *et al.* Mixed-valence nickel-iron dithiolate models of the [NiFe]-hydrogenase active site. *Inorg. Chem.* **51**, 2338-2348 (2012).
8. Schilter, D. *et al.* Synthesis and vibrational spectroscopy of ^{57}Fe-labeled models of [NiFe] hydrogenase: first direct observation of a nickel-iron interaction. *Chem. Commun.* **50**, 13469-13472 (2014).
9. Ogata, H. *et al.* Activation process of [NiFe] hydrogenase elucidated by high-resolution X-ray analyses: Conversion of the ready to the unready state. *Structure* **13**, 1-8 (2005).
10. Wang, H., Alp, E. E., Yoda, Y. & Cramer, S. P. in *Metalloproteins: Methods and Protocols* Vol. 1122 *Methods in Molecular Biology* (Fontecilla-Camps, J.C. & Nicolet, Y., eds) Chapter 9, 125-138 (Springer - Humana Press, NY, 2014).
11. Yoda, Y. *et al.* Nuclear resonant scattering beamline at SPring-8. *Nuc. Instrum. Methods Phys. Res. Sect. A* **467**, 715-718 (2001).
12. Yabashi, M. *et al.* Design of a beamline for the SPring-8 long undulator source 1. *Nuc. Instrum. Methods Phys. Res. Sect. A* **467**, 678-681 (2001).
13. Hara, T. *et al.* The brightest x-ray source: A very long undulator at SPring-8. *Rev. Sci. Instrum.* **73**, 1125-1128 (2002).

14. Wang, H. X., Yoda, Y., Kamali, S., Zhou, Z. H. & Cramer, S. P. Real sample temperature: a critical issue in the experiments of nuclear resonant vibrational spectroscopy on biological samples. *J. Synchrotron Radiat.* **19**, 257-263 (2012).
15. Sturhahn, W. CONUSS and PHOENIX: evaluation of nuclear resonant scattering data. *Hyperfine Interact.* **125**, 149-172 (2000).
16. Frisch, M. J. *et al.* Gaussian 09 Rev A.02. *Gaussian 09, Revision A.02, Gaussian Inc., Wallingford CT* (2009).
17. Chen, C. T., Sette, F., Ma, Y.-J. & Modesti, S. Soft X-ray magnetic circular dichroism of the $L_{2,3}$ edges of nickel. *Phys. Rev. B* **42**, 7262-7265 (1990).
18. Becke, A. D. Density-functional exchange-energy approximation with correct asymptotic-behavior. *Phys. Rev. A* **38**, 3098-3100 (1988).
19. Perdew, J. P. Density-functional approximation for the correlation-energy of the inhomogeneous electron-gas. *Phys. Rev. B* **33**, 8822-8824 (1986).
20. Tomasi, J., Mennucci, B. & Cammi, R. Quantum mechanical continuum solvation models. *Chem. Rev.* **105**, 2999-3093 (2005).
21. Mitra, D. *et al.* Characterization of [4Fe-4S] cluster vibrations and structure in nitrogenase Fe protein at three oxidation levels via combined NRVS, EXAFS, and DFT analyses. *J. Am. Chem. Soc.* **135**, 2530-2543 (2013).
22. Yan, L. F. *et al.* IR-monitored photolysis of CO-inhibited nitrogenase: A major EPR-silent species with coupled terminal CO ligands. *Chem. Eur. J.* **18**, 16349-16357 (2012).
23. Pelmenschikov, V., Guo, Y. S., Wang, H. X., Cramer, S. P. & Case, D. A. Fe-H/D stretching and bending modes in nuclear resonant vibrational, Raman and infrared spectroscopies: Comparisons of density functional theory and experiment. *Faraday Discuss.* **148**, 409-420 (2011).
24. Mitra, D. *et al.* Dynamics of the [4Fe-4S] cluster in *Pyrococcus furiosus* D14C ferredoxin via nuclear resonance vibrational and resonance Raman spectroscopies, force field simulations, and density functional theory calculations. *Biochemistry* **50**, 5220-5235 (2011).
25. Perdew, J. P., Burke, K. & Ernzerhof, M. Generalized gradient approximation made simple. *Phys. Rev. Lett.* **77**, 3865-3868 (1996).
26. Perdew, J. P., Burke, K. & Ernzerhof, M. Generalized gradient approximation made simple (vol 77, pg 3865, 1996). *Phys. Rev. Lett.* **78**, 1396-1396 (1997).
27. Becke, A. D. Density-functional thermochemistry .3. The role of exact exchange. *J. Chem. Phys.* **98**, 5648-5652 (1993).

28. Lee, C. T., Yang, W. T. & Parr, R. G. Development of the Colle-Salvetti correlation-energy formula into a functional of the electron-density. *Phys. Rev. B* **37**, 785-789 (1988).

29. Vosko, S. H., Wilk, L. & Nusair, M. Accurate spin-dependent electron liquid correlation energies for local spin-density calculations - a critical analysis. *Can. J. Phys.* **58**, 1200-1211 (1980).

30. Stephens, P. J., Devlin, F. J., Chabalowski, C. F. & Frisch, M. J. Ab-initio calculation of vibrational absorption and circular-dichroism spectra using density-functional force-fields. *J. Phys. Chem.* **98**, 11623-11627 (1994).

31. Grimme, S., Antony, J., Ehrlich, S. & Krieg, H. A consistent and accurate ab initio parametrization of density functional dispersion correction (DFT-D) for the 94 elements H-Pu. *J. Chem. Phys.* **132**, 154104 (2010).

32. Goerigk, L. & Grimme, S. A thorough benchmark of density functional methods for general main group thermochemistry, kinetics, and noncovalent interactions. *Phys. Chem. Chem. Phys.* **13**, 6670-6688 (2011).

33. Neese, F. The ORCA program system. *WIREs Comput. Mol. Sci.* **2**, 73-78 (2012).

34. Kampa, M., Lubitz, W., van Gastel, M. & Neese, F. Computational study of the electronic structure and magnetic properties of the Ni-C state in NiFe hydrogenases including the second coordination sphere. *J. Biol. Inorg. Chem.* **17**, 1269-1281 (2012).

35. Neese, F., Wennmohs, F., Hansen, A. & Becker, U. Efficient, approximate and parallel Hartree-Fock and hybrid DFT calculations. A 'chain-of-spheres' algorithm for the Hartree-Fock exchange. *Chem. Phys.* **356**, 98-109 (2009).

36. Izsak, R. & Neese, F. An overlap fitted chain of spheres exchange method. *J. Chem. Phys.* **135** (2011).

37. Pantazis, D. A., Chen, X. Y., Landis, C. R. & Neese, F. All-electron scalar relativistic basis sets for third-row transition metal atoms. *J. Chem. Theory Comp.* **4**, 908-919 (2008).

38. van Lenthe, E., Baerends, E. J. & Snijders, J. G. Relativistic regular 2-Component Hamiltonians. *J. Chem. Phys.* **99**, 4597-4610 (1993).

39. van Wüllen, C. Molecular density functional calculations in the regular relativistic approximation: Method, application to coinage metal diatomics, hydrides, fluorides and chlorides, and comparison with first-order relativistic calculations. *J. Chem. Phys* **109**, 392-399 (1998).

40. Sinnecker, S., Rajendran, A., Klamt, A., Diedenhofen, M. & Neese, F. Calculation of solvent shifts on electronic g-tensors with the conductor-like screening model (COSMO) and its self-consistent generalization to real solvents (Direct COSMO-RS). *J. Phys. Chem. A* **110**, 2235-2245 (2006).

41. Petrenko, T., Sturhahn, W. & Neese, F. First-principles calculation of nuclear resonance vibrational spectra. *Hyperfine Interact.* **175**, 165-174 (2007).

42. Petrenko, T. *et al.* Characterization of a genuine iron(V)-nitrido species by nuclear resonant vibrational spectroscopy coupled to density functional calculations. *J. Am. Chem. Soc.* **129**, 11053-11060 (2007).
