# Supplementary figures and images for: Hydride bridge in [NiFe]-hydrogenase observed by nuclear resonance vibrational spectroscopy
Source: Nat Commun. 2015 Aug 10;6:7890. doi: 10.1038/ncomms8890 (PMC4531378; doi:10.1038/ncomms8890)

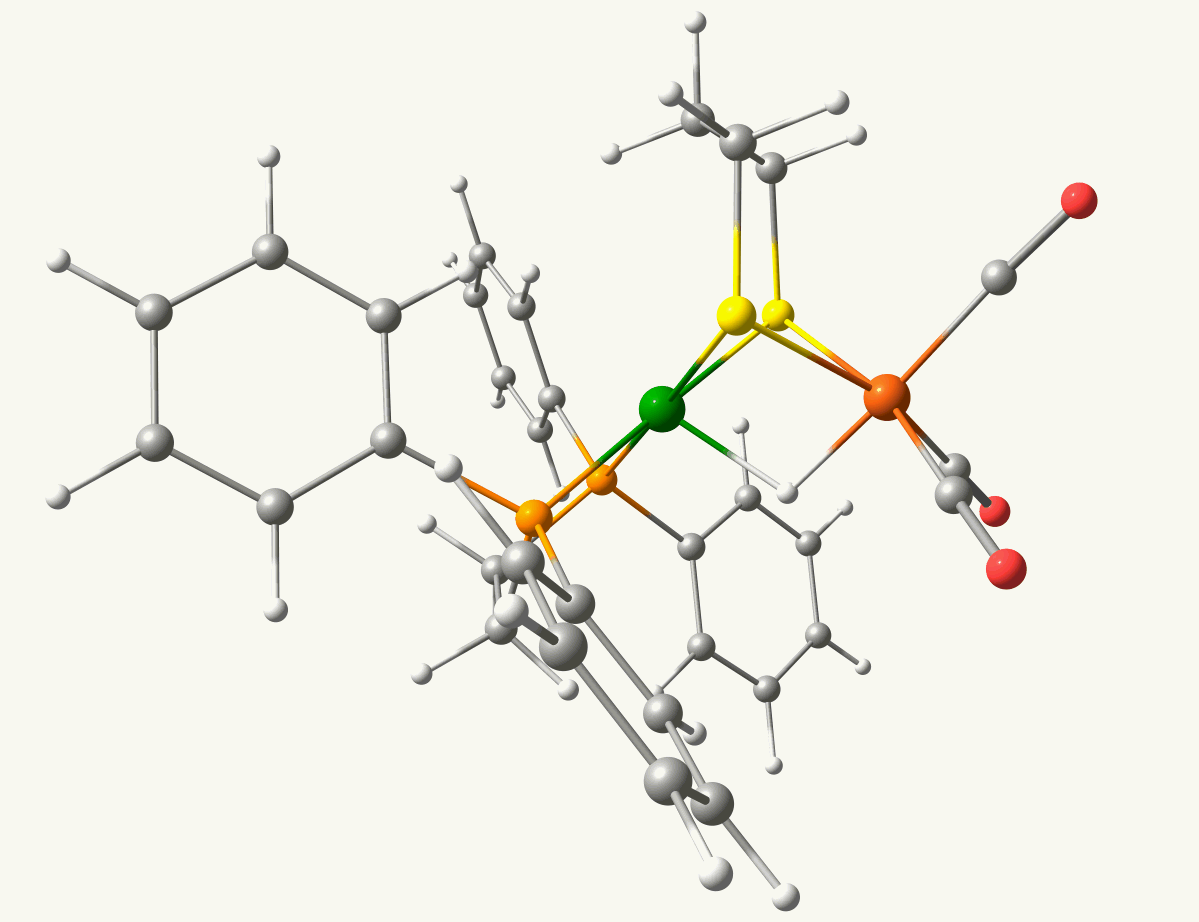

Supplement: Supplementary Movie 1 — Model complex [1'H]+ (H isotopomer), Fe-H stretching mode, v = 1479 cm-1 [file ncomms8890-s2.tif]

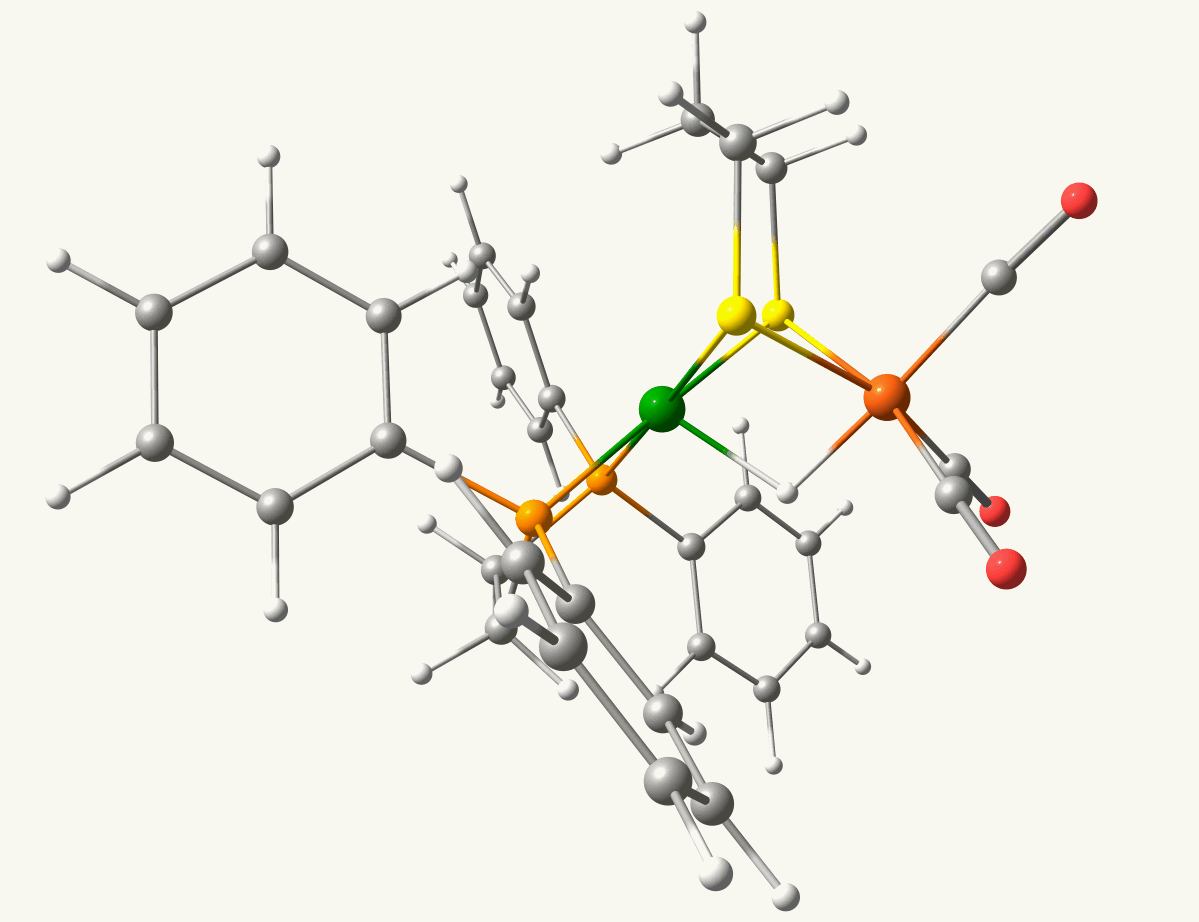

Supplement: Supplementary Movie 2 — Model complex [1'H]+ (H isotopomer), Ni-H stretching mode, v = 1022 cm-1 [file ncomms8890-s3.tif]

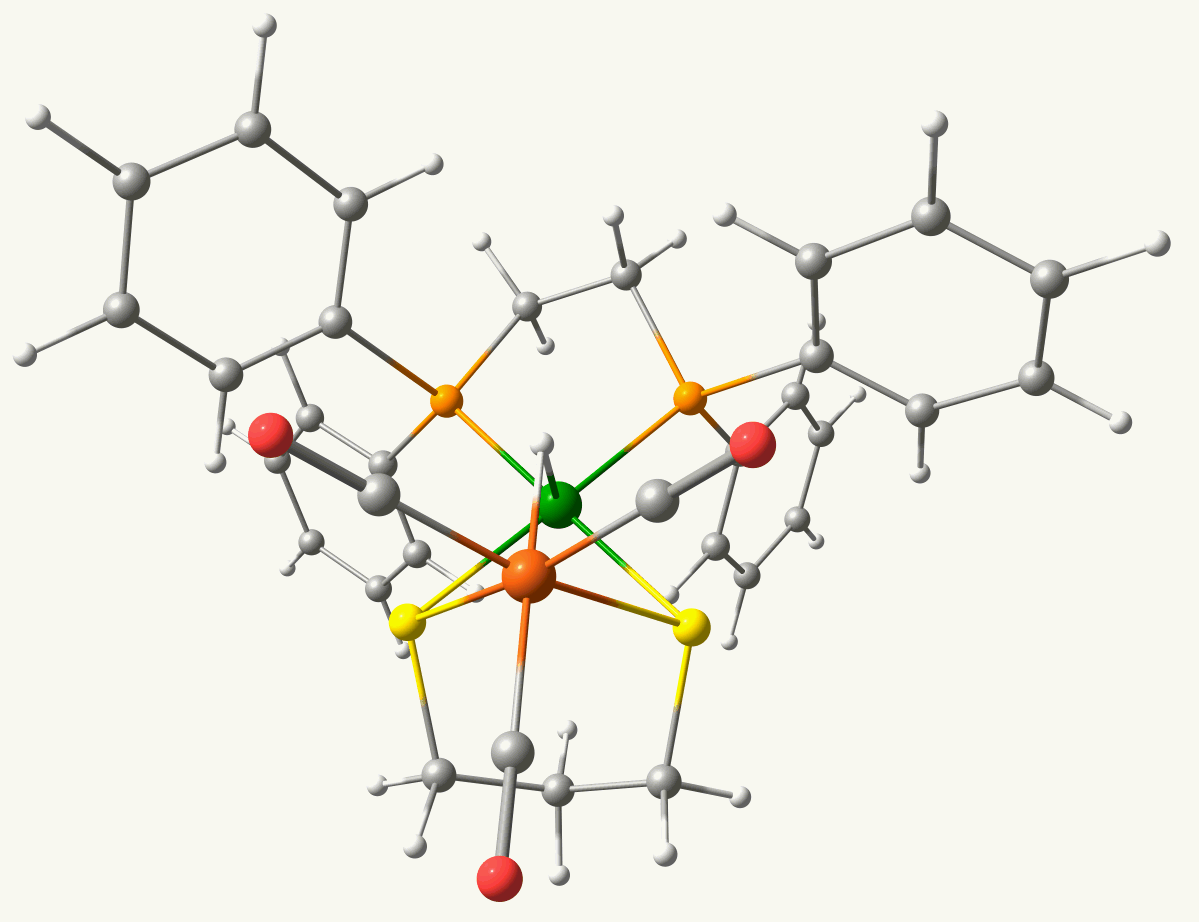

Supplement: Supplementary Movie 3 — Model complex [1'H]+ (H isotopomer), Ni-H-Fe wagging mode, v = 774 cm-1 [file ncomms8890-s4.tif]

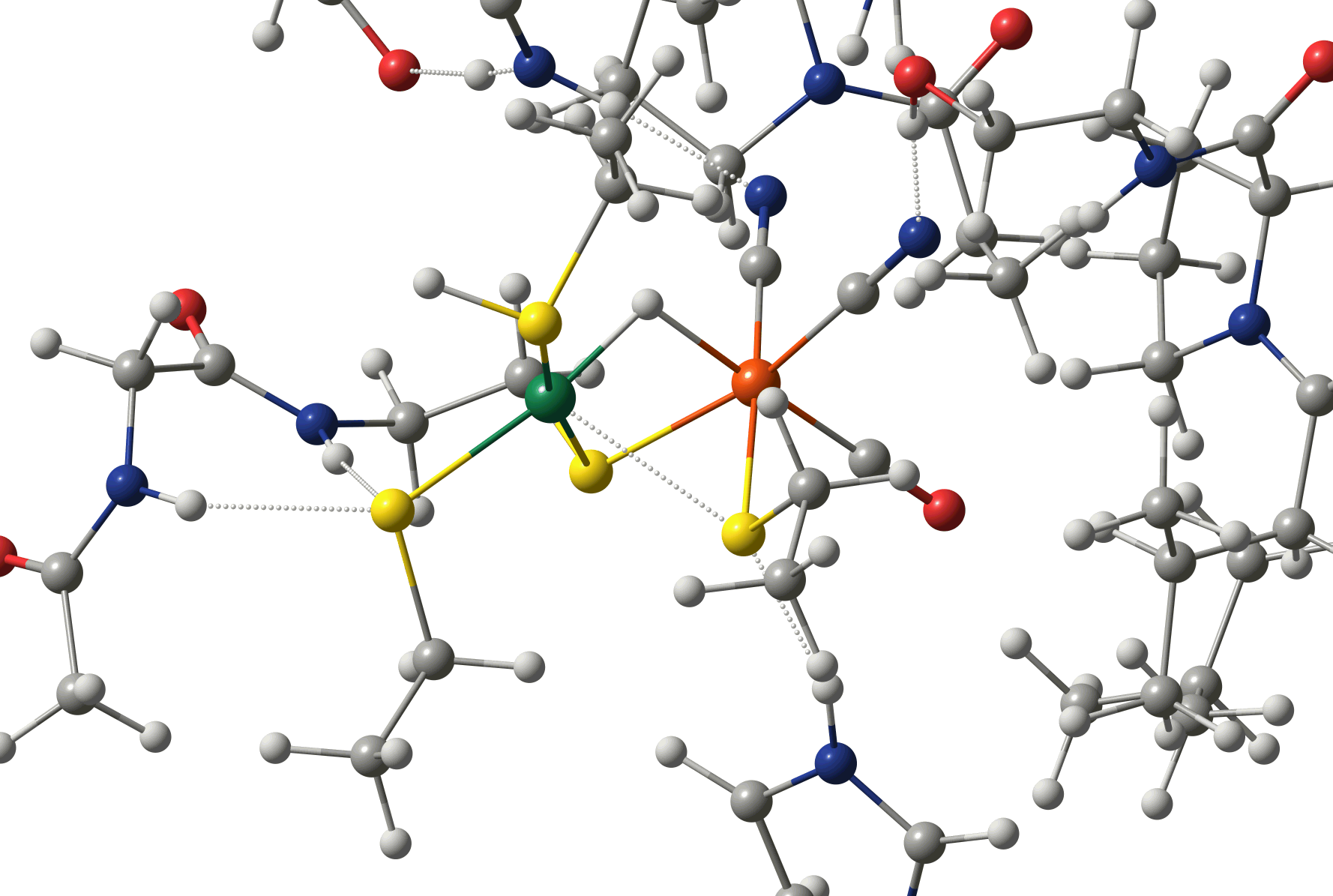

Supplement: Supplementary Movie 4 — Enzyme cluster model V (H isotopomer), Singlet state, v = 411.00 cm-1 [file ncomms8890-s5.tif]

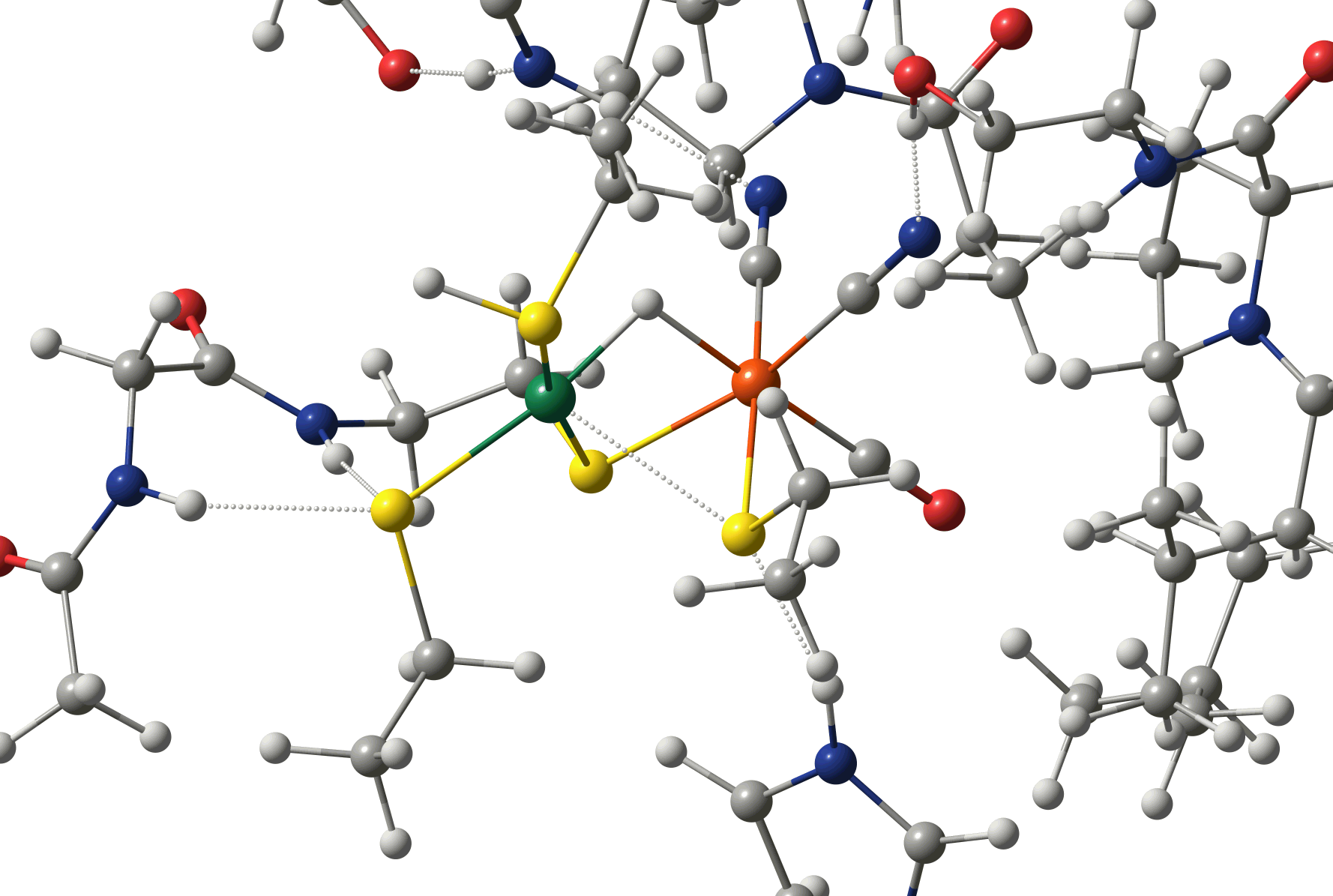

Supplement: Supplementary Movie 5 — Enzyme cluster model V (H isotopomer), Singlet state, v = 439.93 cm-1 [file ncomms8890-s6.tif]

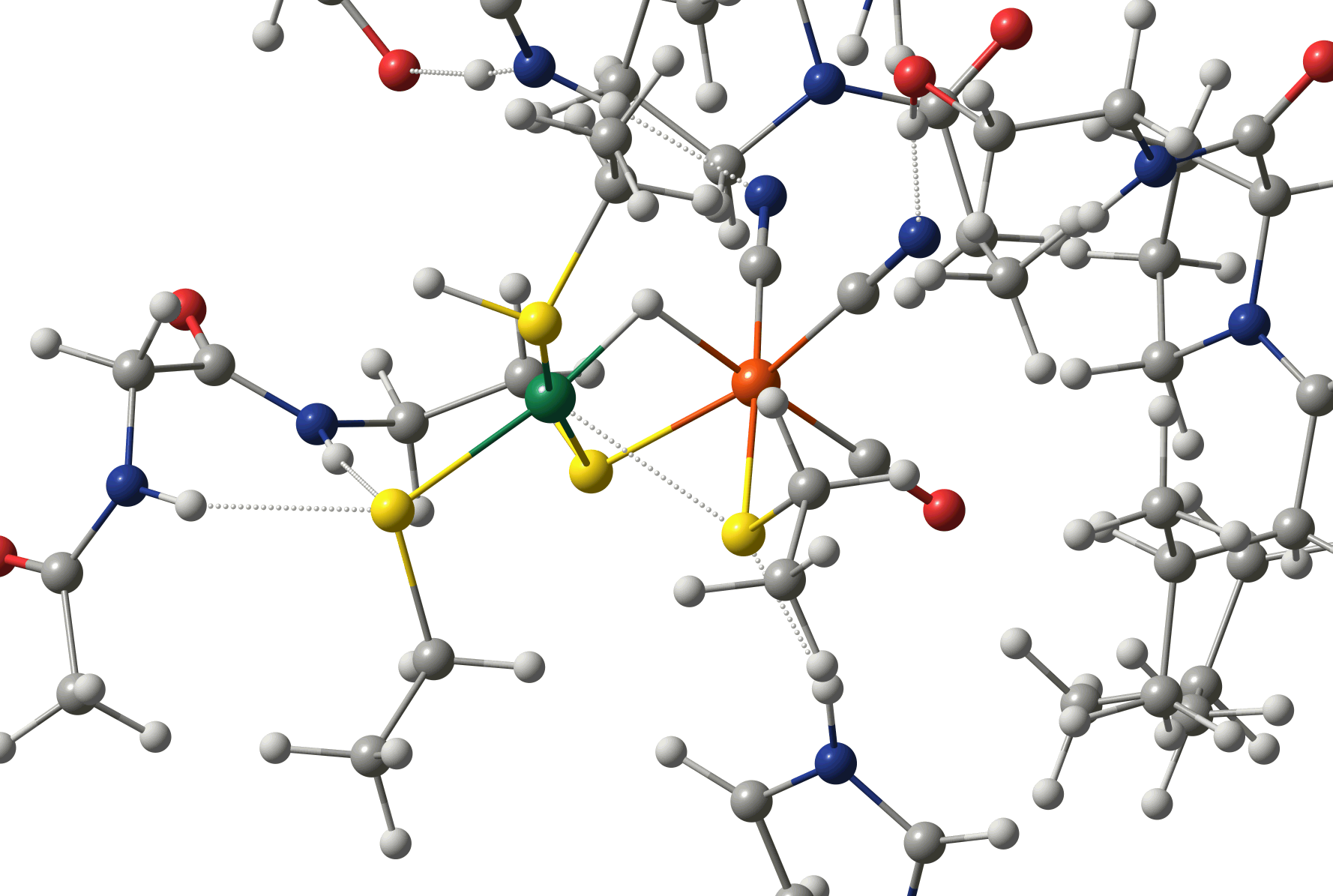

Supplement: Supplementary Movie 6 — Enzyme cluster model V (H isotopomer), Singlet state, v = 460.91 cm-1 [file ncomms8890-s7.tif]

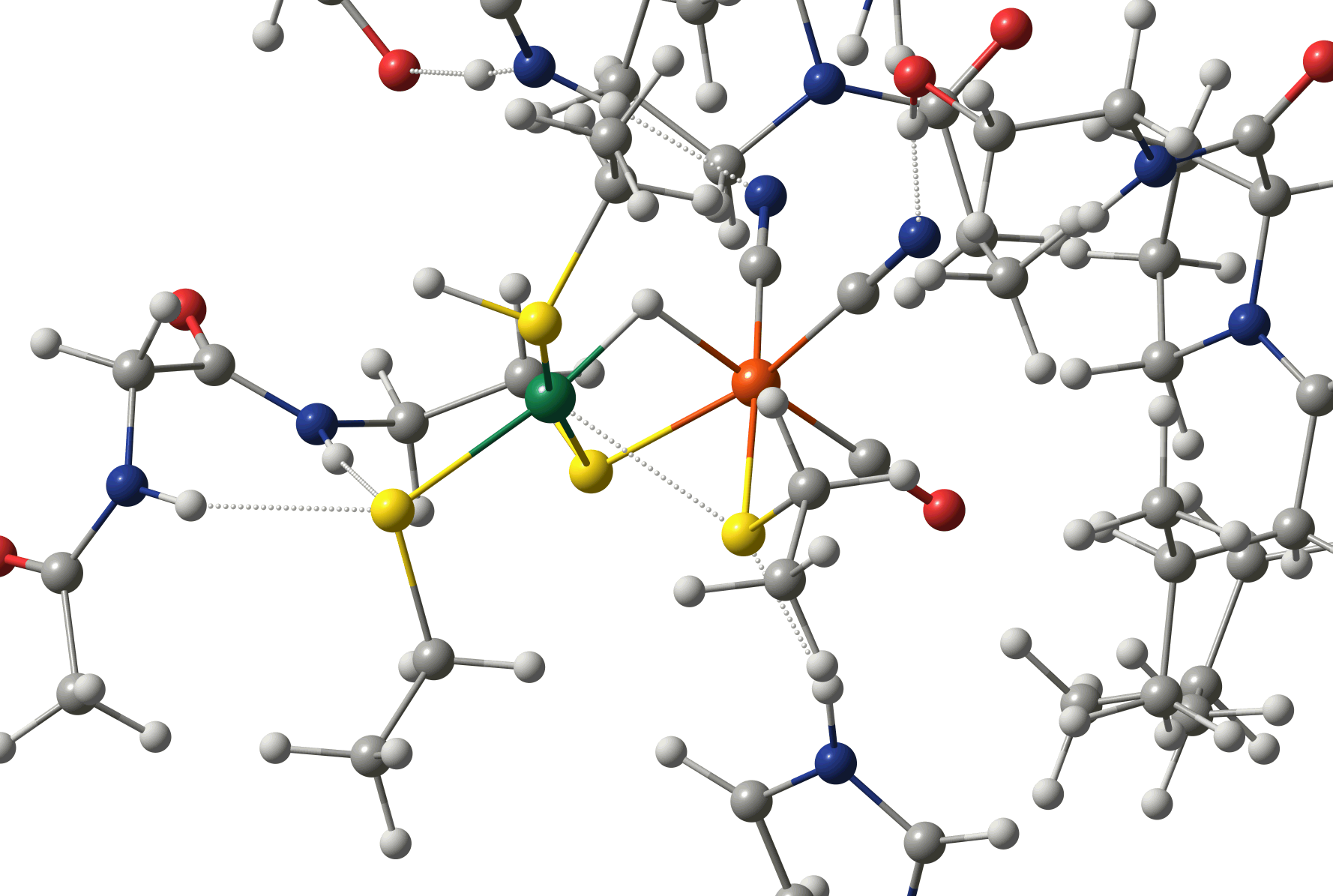

Supplement: Supplementary Movie 7 — Enzyme cluster model V (H isotopomer), Singlet state, v = 504.68 cm-1 [file ncomms8890-s8.tif]

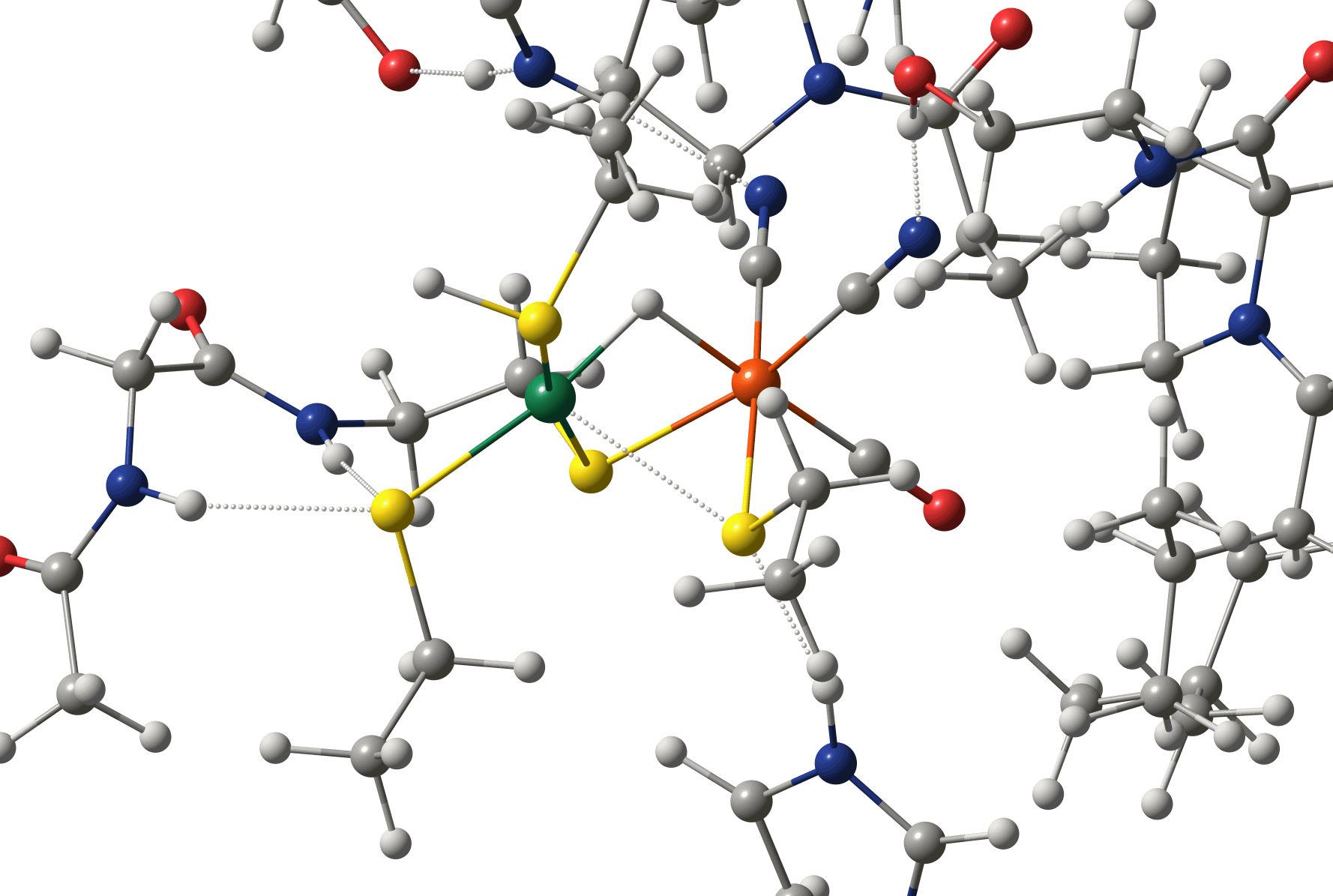

Supplement: Supplementary Movie 8 — Enzyme cluster model V (H isotopomer), Singlet state, v = 543.53 cm-1 [file ncomms8890-s9.tif]

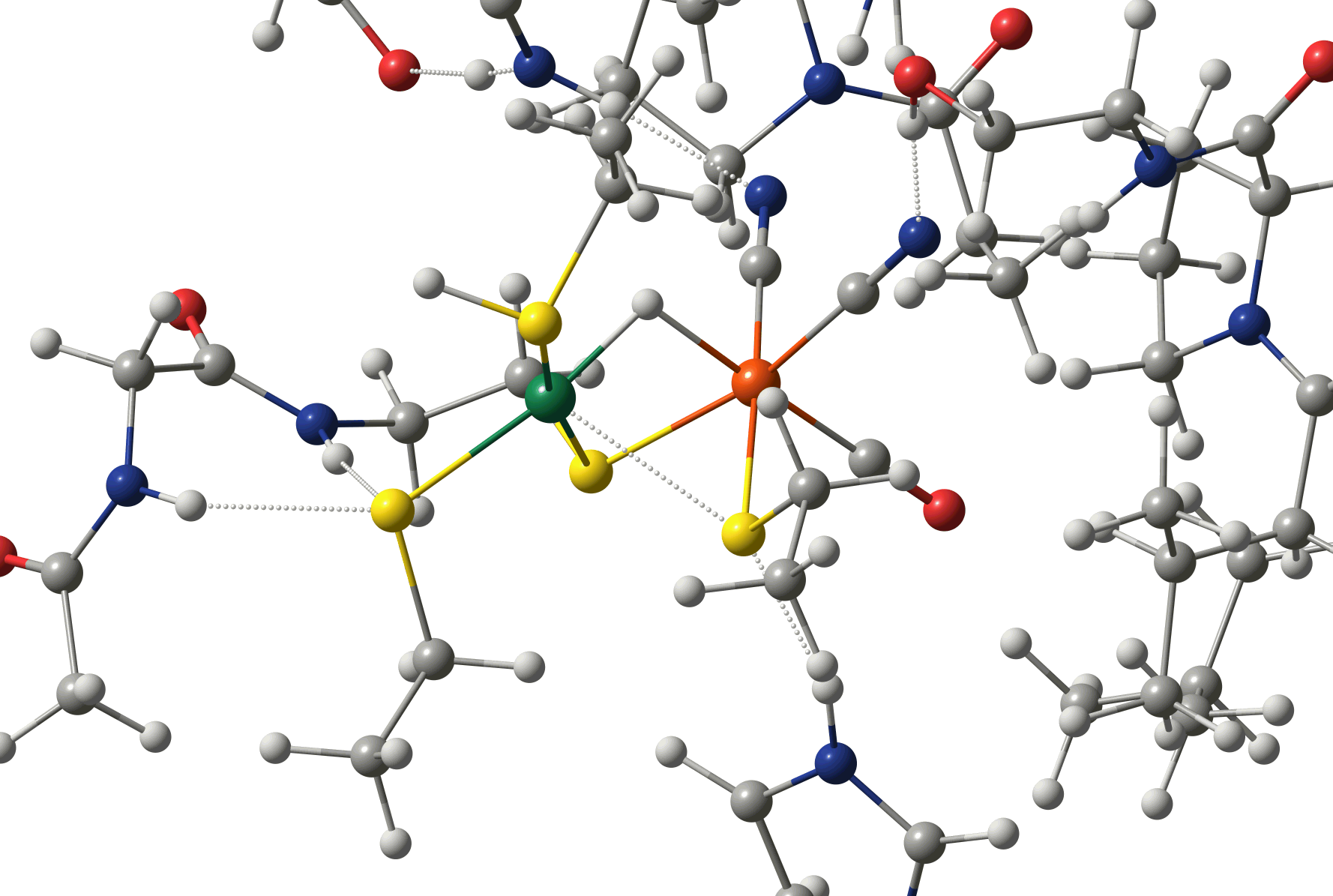

Supplement: Supplementary Movie 9 — Enzyme cluster model V (H isotopomer), Singlet state, v = 588.69 cm-1 [file ncomms8890-s10.tif]

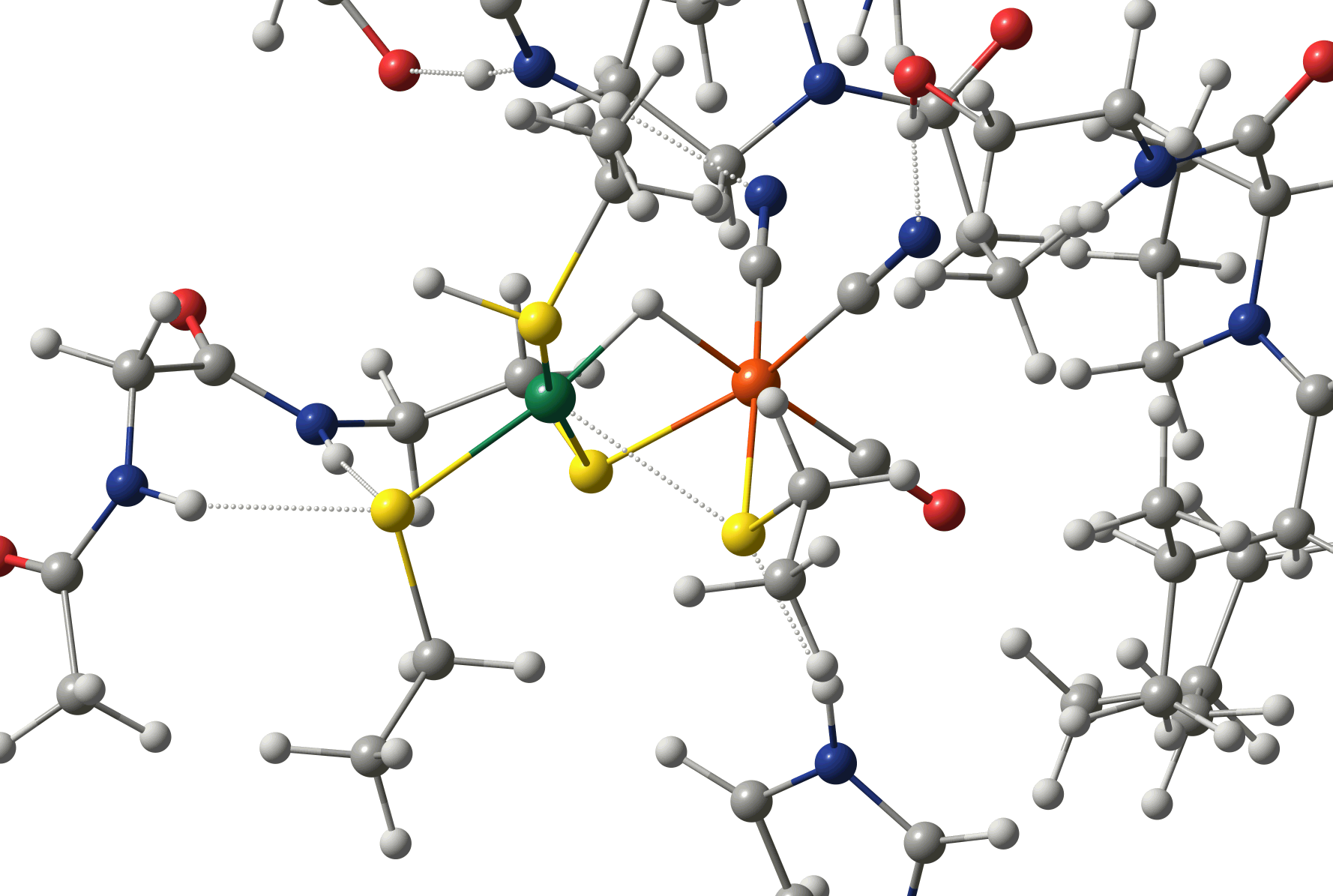

Supplement: Supplementary Movie 10 — Enzyme cluster model V (H isotopomer), Singlet state, v = 613.20 cm-1 [file ncomms8890-s11.tif]

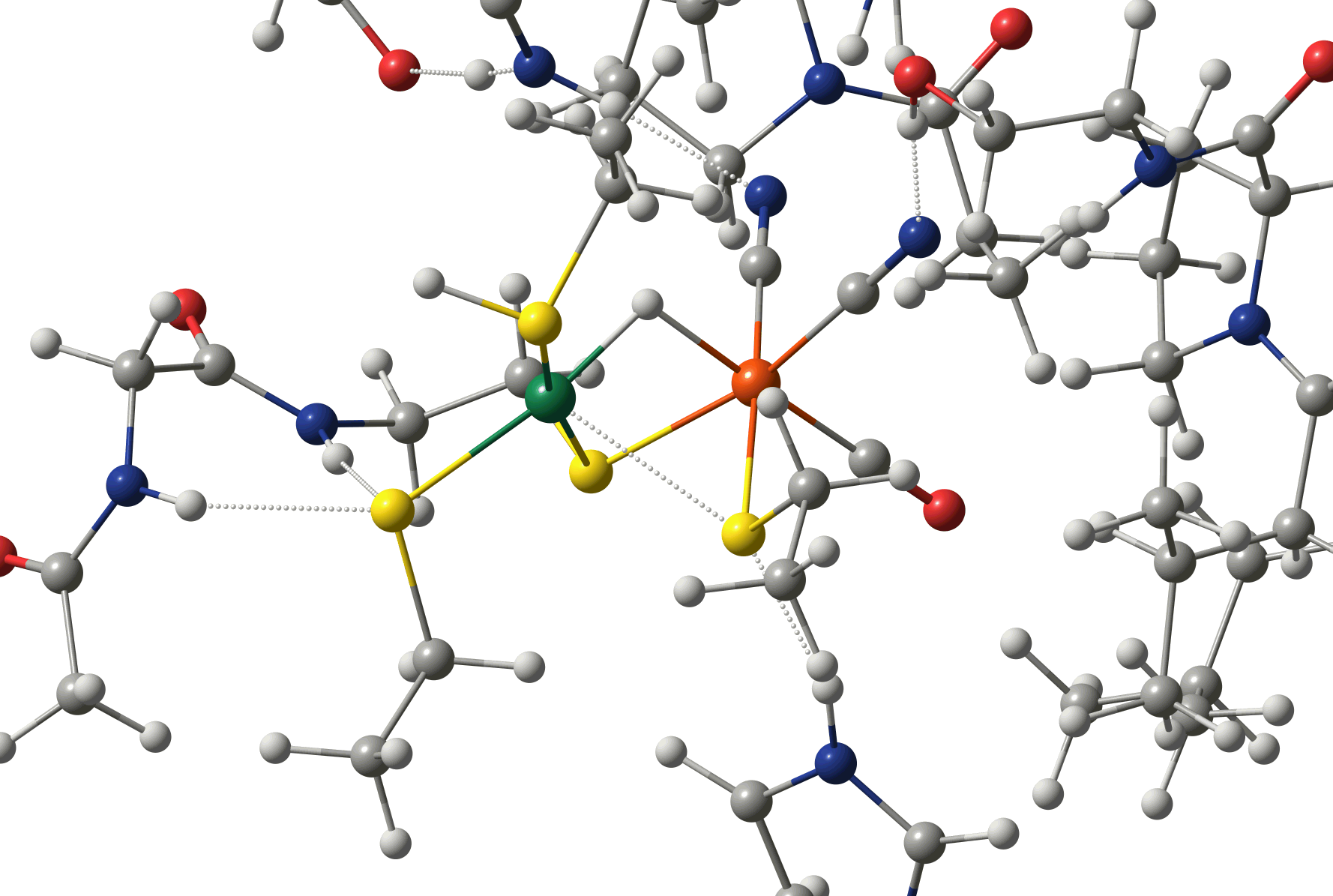

Supplement: Supplementary Movie 11 — Enzyme cluster model V (H isotopomer), Singlet state, v = 726.92 cm-1 [file ncomms8890-s12.tif]

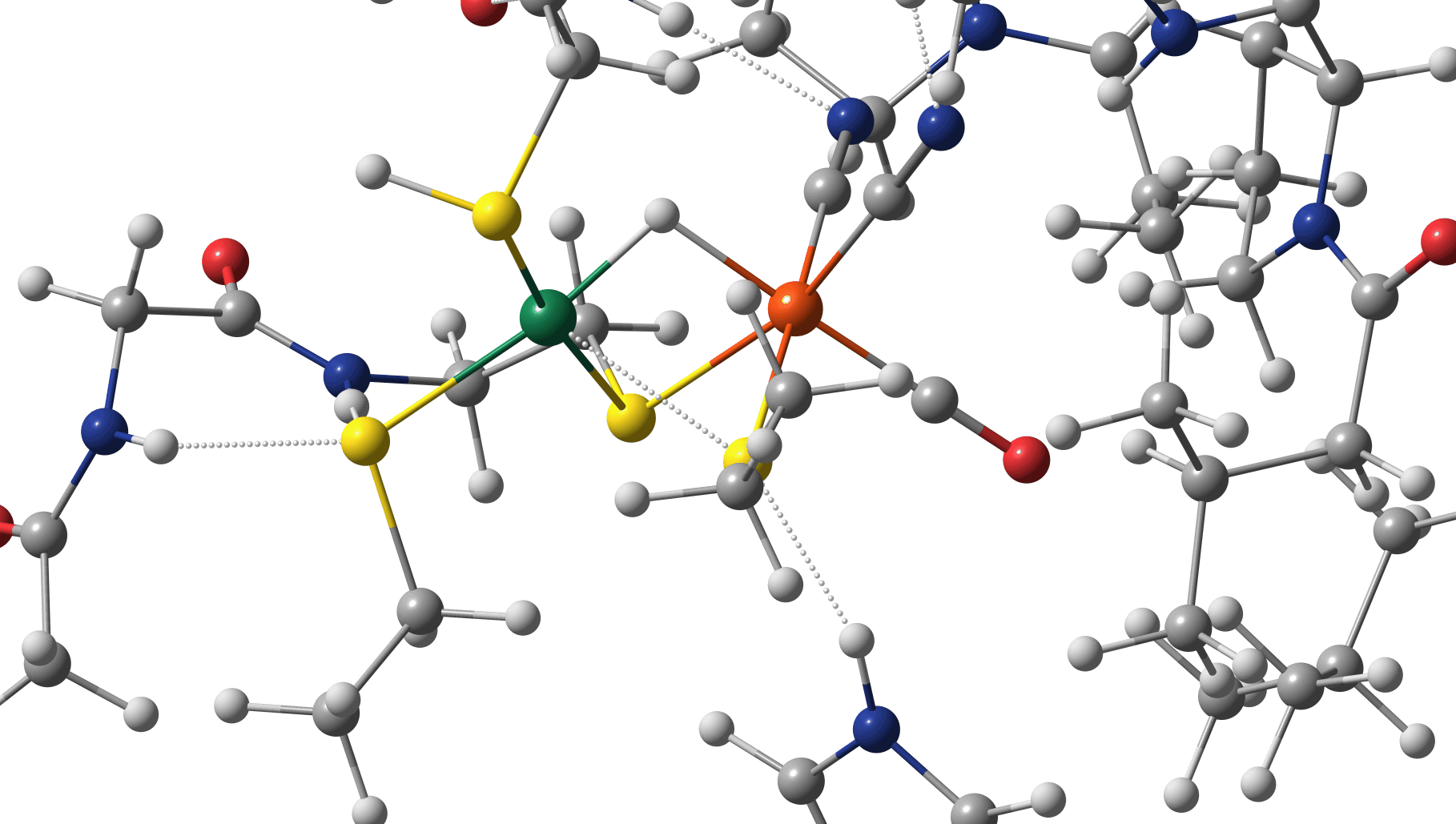

Supplement: Supplementary Movie 12 — Enzyme cluster model V (D isotopomer), Singlet state, v = 408.20 cm-1 [file ncomms8890-s13.tif]

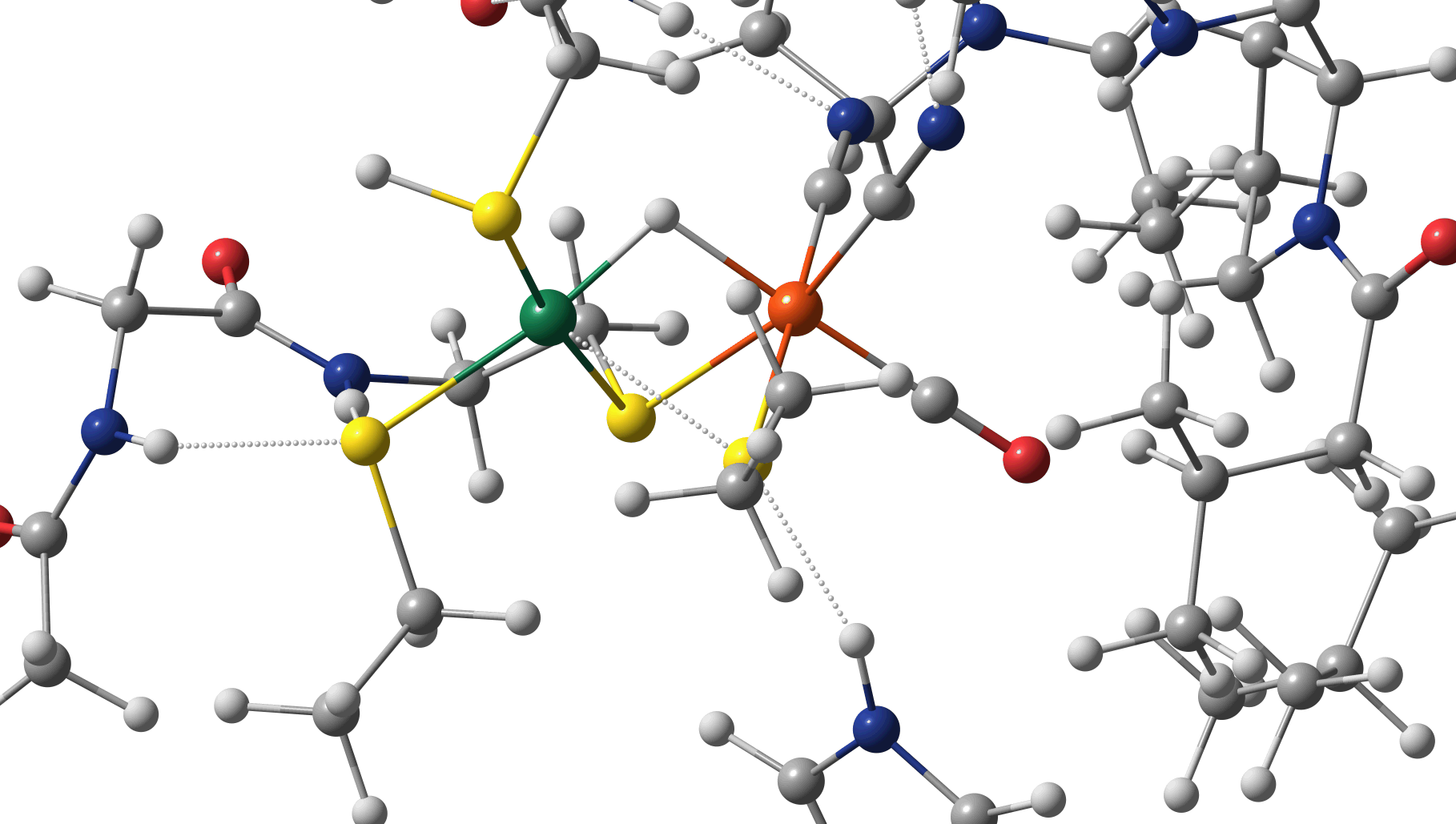

Supplement: Supplementary Movie 13 — Enzyme cluster model V (D isotopomer), Singlet state, v = 432.27 cm-1 [file ncomms8890-s14.tif]

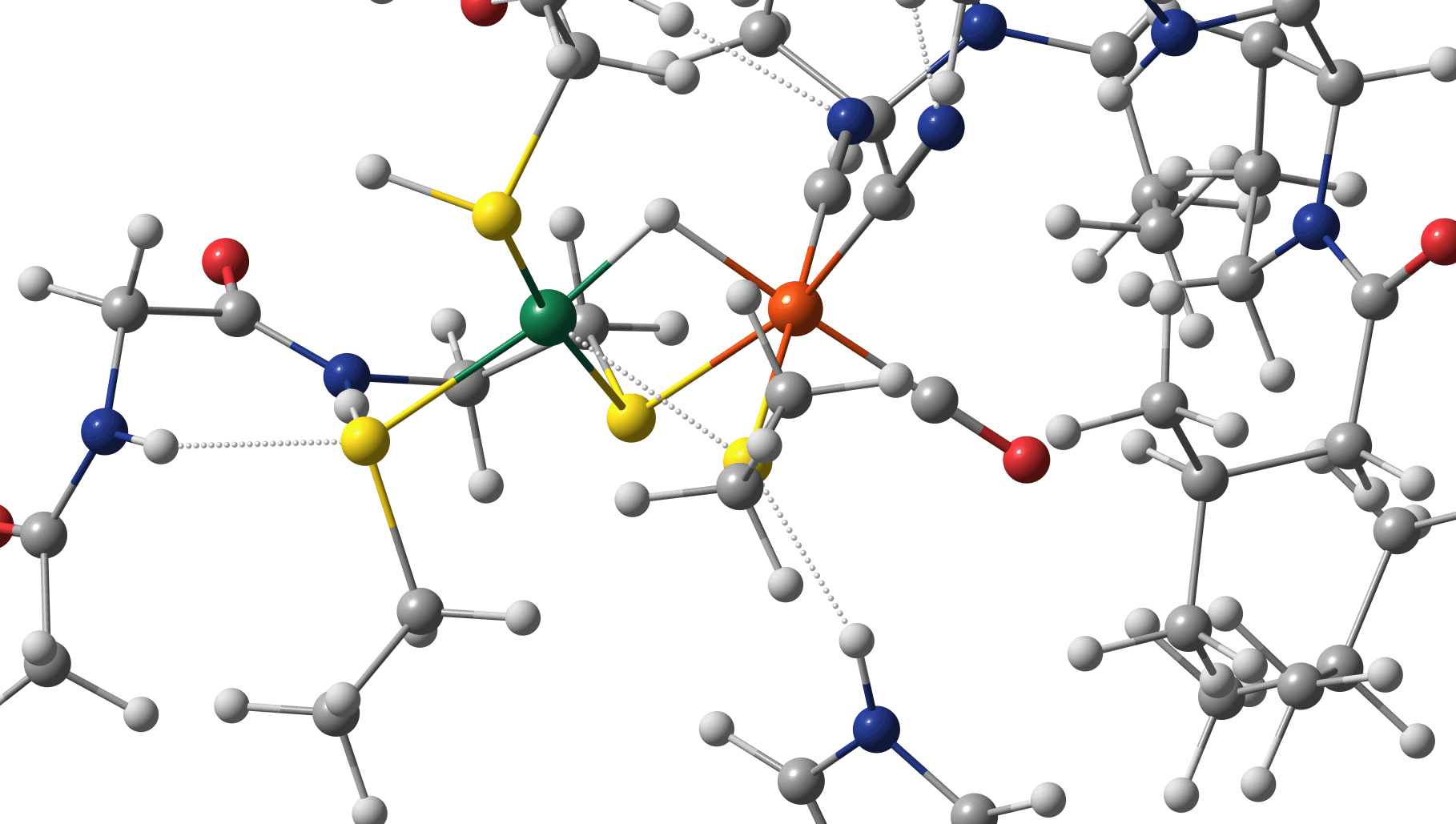

Supplement: Supplementary Movie 14 — Enzyme cluster model V (D isotopomer), Singlet state, v = 438.72 cm-1 [file ncomms8890-s15.tif]

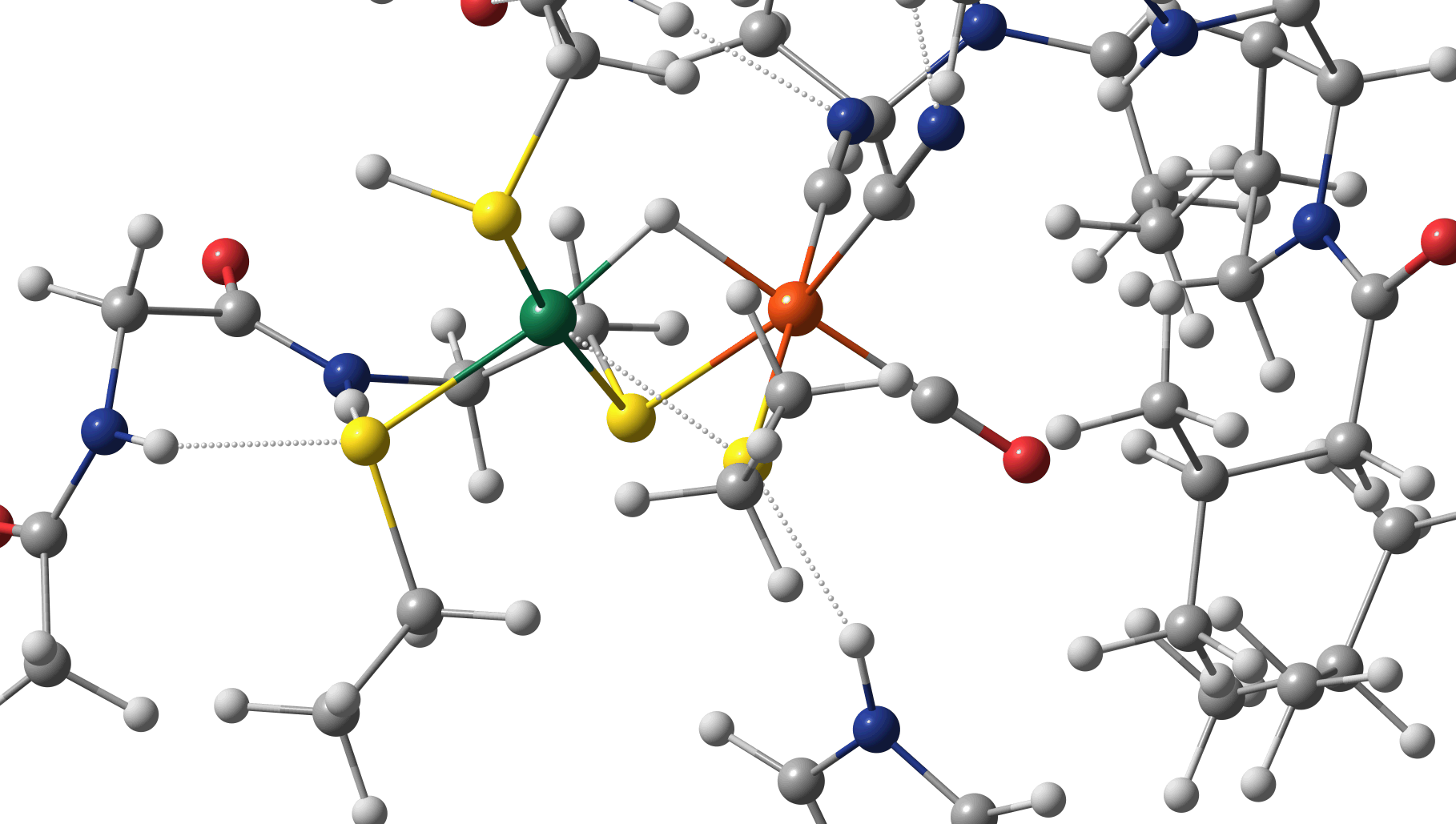

Supplement: Supplementary Movie 15 — Enzyme cluster model V (D isotopomer), Singlet state, v = 458.01 cm-1 [file ncomms8890-s16.tif]

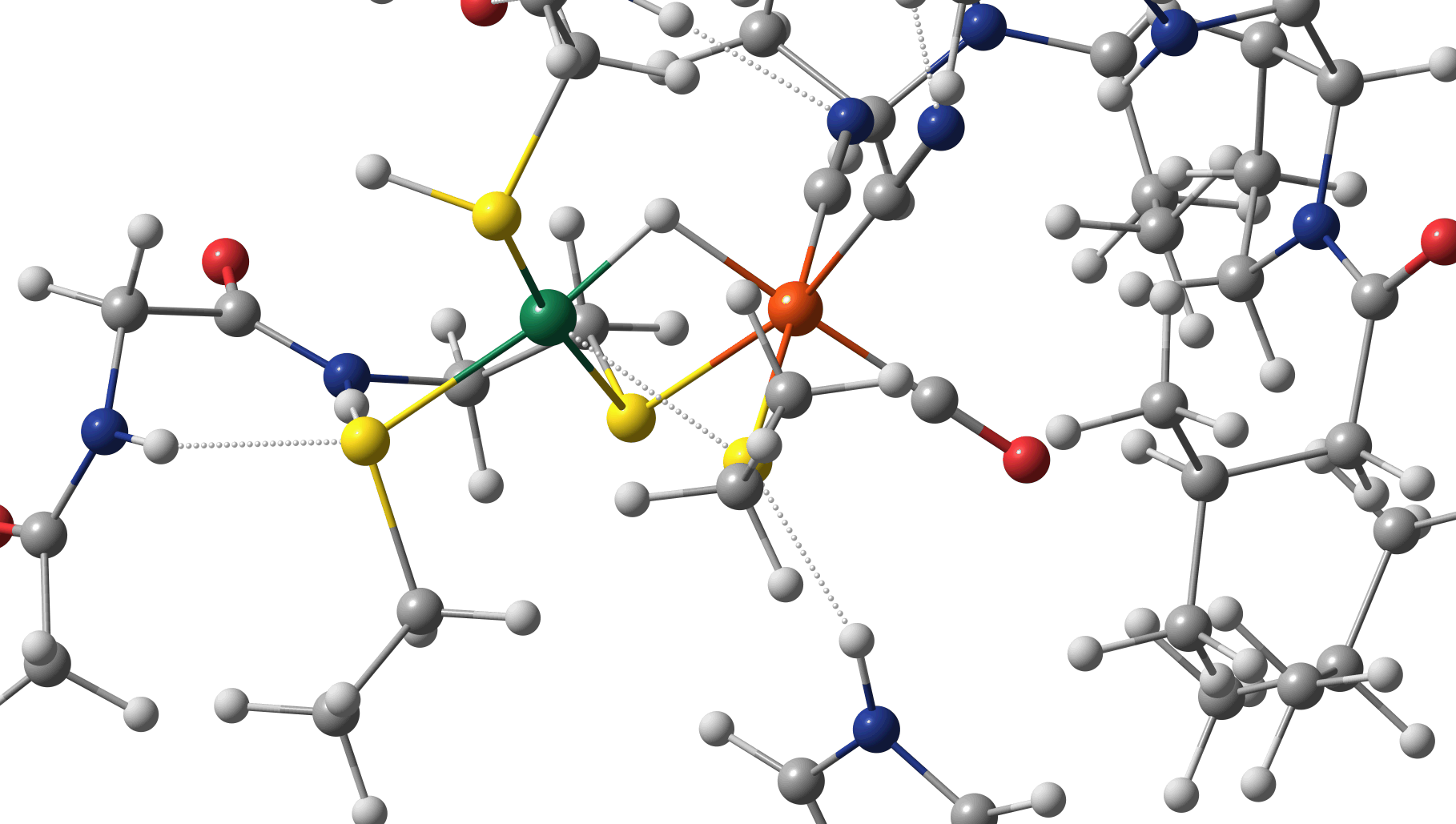

Supplement: Supplementary Movie 16 — Enzyme cluster model V (D isotopomer), Singlet state, v = 504.14 cm-1 [file ncomms8890-s17.tif]

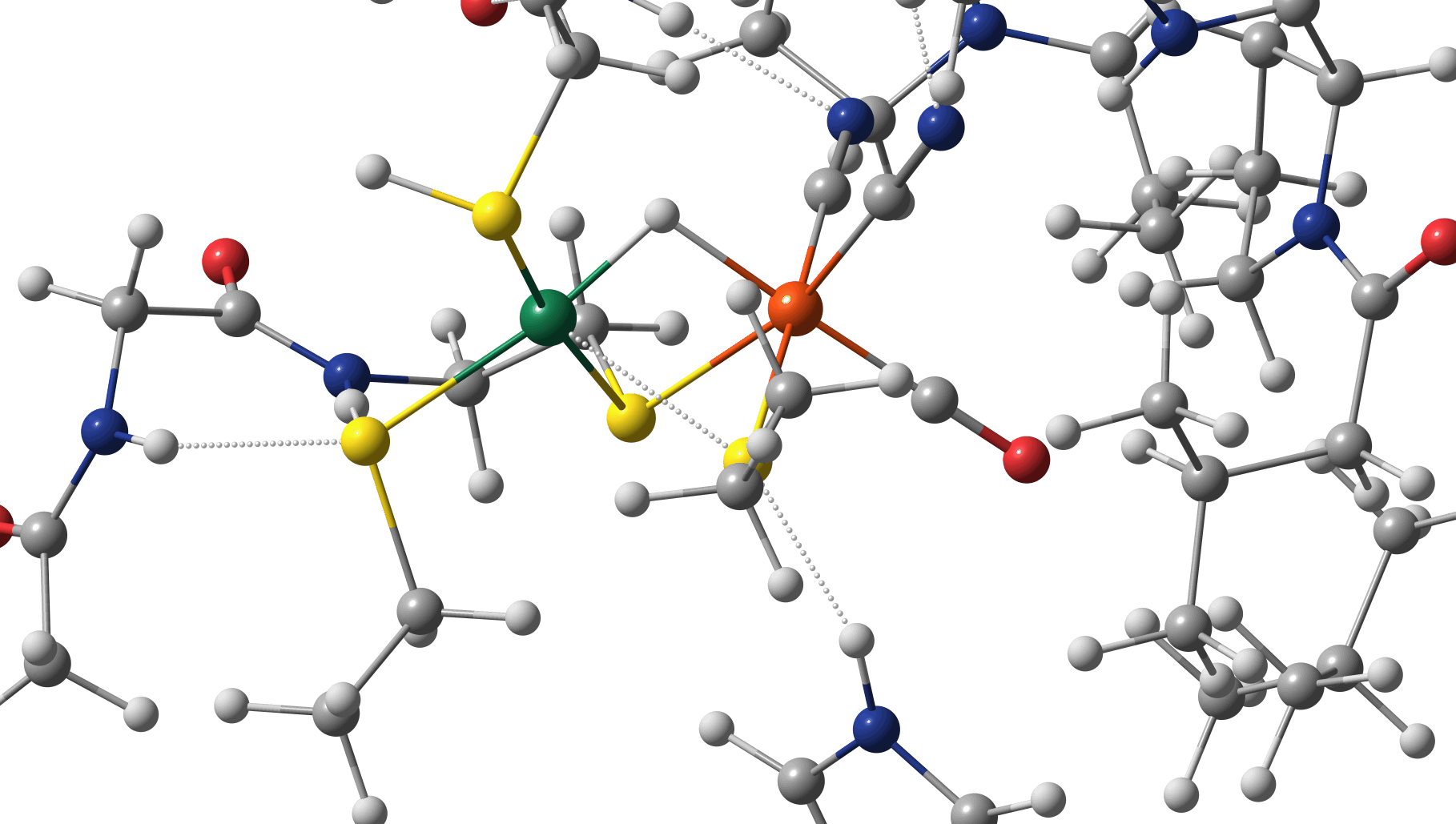

Supplement: Supplementary Movie 17 — Enzyme cluster model V (D isotopomer), Singlet state, v = 522.60 cm-1 [file ncomms8890-s18.tif]

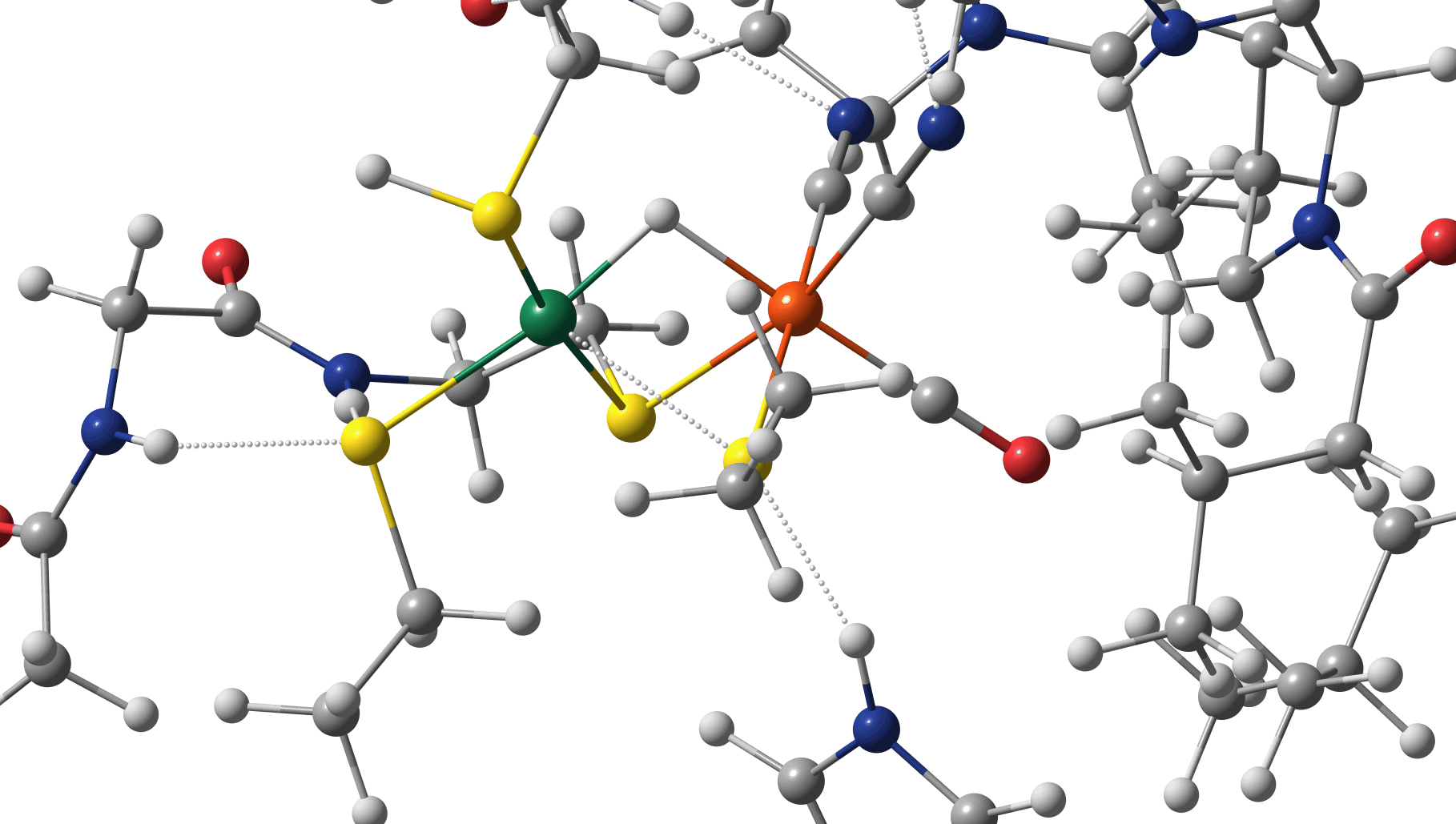

Supplement: Supplementary Movie 18 — Enzyme cluster model V (D isotopomer), Singlet state, v = 544.16 cm-1 [file ncomms8890-s19.tif]

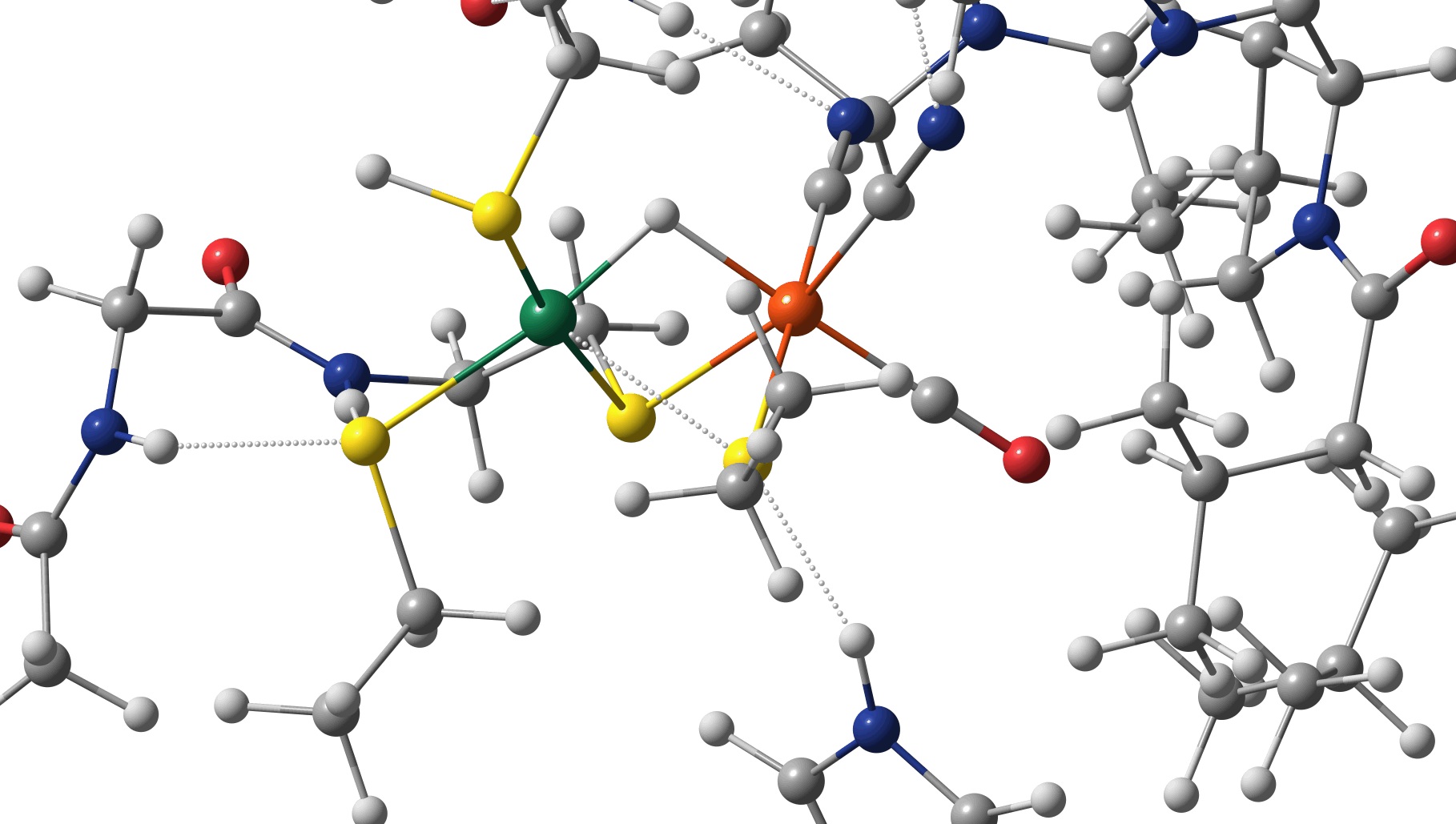

Supplement: Supplementary Movie 19 — Enzyme cluster model V (D isotopomer), Singlet state, v = 600.64 cm-1 [file ncomms8890-s20.tif]

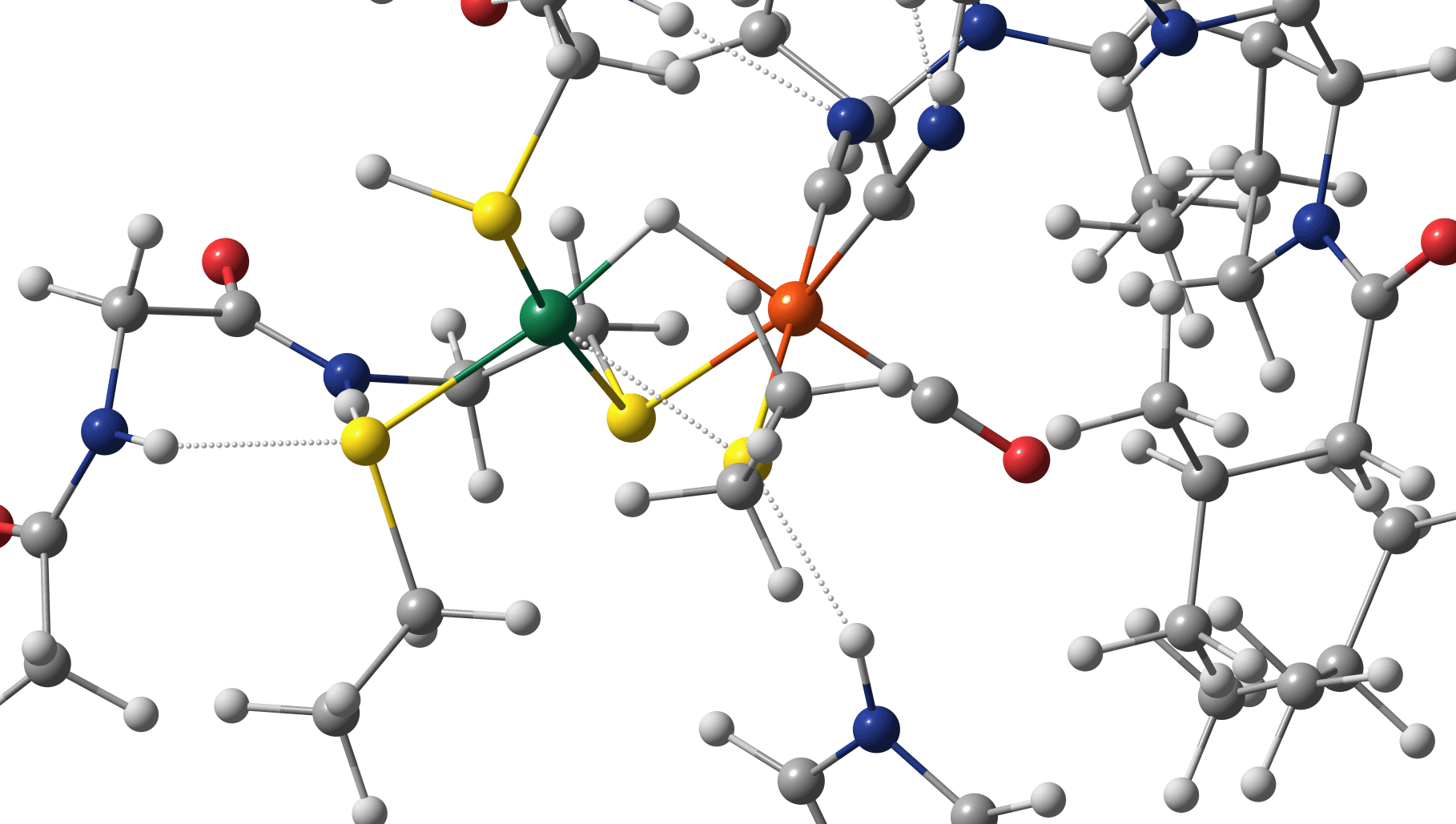

Supplement: Supplementary Movie 20 — Enzyme cluster model V (D isotopomer), Singlet state, v = 613.26 cm-1 [file ncomms8890-s21.tif]

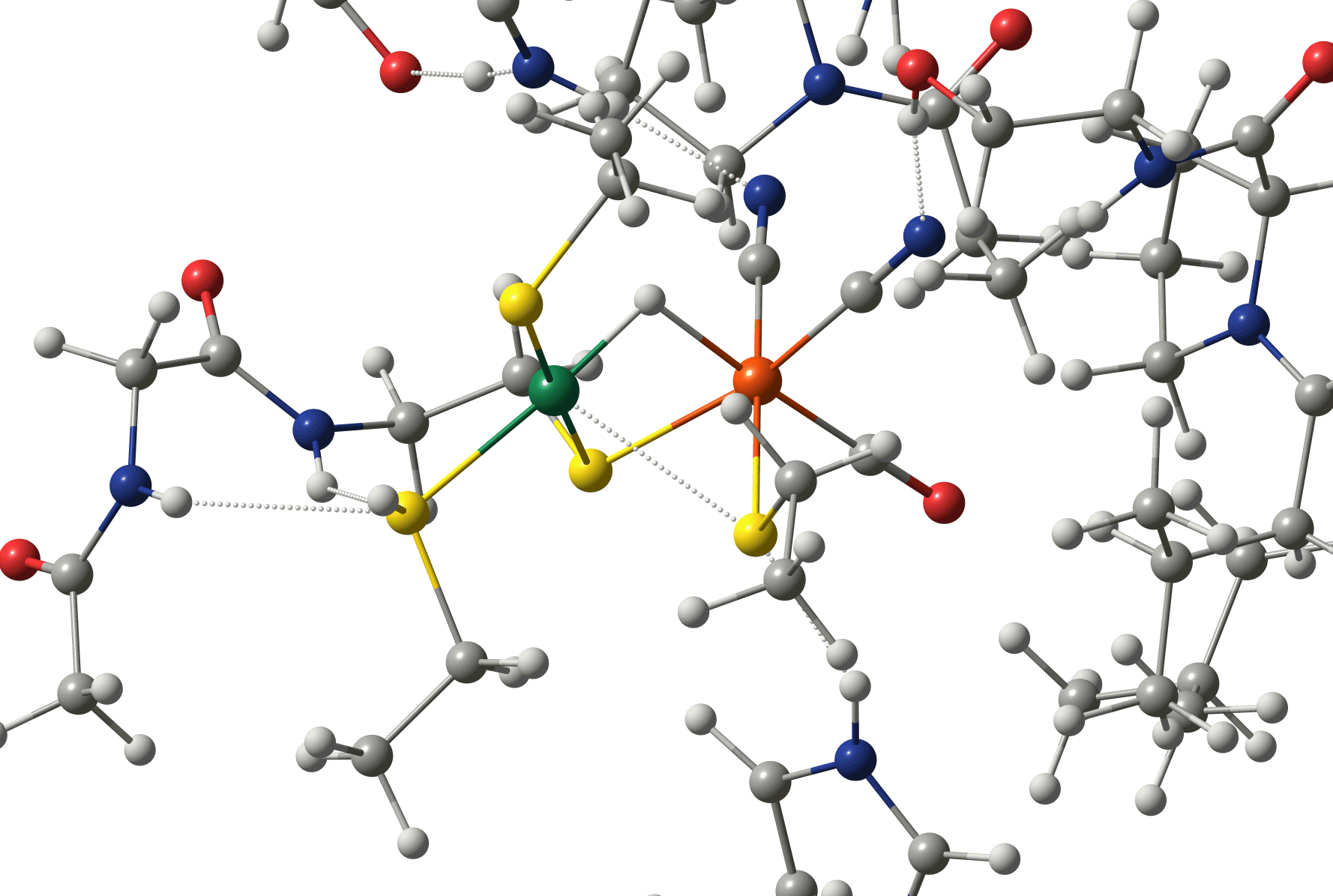

Supplement: Supplementary Movie 21 — Enzyme cluster model VI (H isotopomer), Singlet state, v = 409.49 cm-1 [file ncomms8890-s22.tif]

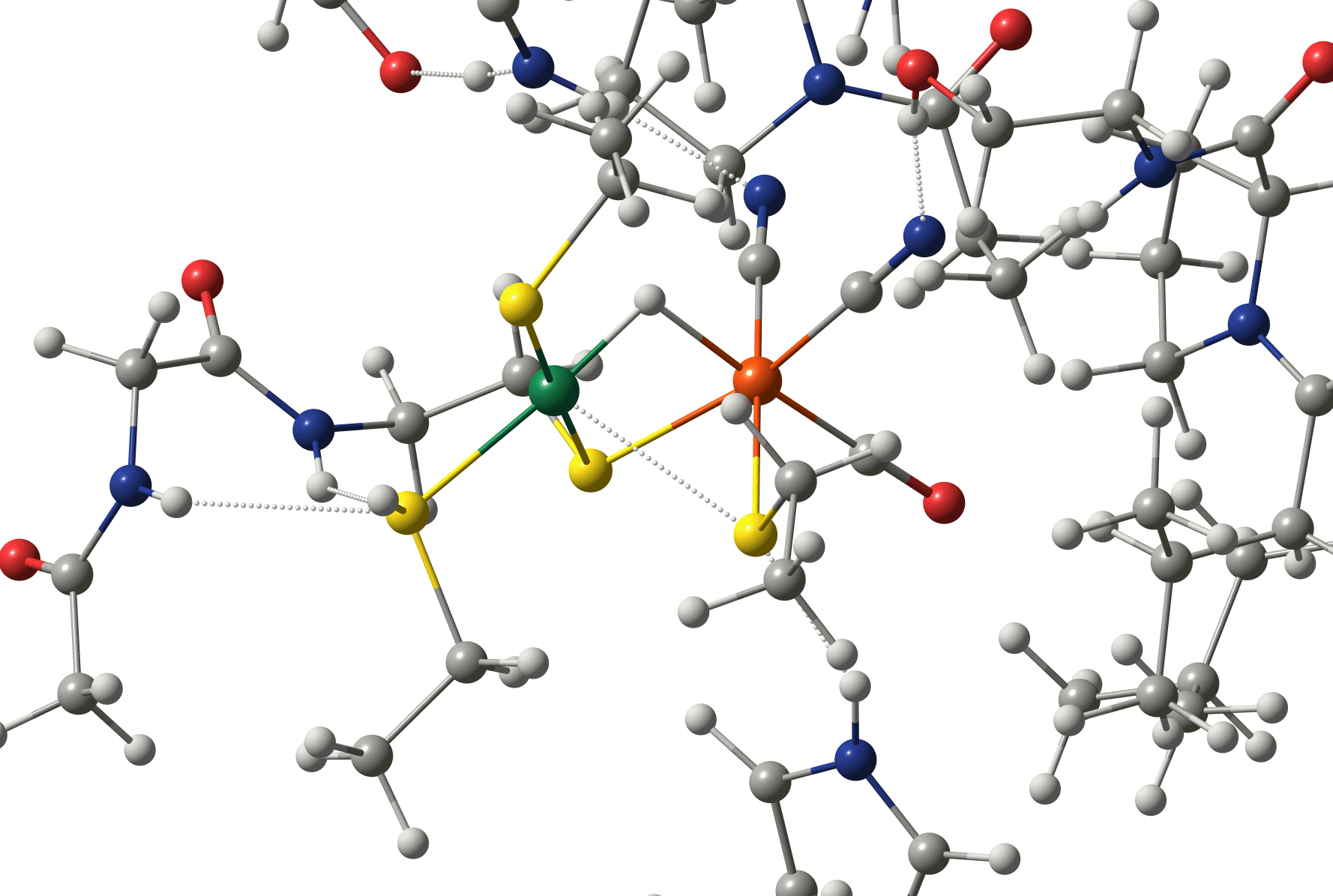

Supplement: Supplementary Movie 22 — Enzyme cluster model VI (H isotopomer), Singlet state, v = 439.68 cm-1 [file ncomms8890-s23.tif]

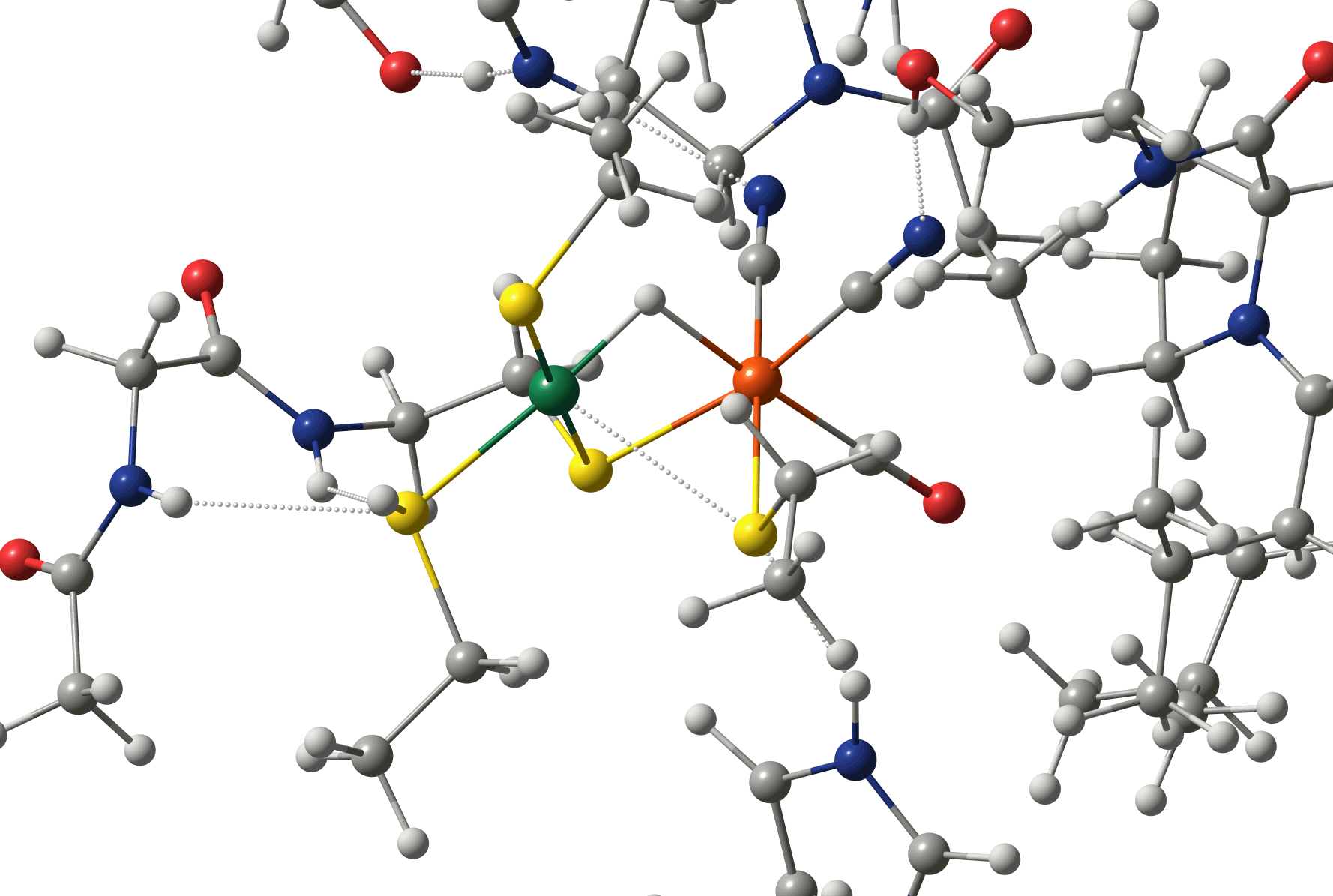

Supplement: Supplementary Movie 23 — Enzyme cluster model VI (H isotopomer), Singlet state, v = 460.61 cm-1 [file ncomms8890-s24.tif]

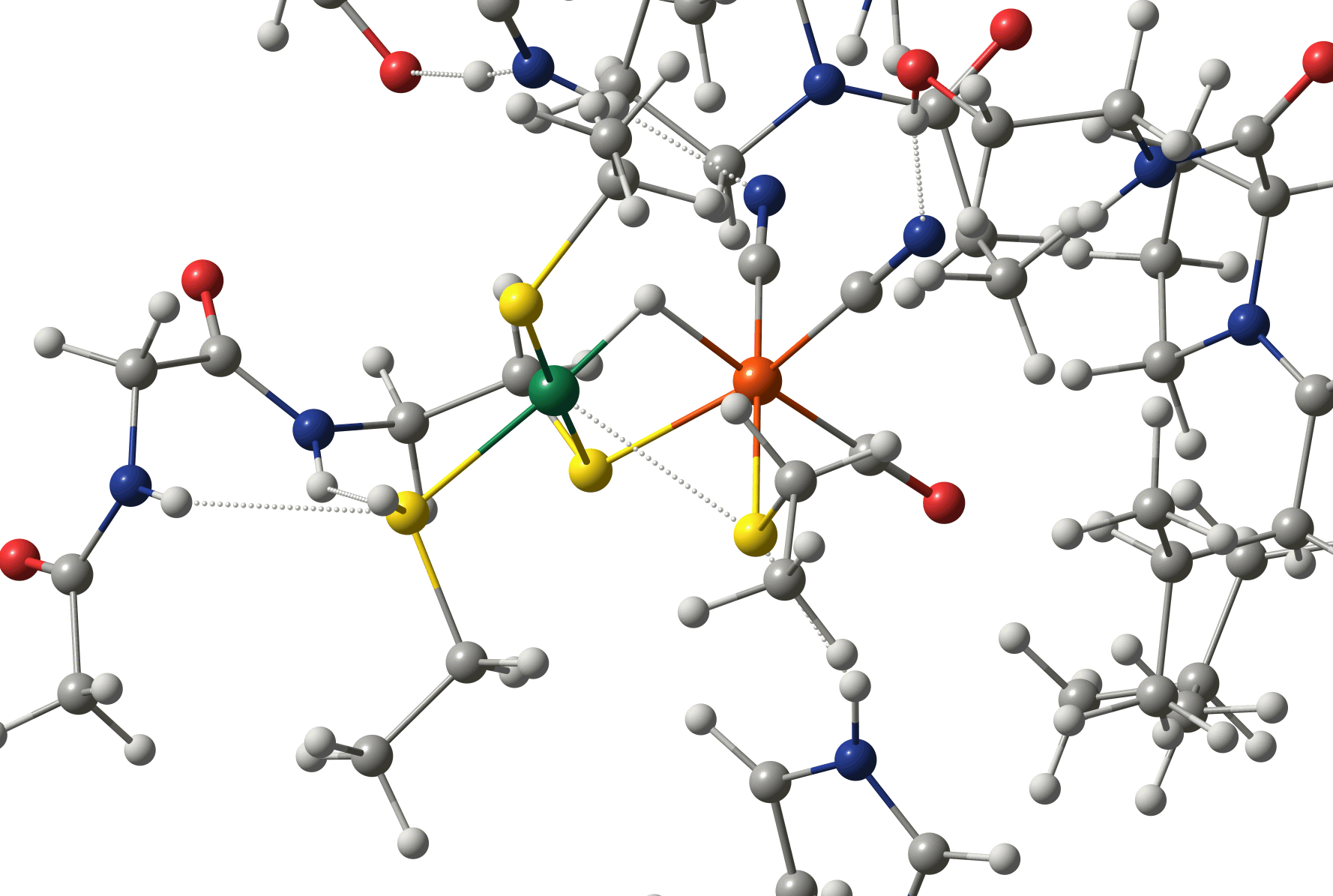

Supplement: Supplementary Movie 24 — Enzyme cluster model VI (H isotopomer), Singlet state, v = 502.78 cm-1 [file ncomms8890-s25.tif]

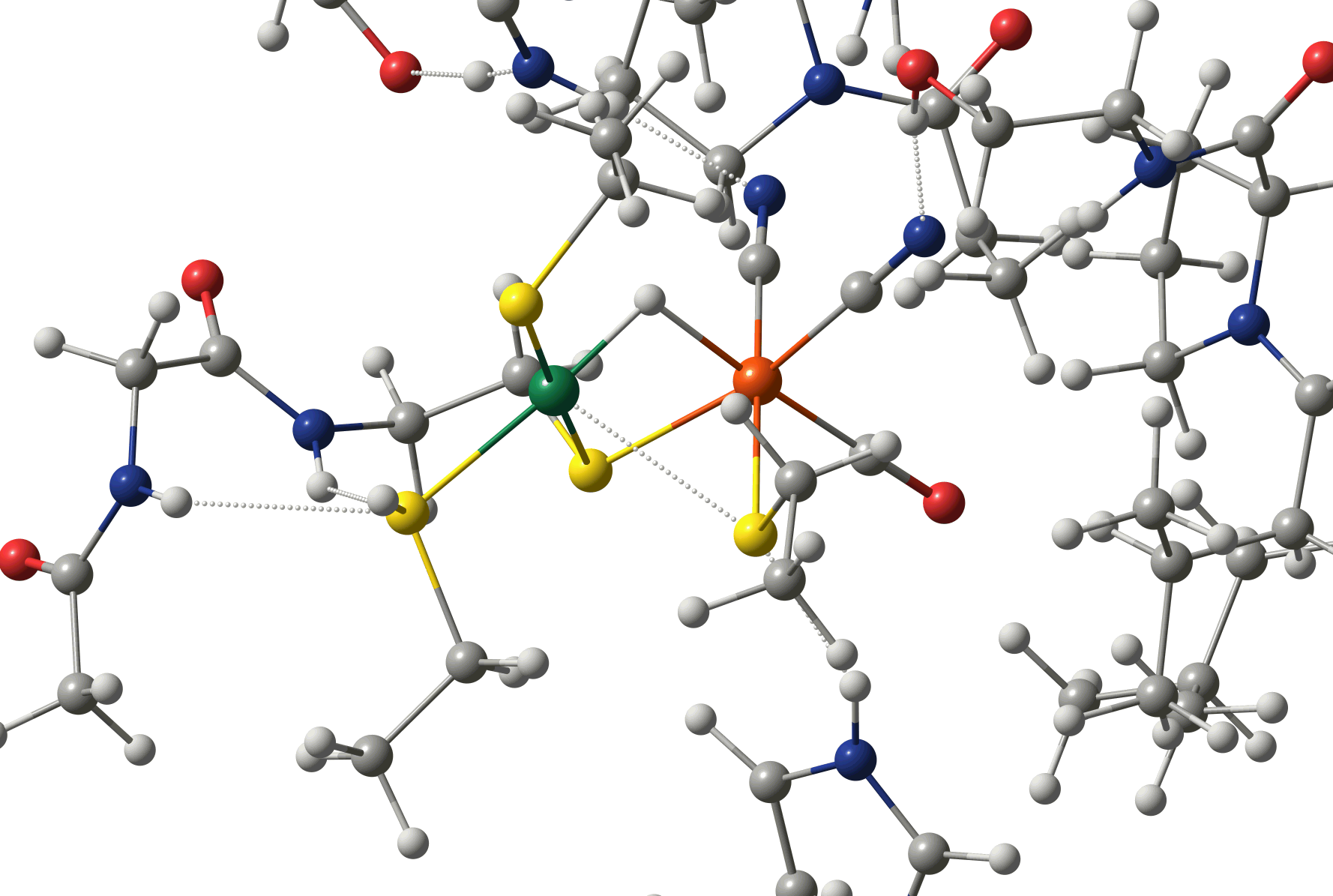

Supplement: Supplementary Movie 25 — Enzyme cluster model VI (H isotopomer), Singlet state, v = 545.14 cm-1 [file ncomms8890-s26.tif]

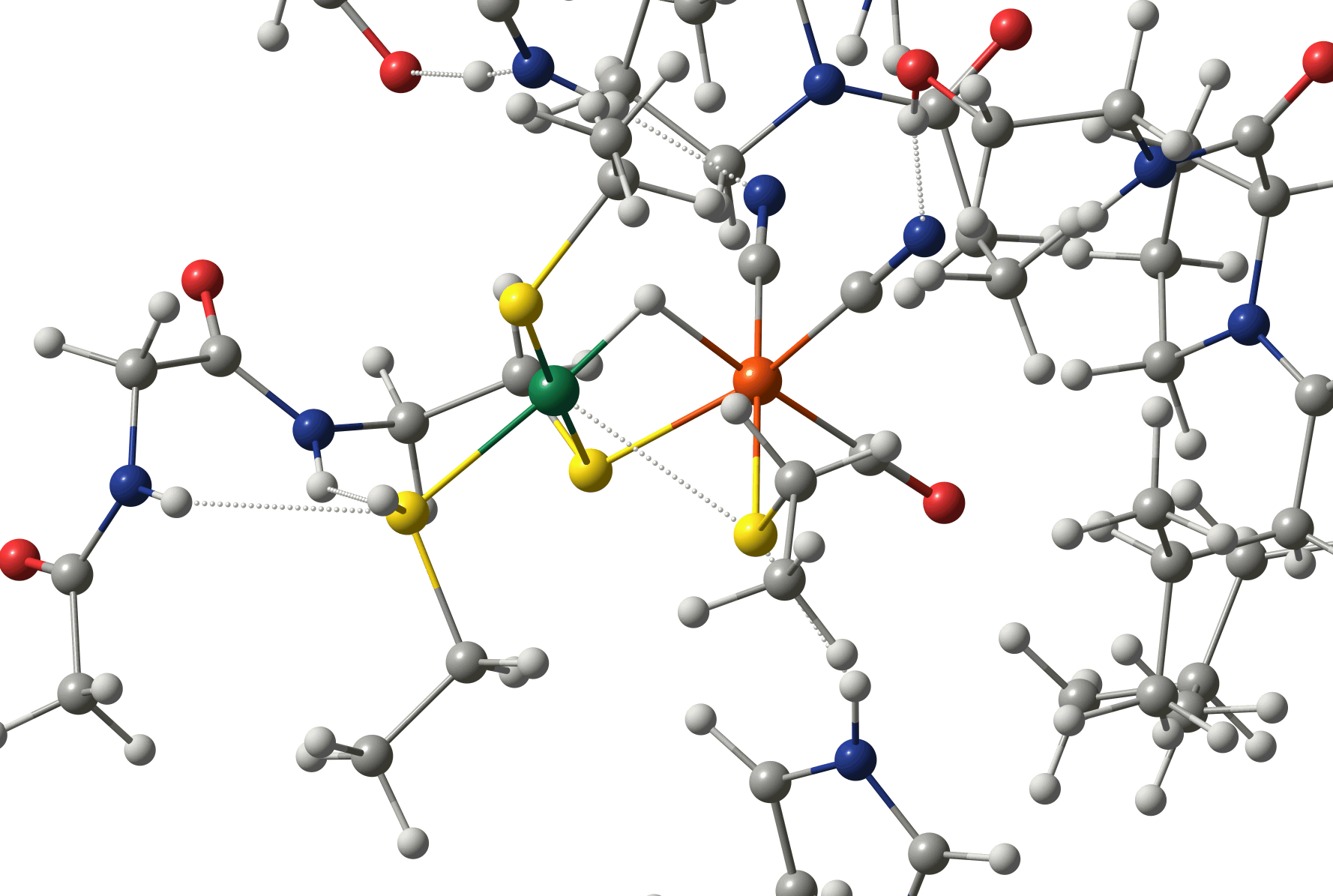

Supplement: Supplementary Movie 26 — Enzyme cluster model VI (H isotopomer), Singlet state, v = 588.10 cm-1 [file ncomms8890-s27.tif]

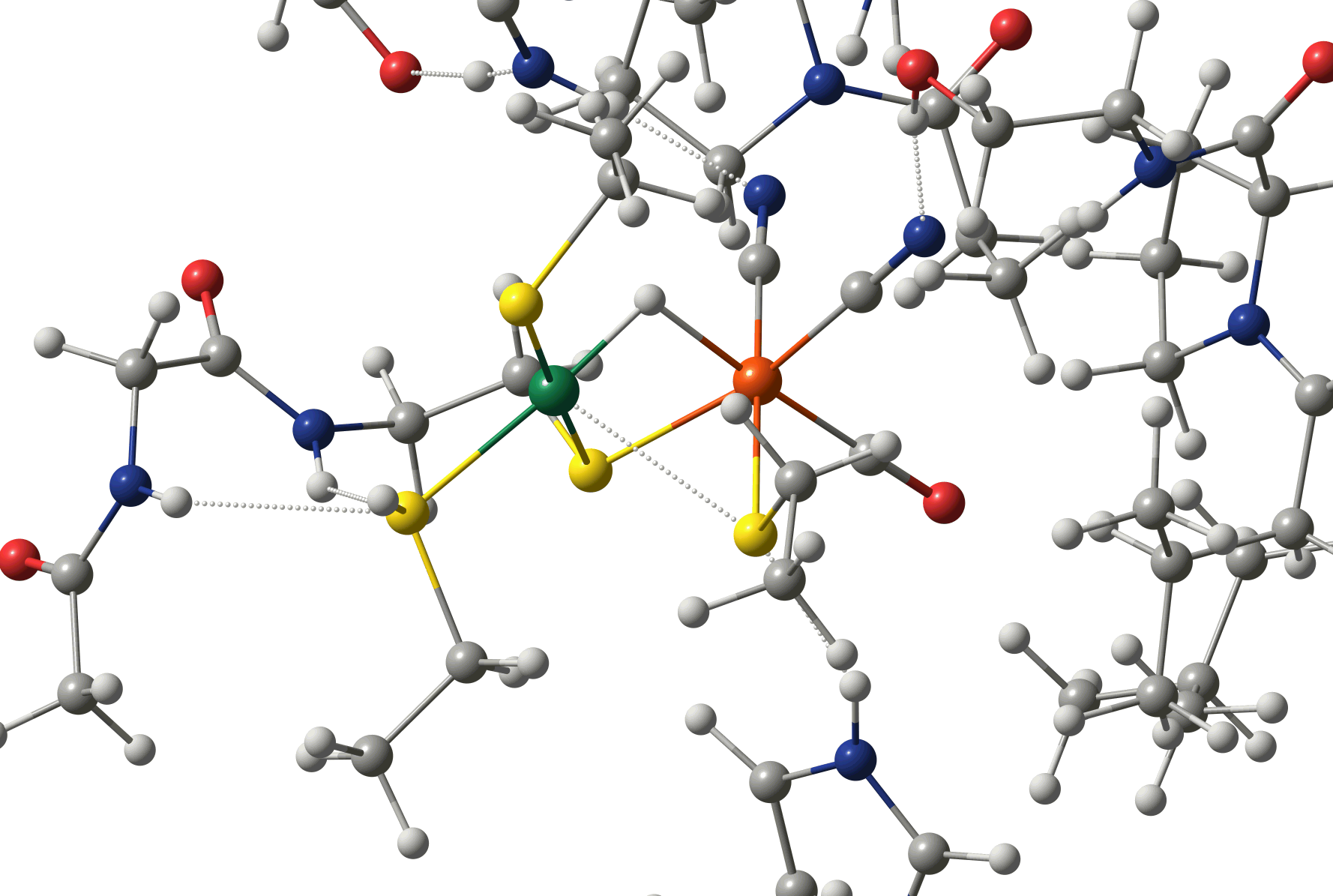

Supplement: Supplementary Movie 27 — Enzyme cluster model VI (H isotopomer), Singlet state, v = 614.86 cm-1 [file ncomms8890-s28.tif]

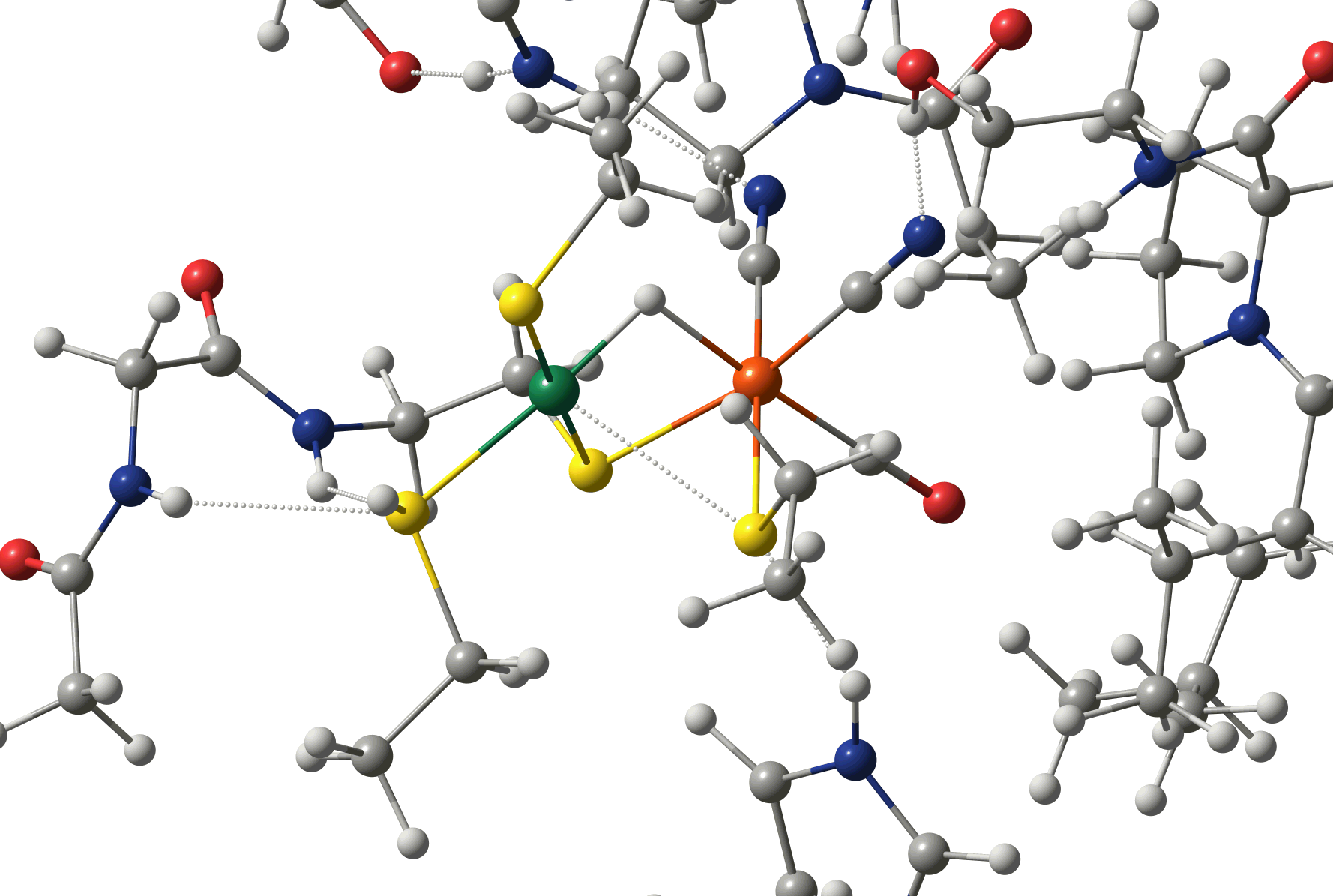

Supplement: Supplementary Movie 28 — Enzyme cluster model VI (H isotopomer), Singlet state, v = 692.53 cm-1 [file ncomms8890-s29.tif]

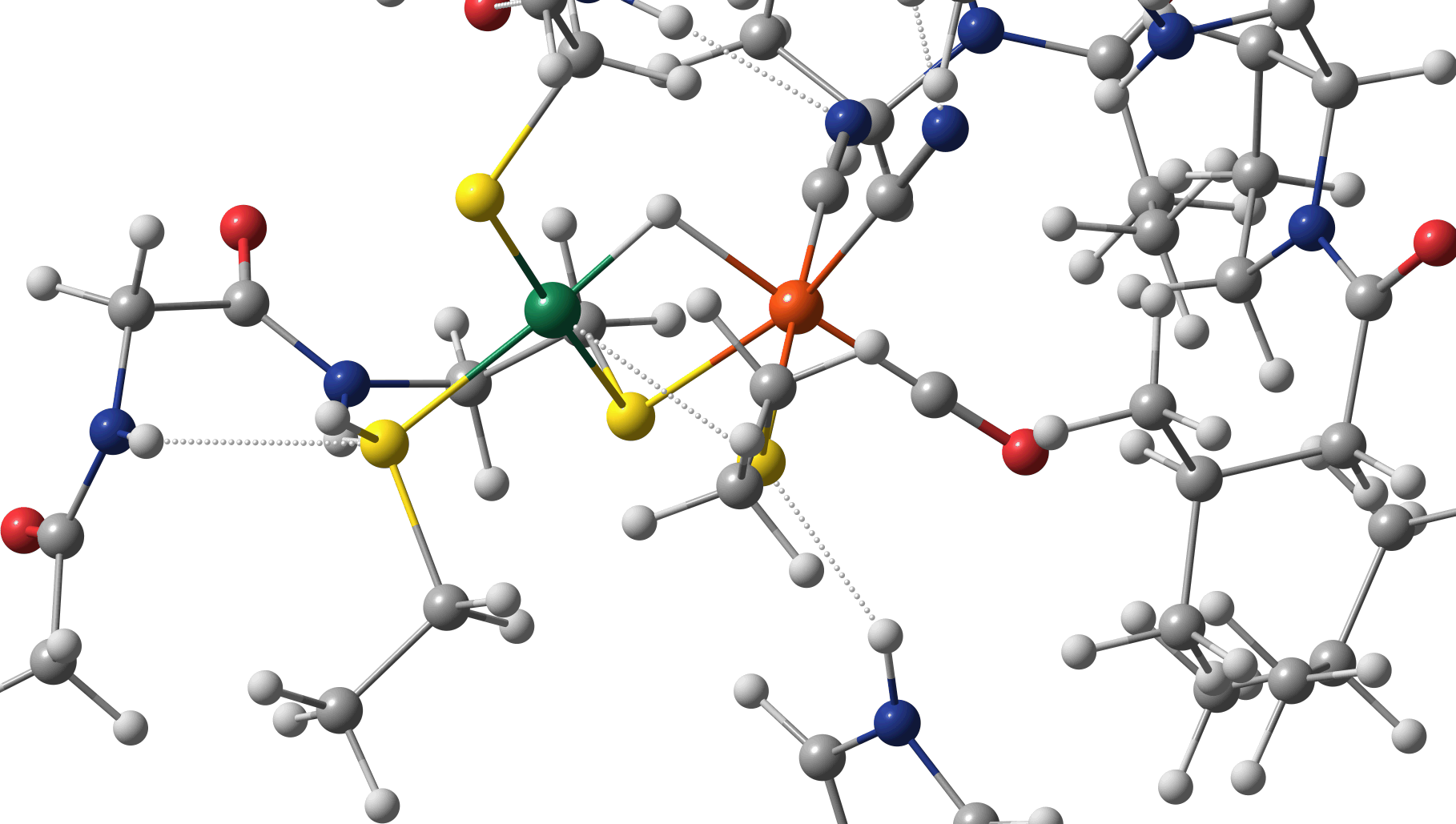

Supplement: Supplementary Movie 29 — Enzyme cluster model VI (D isotopomer), Singlet state, v = 406.20 cm-1 [file ncomms8890-s30.tif]

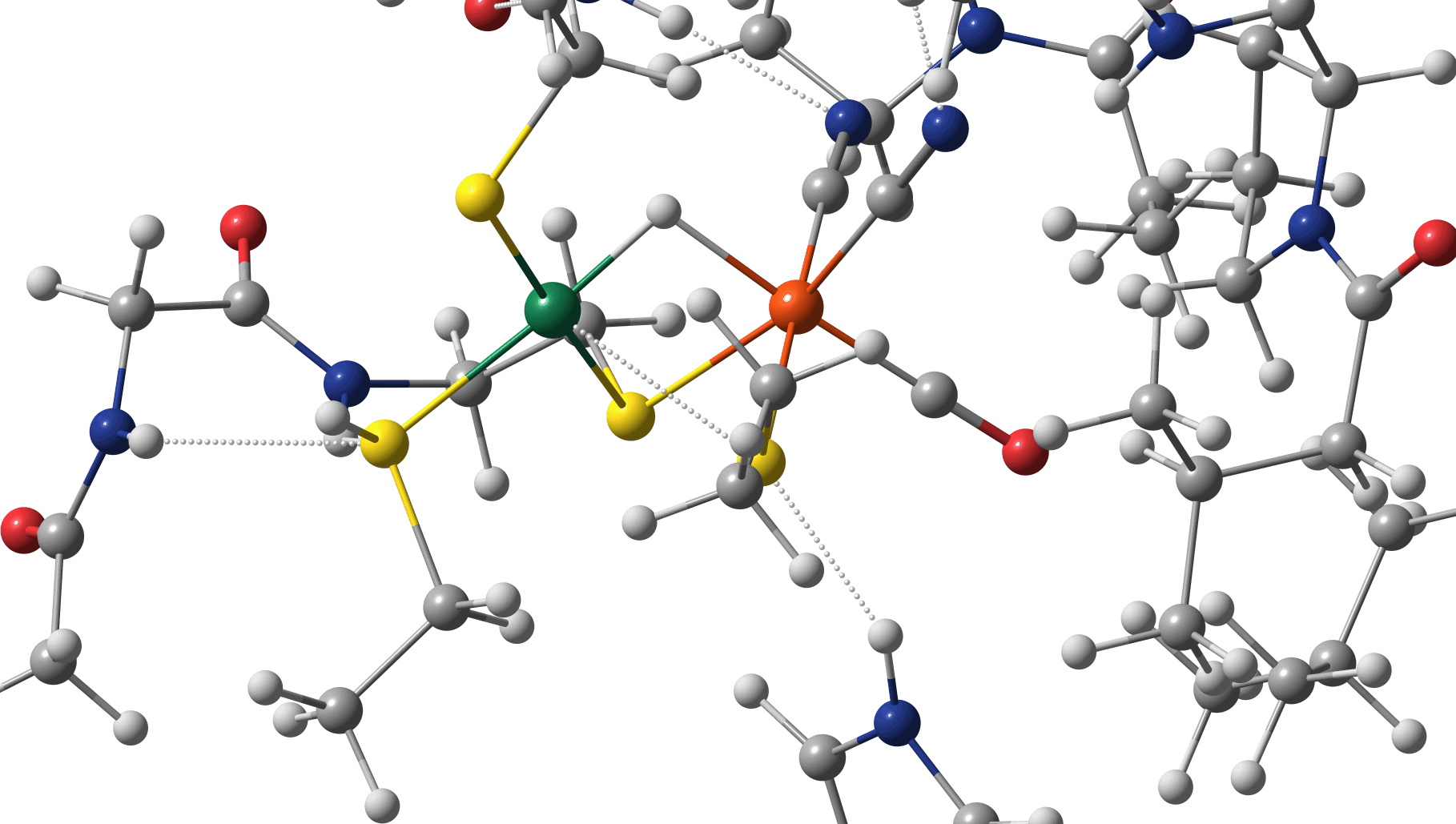

Supplement: Supplementary Movie 30 — Enzyme cluster model VI (D isotopomer), Singlet state, v = 427.43 cm-1 [file ncomms8890-s31.tif]

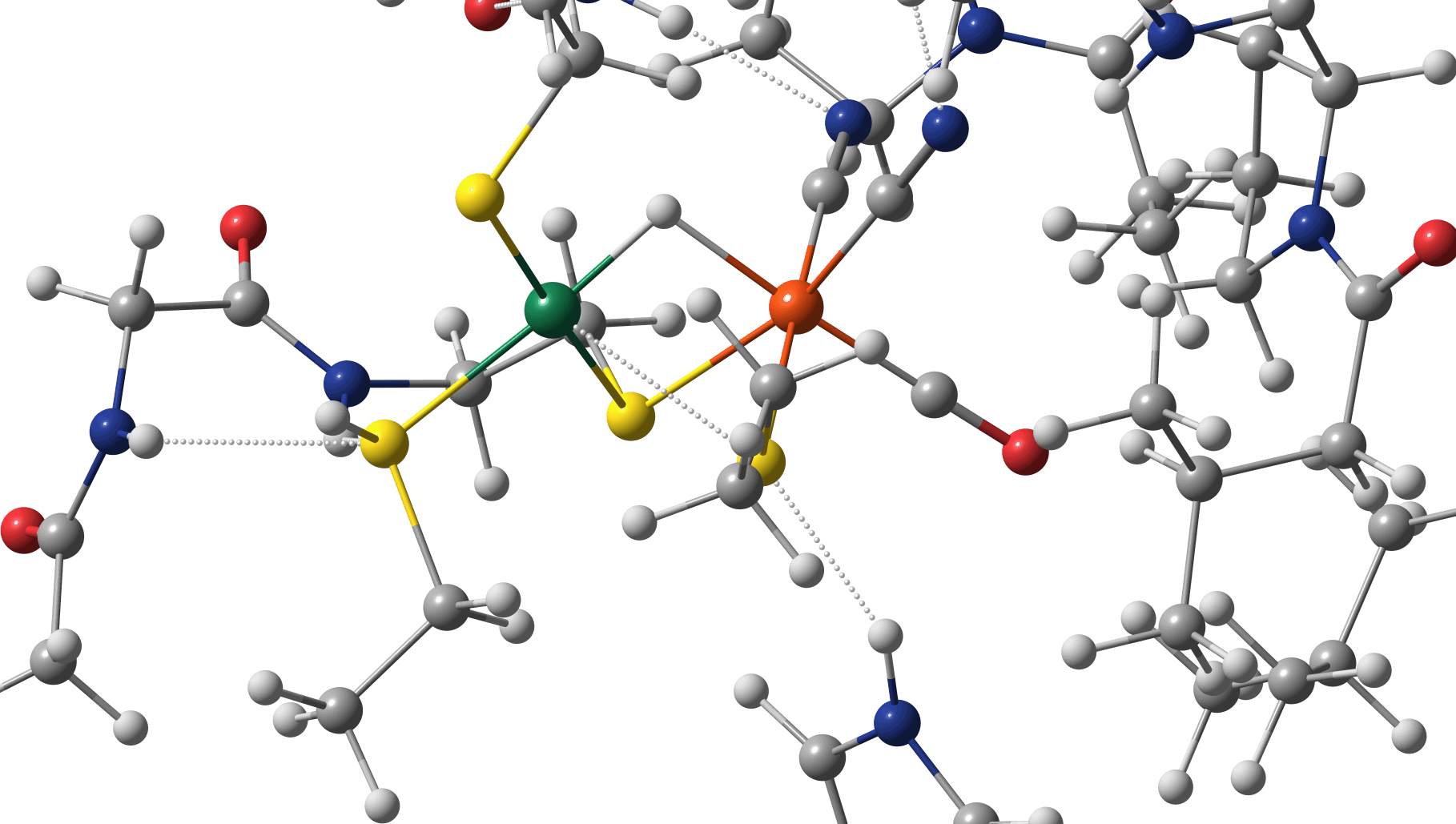

Supplement: Supplementary Movie 31 — Enzyme cluster model VI (D isotopomer), Singlet state, v = 440.82 cm-1 [file ncomms8890-s32.tif]

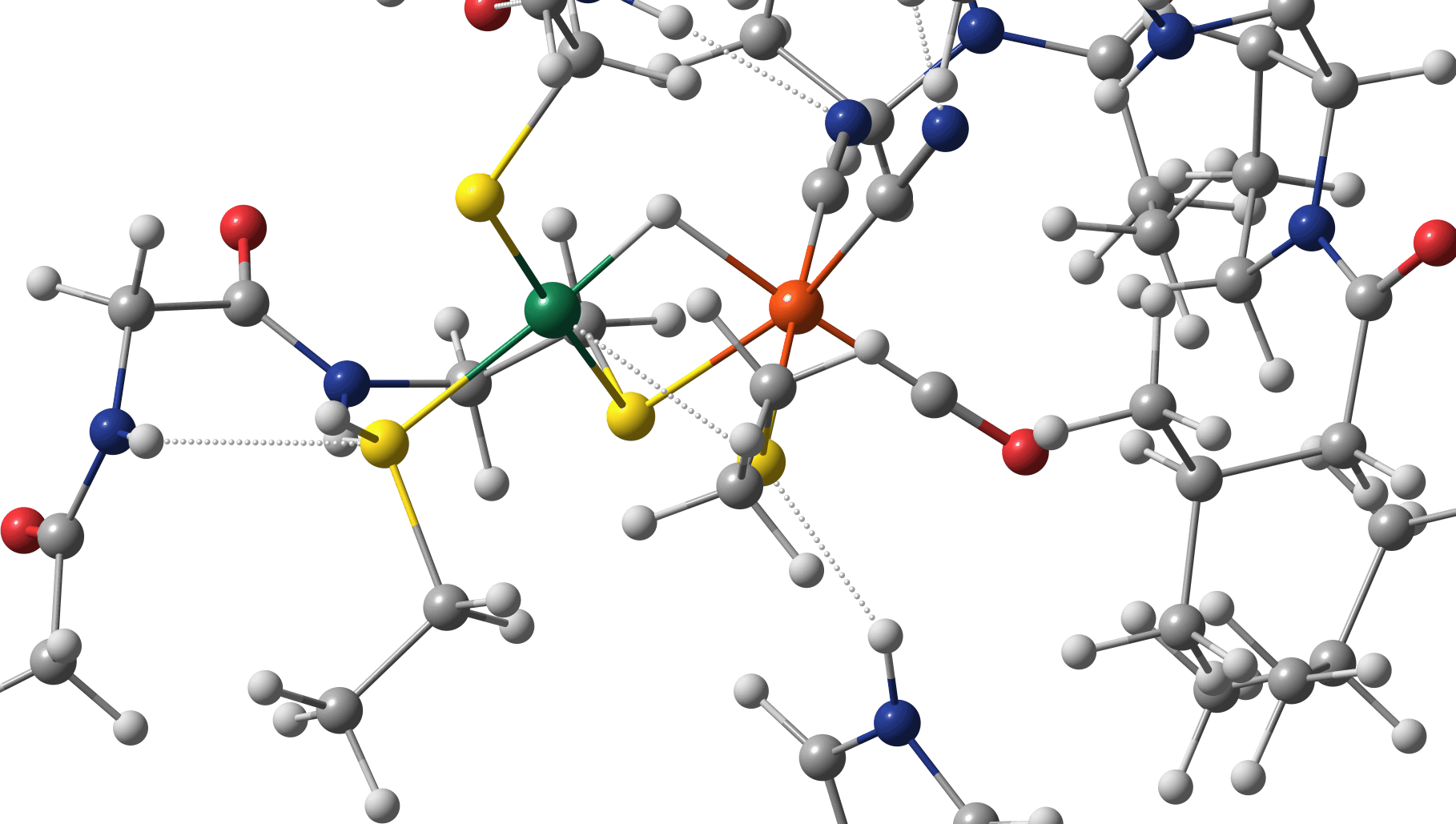

Supplement: Supplementary Movie 32 — Enzyme cluster model VI (D isotopomer), Singlet state, v = 457.11 cm-1 [file ncomms8890-s33.tif]

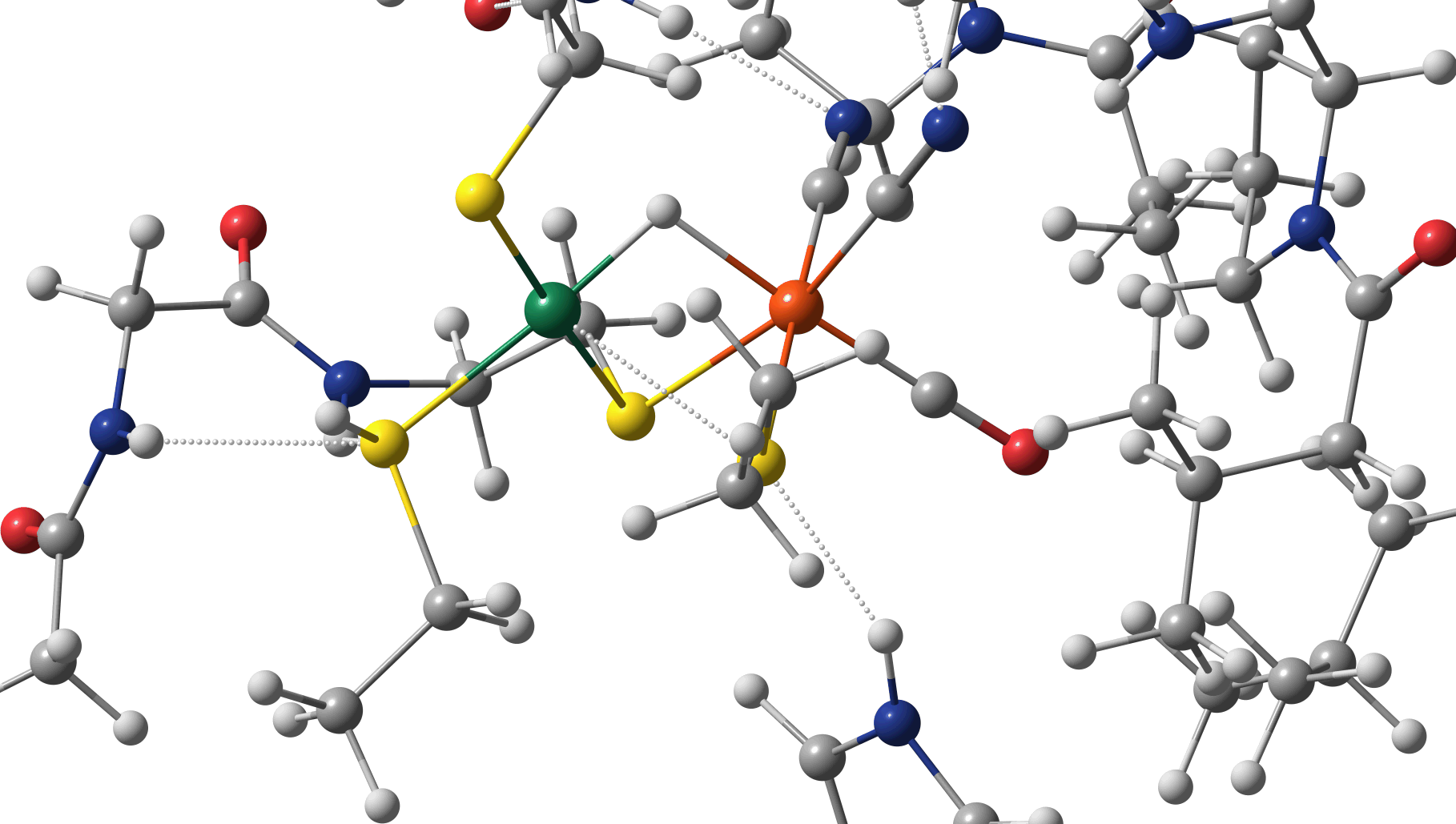

Supplement: Supplementary Movie 33 — Enzyme cluster model VI (D isotopomer), Singlet state, v = 500.43 cm-1 [file ncomms8890-s34.tif]

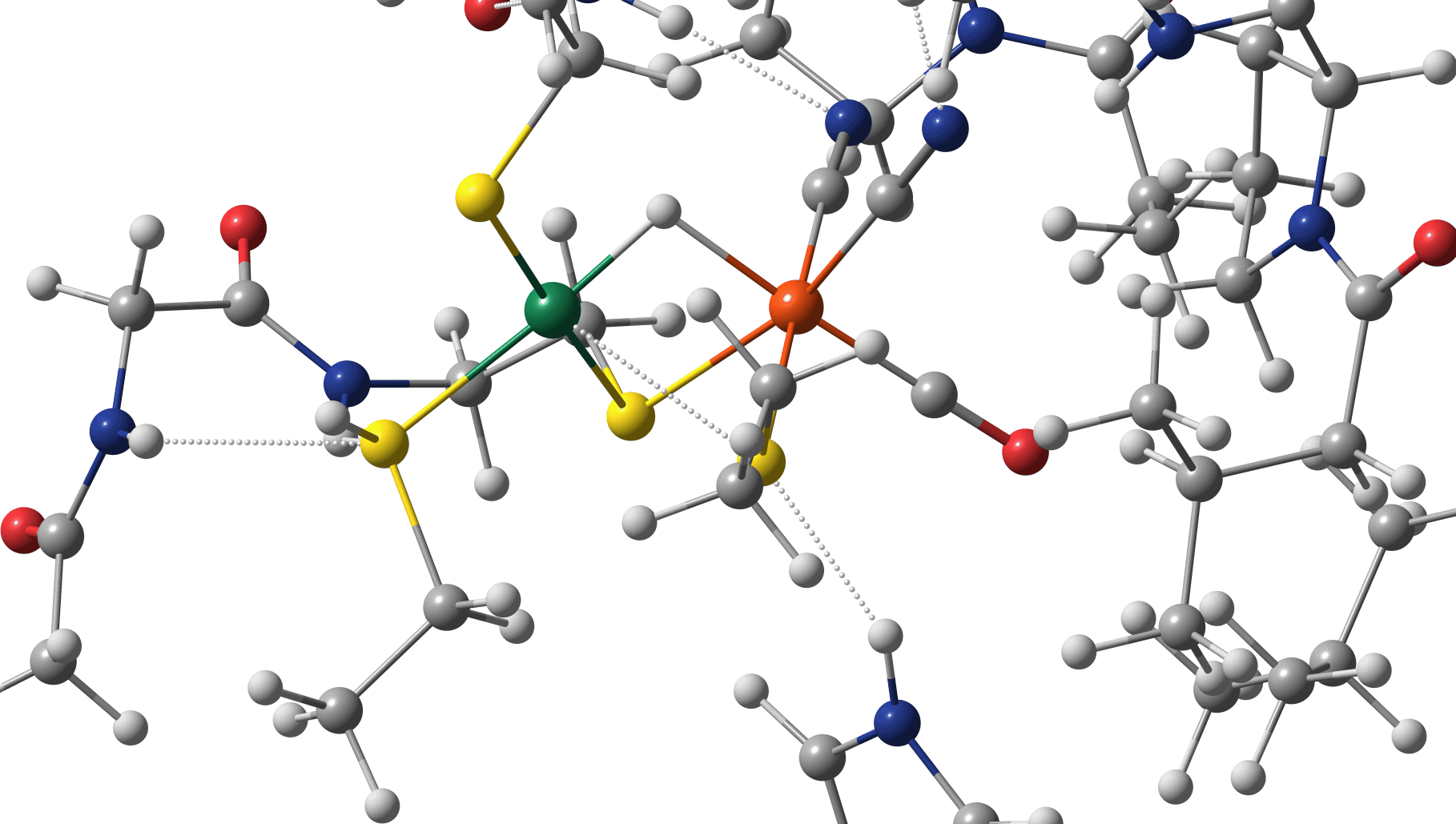

Supplement: Supplementary Movie 34 — Enzyme cluster model VI (D isotopomer), Singlet state, v = 508.69 cm-1 [file ncomms8890-s35.tif]

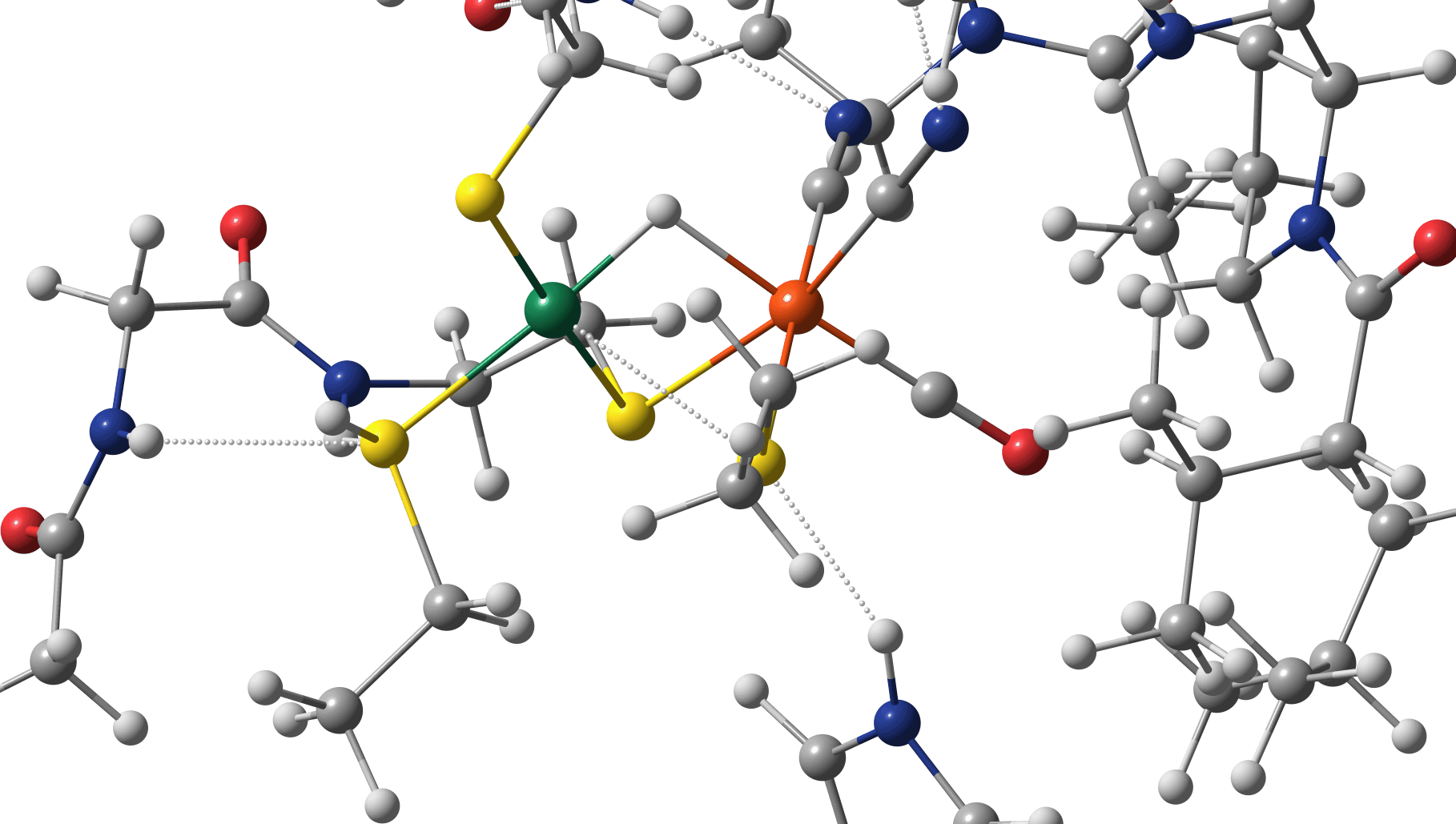

Supplement: Supplementary Movie 35 — Enzyme cluster model VI (D isotopomer), Singlet state, v = 545.50 cm-1 [file ncomms8890-s36.tif]

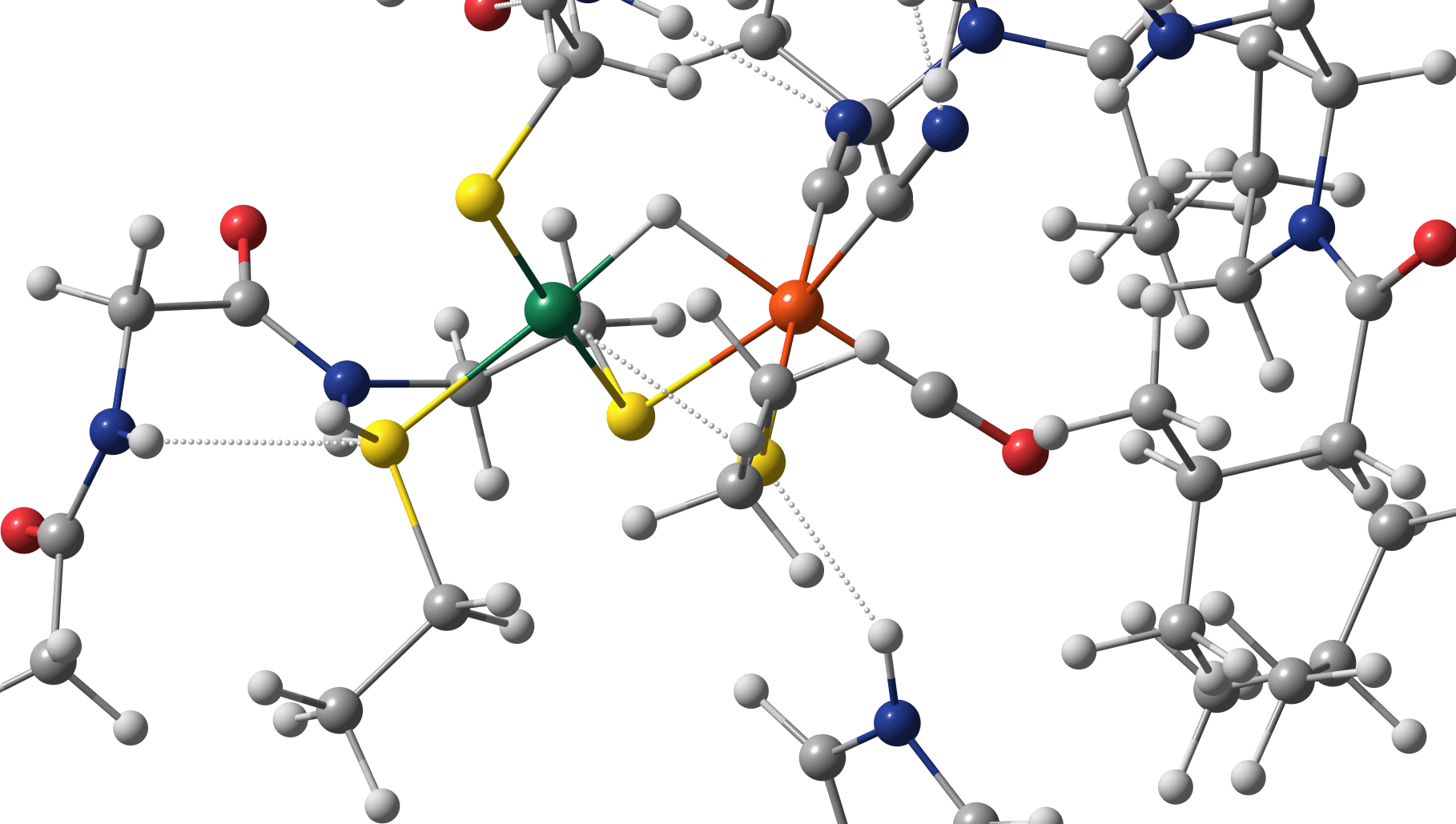

Supplement: Supplementary Movie 36 — Enzyme cluster model VI (D isotopomer), Singlet state, v = 597.65 cm-1 [file ncomms8890-s37.tif]

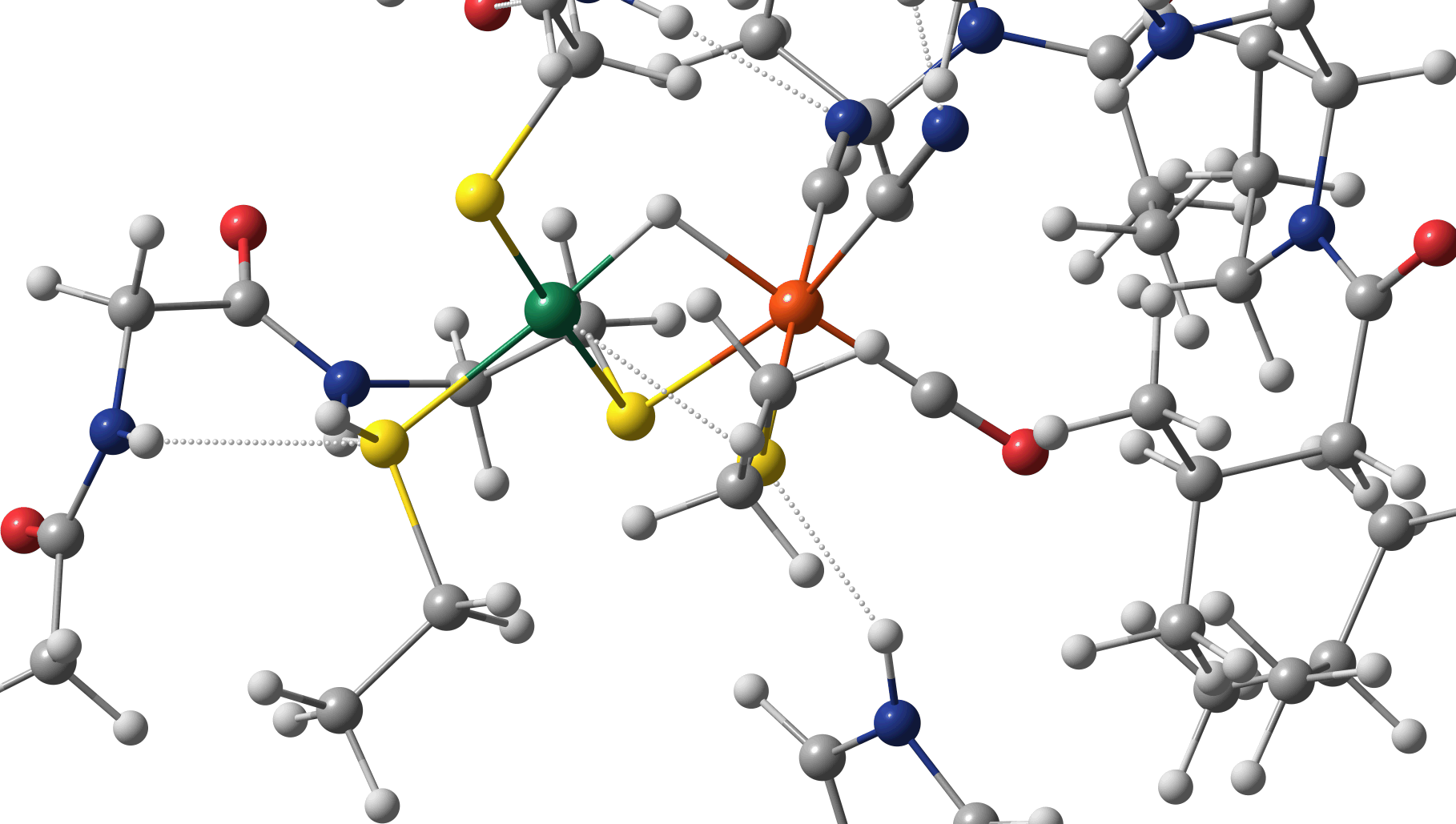

Supplement: Supplementary Movie 37 — Enzyme cluster model VI (D isotopomer), Singlet state, v = 613.77 cm-1 [file ncomms8890-s38.tif]
